# H3K27me3-rich genomic regions can function as silencers to repress gene expression via chromatin interactions

**DOI:** 10.1038/s41467-021-20940-y

**Published:** 2021-01-29

**Authors:** Yichao Cai, Ying Zhang, Yan Ping Loh, Jia Qi Tng, Mei Chee Lim, Zhendong Cao, Anandhkumar Raju, Erez Lieberman Aiden, Shang Li, Lakshmanan Manikandan, Vinay Tergaonkar, Greg Tucker-Kellogg, Melissa Jane Fullwood

**Affiliations:** 1 Cancer Science Institute of Singapore, National University of Singapore, Singapore, Singapore; 2 Department of Biological Sciences, National University of Singapore, Singapore, Singapore; 3 Cancer and Stem Cell Biology Programme, Duke-NUS Medical School, Singapore, Singapore; 4 Department of Cancer Biology, Perelman School of Medicine, University of Pennsylvania, Philadelphia, PA USA; 5 Institute of Molecular and Cell Biology, Agency for Science, Technology and Research (A*STAR), Proteos, Singapore; 6 Department of Molecular and Human Genetics, Baylor College of Medicine, Houston, TX USA; 7 Department of Physiology, Yong Loo Lin School of Medicine, National University of Singapore, Singapore, Singapore; 8 Computational Biology Programme, National University of Singapore, Singapore, Singapore; 9 School of Biological Sciences, Nanyang Technological University, Singapore, Singapore

**Keywords:** Cancer models, Gene regulation, Chromatin structure, Epigenetics, Transcription

## Abstract

The mechanisms underlying gene repression and silencers are poorly understood. Here we investigate the hypothesis that H3K27me3-rich regions of the genome, defined from clusters of H3K27me3 peaks, may be used to identify silencers that can regulate gene expression via proximity or looping. We find that H3K27me3-rich regions are associated with chromatin interactions and interact preferentially with each other. H3K27me3-rich regions component removal at interaction anchors by CRISPR leads to upregulation of interacting target genes, altered H3K27me3 and H3K27ac levels at interacting regions, and altered chromatin interactions. Chromatin interactions did not change at regions with high H3K27me3, but regions with low H3K27me3 and high H3K27ac levels showed changes in chromatin interactions. Cells with H3K27me3-rich regions knockout also show changes in phenotype associated with cell identity, and altered xenograft tumor growth. Finally, we observe that H3K27me3-rich regions-associated genes and long-range chromatin interactions are susceptible to H3K27me3 depletion. Our results characterize H3K27me3-rich regions and their mechanisms of functioning via looping.

## Abstract (web summary)

Mechanisms underlying gene repression and silencers remain poorly understood. Here the authors investigate the role of H3K27me3-rich regions in the genome, as defined from clusters of H3K27me3 peaks, in regulating gene expression via looping.

## Introduction

The 3-dimensional organization of our genomes is important for gene regulation^1–3^. The genome is organized into large Topologically-Associated Domains (TADs) and chromatin interactions. Gene transcription is controlled by transcription factors (TFs) that bind to enhancers and promoters to regulate genes^4^. TFs can bind to proximal enhancers in the genome, and enhancers distal to genes can loop to gene promoters via chromatin interactions to activate gene expression^3^. Cancer cells show altered chromatin interactions^2,3^ including altered chromatin loops to key oncogenes such as *TERT*^5^.

By contrast, mechanisms for gene repression are much less well understood. Silencers are regions of the genome that are capable of silencing gene expression. Silencers have been shown to exist in the human genome, but are less well characterized than enhancers. Until now, there are only a few known experimentally validated silencers that have been demonstrated to repress target genes in vitro, such as the human synapsin I gene^6^, the human *BDNF* gene^7^ and the human *CD4* gene^8,9^ (experimentally validated silencer examples are discussed in Supplementary Table 1). The reason for the paucity of known silencers in the literature is that methods to identify human silencer elements in a genome-wide manner are only starting to be developed now. Moreover, the mechanism by which silencers can regulate distant genes is still uncharacterized. Distant silencers are thought to loop over to target genes to silence them^10,11^, and this mechanism has been demonstrated in studies of polycomb-mediated chromatin loops in *Drosophila*^12^ and in mice^13^. but no such examples have been characterized to date in humans.

Polycomb Group (PcG) proteins including Polycomb Repressive Complexes, PRC1 and PRC2 are widely recognized to mediate gene silencing of developmental genes^14^. During the development process, PRC1 and PRC2 have the ability to orchestrate genome architecture and repress gene expression^15^. There are two different types of genomic domains: active domains and repressive domains, which regulate gene expression and establish cellular identity. Genes involved in cell self-renewal are contained within the active domains which are governed by super-enhancers, while genes specifying repressed lineage are organized within chromatin structures known as PcG domains^16^. Moreover, intact PcG domains have been shown to be necessary to maintain the chromatin interaction landscape^17,18^. However, the mechanisms of PcG domain formation and PcG proteins recruitment are not fully characterized yet^19^, which makes finding silencers more difficult.

PcG domains are marked by H3K27me3, which is deposited by the catalytic component of PRC2 complex, mainly Enhancer of Zeste Homolog 2 (EZH2) and sometimes EZH1^20^. H3K27me3 marks are associated with gene repression for cell type-specific genes. Unlike H3K9me3 which remains silenced all the time and prevents multiple TFs from binding^21^, H3K27me3 still allows these genes to be activated through TF binding in a different cell state^22^. H3K27me3 is known to be a characteristic of silencers^18,23^. Although large blocks of H3K27me3-marked loci have been observed in previous studies^24–26^, their roles in chromatin loops and consequent regulatory actions were not explored in these manuscripts.

Recently, several studies have proposed methods to identity silencer elements in a genome-wide manner. Huang et al. defined silencers using the correlation between H3K27me3-DNase I hypersensitive site (DHS) and gene expression^27^. At the same time, Jayavelu et al. used a subtractive analysis approach to predict silencers in over 100 human and mouse cell types^28^. Moreover, Pang and Snyder identified silencers through an innovative “ReSE screen” which screened for genomic regions that can repress caspase 9 expression upon apoptosis induction^29^. Ngan et al. characterized silencers in mouse development through PRC2 Chromatin Interaction Analysis with Paired-End Tag sequencing (ChIA-PET) in mouse embryonic stem cells. They concluded that PRC2-bound looping anchors function as transcriptional silencers suggesting that we can identify silencers through investigating chromatin interactions^13^.

However, there is no consensus yet in terms of how to identify silencers. Notably, each of these methods identify different genomic regions as silencers, raising the question of whether there may be different classes of silencers. Moreover, current methods for identifying silencers are laborious and require complicated bioinformatics analyses and/or genome-wide screening (Supplementary Table 2, “comparison of different human silencer identification methods”). A simple, easy to perform method to identify silencers in the genome in a high-throughput manner would be ideal. Further investigation is needed to understand whether there are different classes of silencers and to characterize the roles of silencers in the genome.

The term “super-enhancer”^30^ has been used to describe clusters of H3K27ac peaks which show very high levels of H3K27ac or other transcription-associated factors such as mediators as determined from ChIP-seq data. Super-enhancers have high levels of chromatin interactions to target genes^31^, and are associated with oncogenes in cancer cells^32^ and cell fate-associated genes in embryonic stem cells^30^. While more research needs to be done to determine if super-enhancers are a distinctly different entity from enhancers, super-enhancers are thought as strong enhancers, and the definition has been useful in identifying genes important for cell-type specification^33^.

We reason that “super-silencers” or “H3K27me3-rich regions (MRRs)” from clusters of H3K27me3 peaks in the genome can be identified through H3K27me3 ChIP-seq, as how super-enhancers are defined. We hypothesize that H3K27me3-rich regions may be a useful concept in identifying genomic regions that contain silencers which can repress target genes either in proximity or via long-range chromatin interactions. The target genes may be tumor suppressors in cancer cells, and also cell fate-associated genes that need to be turned off for differentiation to occur.

Here, we show that MRRs can be identified using H3K27me3 ChIP-seq data. MRRs show dense chromatin interactions connecting to target genes and to other MRRs. CRISPR excision of two examples of looping silencers leads to gene up-regulation, indicating they are indeed *bona fide* silencers. CRISPR excision leads to changes in chromatin loops, histone modifications, and cell phenotype including cell adhesion, growth and differentiation. Initial histone modification states predict changes in chromatin loops. Finally, EZH2 inhibition leads to changes in chromatin interactions and histone modifications at MRRs, and MRR-associated gene up-regulation. Taken together, silencers identified from clustering H3K27me3 peaks regulate key epigenomic, transcriptomic and phenotypic events in cells.

## Results

### Identification and characterization of H3K27me3-rich regions (MRRs) in the human genome

**Fig. 1:**
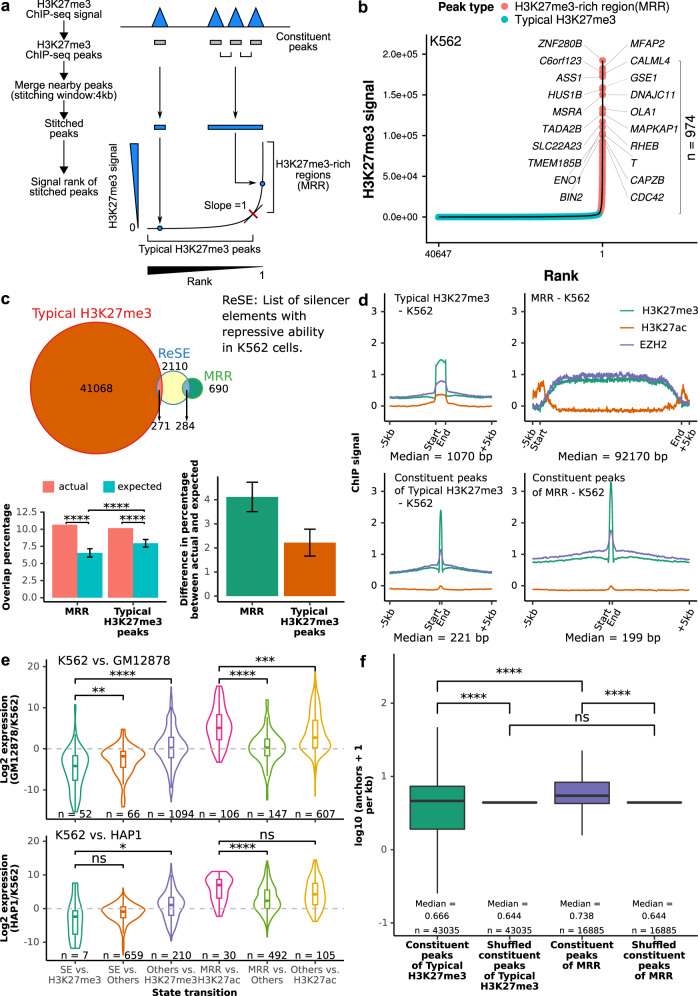
Definition of H3K27me3-rich regions (MRRs) and their characterization. **a** Schematic figure of MRR calling. More details can be found in the Methods section. Constituent peaks, peaks that are stitched during the process of merging peak. **b** H3K27me3-rich regions (MRRs) and typical H3K27me3 peaks in K562 and their associated genes. A representative overlapping gene from each of the top 10 MRRs was shown. **c** Overlap of MRR and typical H3K27me3 with ReSE list^29^. The Venn diagram shows the observed overlap between our MRR (H3K27me3-rich region)/typical H3K27me3 peaks and the ReSE list. Left barplot: The barplots show the percentage of elements in ReSE list that overlap with MRR/typical H3K27me3 peaks. Actual, observed overlap percentage (*n* = 1); expected, expected overlap percentage generated by random shuffling (*n* = 1000). Error bars indicate mean values ± SD. One-sided one sample *t*-test was used to test whether the expected are lower than the actual. Right barplot: The difference between actual and expected percentage. **d** ChIP-seq signal on typical H3K27me3, MRR, constituent peaks of typical H3K27me3 peaks, and constituent peaks of MRR regions in K562. Peaks are scaled to the same median length of peaks in typical H3K27me3 (1070 bp), MRR (92170 bp), constituent peaks of typical H3K27me3 (221 bp), or constituent peaks of MRRs (199 bp), and the plot expanded by 5 kb on both sides of the peak. **e** Expression changes associated with different peaks between different cells. K562 vs. GM12878/K562 vs. HAP1 cell lines used in the comparison. Two-tailed Wilcoxon test *p* values are as indicated. **f** Constituent peaks of MRRs have more Hi–C interactions compared to the constituent peaks of typical H3K27me3. The shuffled peaks are generated by expanding the midpoint of each constituent peaks to the median length of all the constituent peaks, and then followed by random genomic region shuffling. Two-tailed Wilcoxon test was used. Box and whiskers plot: whiskers were extended to the furthest value that is no more than 1.5 times the inter-quartile range. The boxes represent the 25th percentile, median, and 75th percentile. *p < = 0.05; **p < = 0.01; ***p < = 0.001; ****p < = 0.0001; ns, *p* > 0.05.

We identified highly H3K27me3-rich regions (MRRs) from cell lines using H3K27me3 ChIP-seq data^34^ in the following manner: we first identified H3K27me3 peaks, then clustered nearby peaks, and ranked the clustered peaks by average H3K27me3 signals levels. The top clusters with the highest H3K27me3 signal were called as “H3K27me3-rich regions” (MRRs) and the rest were called as “typical H3K27me3” regions (Fig. 1a, b). The peaks that were merged together during this process were called constituent peaks. This method is similar to how super-enhancers were defined^30,35^. Recently, Pang and Snyder identified a list of silencer elements in K562 cells using a lentiviral screening system called ReSE^29^. We overlapped our list of MRRs in K562 with the list of silencers that identified by ReSE and found that 10.66% of ReSE silencer elements overlap with our MRRs (Fig. 1c). This overlap percentage of 10.66% between our MRR and the ReSE silencer elements is significantly higher when compared to random expectation (Fig. 1c). Although typical H3K27me3 peaks also have more overlap when compared with expectation, the differences in the percentage between actual and expected overlap percentage are larger for MRR (Fig. 1c). This indicated that MRRs can be used to identify silencers in the genome. While the overlap percentage between our MRR and ReSE silencer elements is higher than random expectation, it is still relatively low compared with the total number of ReSE elements, which could be because ReSE elements contain other types of silencers such as DNA hypomethylated regions.

The number of constituent peaks and overlapping genes at MRRs is larger than typical H3K27me3 peaks (Supplementary Fig. 1a, b). Considering the differences in the lengths of MRRs and typical H3K27me3 peaks, we used constituent peaks of MRRs and typical H3K27me3 peaks to study CpG methylation and gene features. The results showed that the constituent peaks of MRRs and typical H3K27me3 peaks mostly overlap with inter CpG island methylation (Supplementary Fig. 1c) and the intronic regions of genes (Supplementary Fig. 1d).

Many MRR-overlapping genes in different cell lines are known or predicted tumor suppressor genes^36^ (Supplementary Fig. 1e, Supplementary Data 1 and 2). For example, *NPM1*, the most commonly mutated gene in leukemia^37–40^, overlaps with an MRR in the leukemic cell line K562. *FAT1*, which is frequently mutated in chronic lymphocytic leukemia (CLL) and can act as a tumor suppressor through inhibiting Wnt signaling^41,42^, also overlaps with an MRR in K562. Gene ontology analysis showed that MRR-related genes are enriched in developmental and differentiation processes, while genes associated with typical H3K27me3 peaks are enriched in cell metabolism and transportation processes (Supplementary Fig. 1f, g). These results suggested that MRR may regulate important genes related to development and tumorigenesis.

ChIP-seq signals of EZH2 showed high correlation with H3K27me3 signal at typical H3K27me3, MRRs, constituent peaks of typical H3K27me3 and constituent peaks of MRRs, which is consistent with EZH2’s role in H3K27me3 mark deposition (Fig. 1c; Supplementary Fig. 1h, i). Notably, the constituent peaks of MRRs had higher H3K27me3 and EZH2 signals than the constituent peaks of typical H3K27me3 peaks. This suggests that there are genomic regions with higher level of H3K27me3 and EZH2 compared with others, and they can be found in MRRs. In addition, the ChIP-seq profiles of SUZ12 and BMI1 are also higher in the constituent peaks of MRRs, suggesting that these regions may be targeted by PRC1 and PRC2 complex (Supplementary Fig. 1j, k).

MRRs were different in different cell lines, where a gene can overlap with different types of peaks (Supplementary Fig. 1l–n). For example, the cadherin-like coding gene *CPED1* is covered by a broad MRR in GM12878, but overlaps with a super-enhancer in K562 (Supplementary Fig. 1l). Conversely, the gene for *DENND2D* is associated with an MRR but overlaps with a super-enhancer in GM12878 (Supplementary Fig. 1l). However, *CPED1* is not covered by super-enhancer or MRR in HAP1 cells (Supplementary Fig. 1m, n). In addition, most MRRs were unique to individual cell lines (Supplementary Fig. 1o).

Analysis of cell line expression data showed that genes which are MRR-associated in one cell line, but H3K27ac peak-associated in a second cell line were upregulated in the second cell line, while genes that are super enhancer-associated in one cell line but are H3K27me3 peak-associated in a second cell line were down-regulated in the second cell line (Fig. 1e). This observation is consistent with previously identified elements with dual function in both enhancing and silencing in mouse, human^42^, and *Drosophila*^43^. The expression fold changes between repressive and active state are higher than those genes that merely lost MRR or SE (Fig. 1e; MRR vs. others and SE vs. others) or gained H3K27ac or H3K27me3 (Fig. 1e; others vs. H3K27ac and others vs. H3K27me3), respectively. Further, genes whose expression were more cell line-specific were associated with more MRRs than those genes with lower expression specificity (Supplementary Fig. 1p). The uniqueness and specificity of MRRs suggested they might be primed for specific regulation in different contexts.

We overlapped MRRs with high-resolution in situ Hi-C data^44^, and found that constituent peaks of MRRs had a higher density of chromatin interactions than the constituent peaks of typical H3K27me3 peaks in both K562 and GM12878 (Fig. 1f; Supplementary Fig. 1q, r). The involvement of chromatin interactions in MRRs was similar to super-enhancers compared with typical enhancers^45^, which suggested that chromatin interactions might be important within regions rich in histone modification marks.

In summary, we defined MRRs using H3K27me3 ChIP-seq peaks, and showed that MRRs might be involved with specific gene repression related to development, differentiation and cancer via chromatin interactions.

### H3K27me3-rich regions (MRRs) preferentially associate with MRRs in the human genome via chromatin interactions

**Fig. 2:**
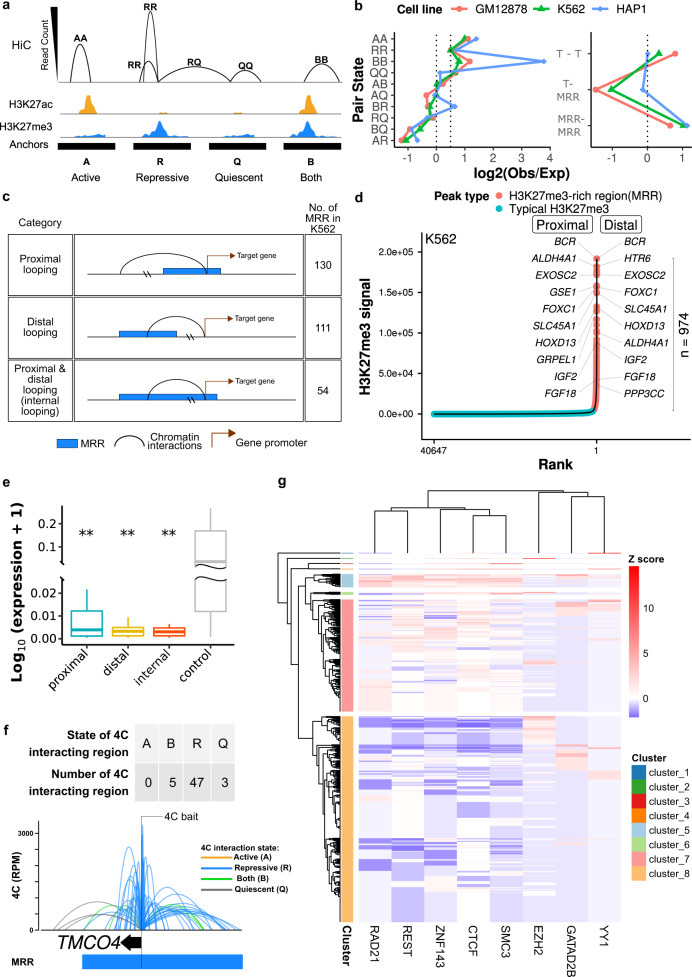
H3K27me3-rich regions (MRRs) preferentially associate with MRRs in the human genome via chromatin interactions. **a** Schematic plot of how different categories of Hi–C interactions are defined. More details can be found in the Method section. **b** Observed/expected ratio of Hi–C interactions in different categories. Left: categories of chromatin pair states. Right: T (typical H3K27me3) or MRR peaks. The expected interactions are calculated from the marginal distributions of different anchors. **c** Different categories of MRR associated with genes. **d** H3K27me3-rich regions (MRRs) and typical H3K27me3 peaks in K562 and their associated genes through chromatin interactions. Peaks overlapping with Hi–C interactions are labeled with associated genes: for peaks labeled “proximal”, the gene TSS and peak occupy the same Hi-C anchor; “distal” peaks are connected to the gene via Hi–C interactions. **e** Expression of genes that are associated with MRR in proximal, distal, and internal category in K562 cells. The three categories are described in **c**. Two-tailed Wilcoxon test was used to compare proximal/distal/internal category with the control. Proximal (*n* = 50, *p* = 0.0084), distal (*n* = 41, *p* = 0.018), internal (*n* = 49, *p* = 0.0077), control (*n* = 46). Box and whiskers plot: whiskers were extended to the furthest value that is no more than 1.5 times the inter-quartile range. The boxes represent the 25th percentile, median, and 75th percentile. *p < = 0.05; **p < = 0.01; ***p < = 0.001; ****p < = 0.0001; ns, *p* > 0.05. **f** Example of 4C at the *TMCO4* gene promoter bait showing extensive internal looping within an MRR in K562. The colors of 4C interactions are based on the distal interacting regions to the 4C bait. Blue: repressive; orange: active; green: both; gray: quiescent. The state of the 4C bait is labeled by text. Each ChIP-seq track contains ChIP signal and peaks. TE, typical enhancer; SE, super-enhancer; T, typical H3K27me3; MRR, H3K27me3-rich region. **g** Heatmap of transcription factors binding enrichment at interacting regions of MRRs. Each row representing an interacting region of MRRs. The number of overlapping transcription factor peaks at interacting regions are normalized to Z score per transcription factor. Red colors indicate more binding events.

We assigned chromatin states at Hi-C interaction anchors using H3K27me3 and H3K27ac peaks: active (A) anchors overlap with H2K27ac peaks, repressive (R) anchors overlap with H3K27me3 peaks, bivalent (B) anchors overlap with both H3K27me3 and H3K27ac peaks, and quiescent (Q) anchors overlap with neither peak (Fig. 2a). We further defined the chromatin state pair of an interaction as the chromatin states of its anchors and calculated the proportion of different chromatin interactions in the Hi-C data (Fig. 2b, “Obs”). Next, we calculated the expected proportion of interactions for each state pair under a homogeneous model (Fig. 2b, “Exp”), and compared those expectations to the actual number of observations (Fig. 2b, log_2_(Obs/Exp) on the *x*-axis). If the observed proportion of a certain category of interactions were more frequently seen, the log_2_(Obs/Exp) value would be positive; conversely, if a certain category was depleted, the log_2_(Obs/Exp) value would be negative.

Interactions between anchors of the same state (AA, RR, and BB) were more likely to interact with each other, while interactions with vastly different chromatin state pairs (e.g., AR, BQ) were less likely to interact (Fig. 2b, left), regardless of different cell lines. When grouped into typical H3K27me3 peaks (T) versus high H3K27me3 regions or MRRs (MRR), the high H3K27me3 regions showed a preference for interactions with other MRRs (Fig. 2b, right). In keeping with A/B chromatin compartments of the nucleus, this “like-like” preference indicated that loci of similar chromatin states were more prone to interact with each other.

To further explore the potential regulatory role of MRRs in chromatin interactions, we identified the subset of MRR-anchored interactions where at least one anchor peak overlapped a gene transcription start site, and grouped them according to whether the MRR anchor was proximal or distal to the TSS anchor (Fig. 2c, d; Supplementary Fig. 2a–f, g; Supplementary Data 1; examples of genes can be found in Supplementary Fig. 2i–2l). Both proximal and distal gene looping occur for MRR-anchored interactions, but some MRRs are large enough that both anchors occur in the same MRR. While proximal looping genes are a subset of the genes within MRRs, distal looping genes are only identified by chromatin interactions (Fig. 2d, right panel). The expression levels of genes that are proximally, distally, or internally associated with MRR are lower than randomly sampled genes that are involved in chromatin interactions (Fig. 2e; Supplementary Fig. 2h). The difference in gene expression levels between proximal, distal, and internal categories is not significant, suggesting that distal looping by MRRs is associated with reduced gene expression to a similar extent as proximal regulation by MRRs (Fig. 2e). There is no significant difference between proximal, distal and internal categories, thus showing that genes regulated by distal looping may be silenced to the same extent as genes proximal to MRRs. This indicated the importance of long-range looping in mediating silencing between distal regulatory elements and gene promoters. The top-ranking MRRs are often involved in extensive internal looping (Supplementary Fig. 2k–l). Gene ontology analysis showed that MRR-associated genes in the context of chromatin interactions are involved in developmental and differentiation processes (Supplementary Fig. 2m).

In order to validate the “like-like” preference of chromatin interactions, we performed Circular Chromosome Conformation Capture (4C) experiments on selected loci at MRR to investigate the associated chromatin interactions in a comprehensive and high-resolution manner. We annotated the interactions based on the chromatin state of the anchor distal from the bait in K562 (Fig. 2f and Supplementary Fig. 2n–p), and across multiple cell lines (Supplementary Fig. 2q, r). The interaction profiles of 4C baits of different states were largely dominated by interacting regions of the same state as the baits. In addition, the *TMCO4* 4C data showed that most 4C interactions fell within the same MRR as the bait and only a handful of them were outside of the MRR. This suggested that MRR can have extensive internal looping.

We also carried out 4C experiments on the same bait across different cell lines. The interactions and the chromatin state at the bait locus varied in different cell lines, but the interaction profile maintained a preference for the same chromatin state as the bait (Supplementary Fig. 2q, r). As a further test of this concept, the extensive BB long-range interactions (green arcs) connecting *PSMD5* and *TOR1A* in K562 were validated using reciprocal 4C bait design. When the *PSMD5* bait region was A (active) in either GM12878 or HAP1 cells, the BB interactions were largely reduced and other types of interactions started to appear (Supplementary Fig. 2q).

Next, we analyzed the transcription factors binding to the regions of MRRs that are connected by chromatin interactions. ChIP-seq peaks of chromatin architectural proteins (CTCF, YY1, ZNF143), cohesin subunits (RAD21, SMC3), and transcription repression-associated proteins (EZH2, REST, GATAD2B) were downloaded from ENCODE and overlapped with the interacting regions of MRRs, which were then normalized to *Z* score and clustered by hierarchical clustering. Enrichments of one specific transcription factor can be found in several small clusters (Fig. 2g; Supplementary Data 3; YY1 in cluster_1, EZH2 in cluster_2, and SMC3 in cluster_3). Another cluster was identified with very high binding affinity of RAD21, REST, ZNF143, CTCF, and SMC3 (Fig. 2g cluster_5). Our results demonstrated that different chromatin architectural proteins are involved in the regulation of different silencer-associated chromatin interactions.

### CRISPR excision of a looping anchor within an MRR (MRR1-A1) leads to upregulation of multiple genes like *FGF18*, cell differentiation and tumor growth inhibition

**Fig. 3:**
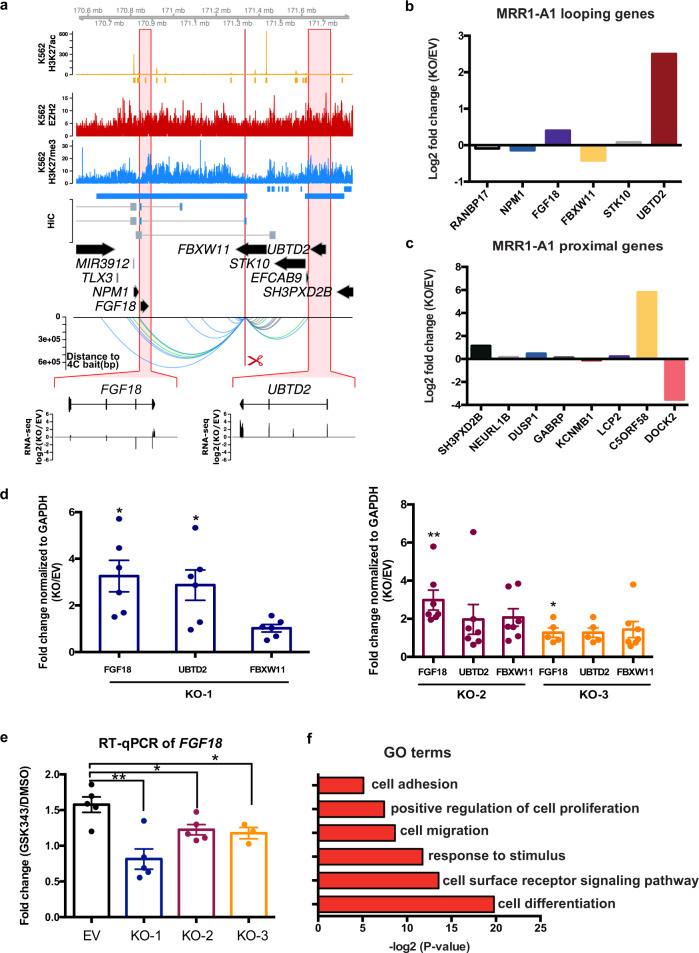
CRISPR excision of MRR1-A1 leads to gene upregulation of multiple proximal and looping genes including *FGF18*. **a** Screenshot showing EZH2 ChIP-seq, H3K27me3 ChIP-seq, H3K27ac ChIP-seq and chromatin interactions as identified from previously published Hi-C data^44^, gene information, and 4C performed on the CRISPR-excised region in wild-type cells confirming chromatin interactions to *FGF18*, as well as showing chromatin interactions to *UBTD2* and other genes. The regions highlighted in the red boxes are shown in more detail, with RNA-seq was shown as one CRISPR knockout clone over wild-type at *FGF18* and *UBTD2*. The blue bar shows the predicted whole MRR. The red box with the red scissors indicates the region which was excised. **b** RNA-seq fold changes calculated from two replicates of RNA-seq data of MRR1-A1 looping genes in one MRR1-A1 knockout clone (KO) as compared with one vector control clone (“Empty Vector”; “EV”)**. c** RNA-seq fold changes of MRR1-A1 proximal genes in KO as compared with EV. **d** RT-qPCR of *FGF18*, *UBTD2* and *FBXW11* in three different CRISPR-excised clones (“KO-1”, “KO-2”, “KO-3”) as compared with EV. *N* = 6 for each clone. **e** RT-qPCR of *FGF18* expression upon GSK343 treatment in EV and three KO clones. Fold change was plotted compared to *GAPDH* for EV and KO cells in DMSO and GSK343 condition. *N* = 5 for each clone. **f** Gene Ontology (GO) was performed using significant differentially expressed (DE) genes in the RNA-seq data which was shown as ‒log_2_(*p* value). All data shown here indicates average + standard error. *P* value is calculated by two-tailed student’s *t*-test. *P* value less than 0.05 is shown as *. *P* value less than 0.01 is shown as **.

Next, we asked if MRRs function as silencers to regulate gene expression. We selected 2 MRRs for functional testing based on the H3K27me3 signal, the presence of Hi-C anchors and the number of Hi-C anchors they associated with, as well as whether the target genes were involved in cell identity (Supplementary Note 1). Briefly, there are 974 MRRs in K562 (Supplementary Fig. 3a) and of those MRRs, 237 MRRs are associated with genes. Among these, 130 MRRs show proximal looping to genes (MRRs overlap with target gene promoters), 111 MRRs show distal looping to genes (MRRs loop over to the promoters of target genes by long-range chromatin interactions) and 51 MRRs show internal looping to genes (part of the MRR overlaps with the target gene promoter and the other part of the MRR loops over to the promoter of the target gene by long-range chromatin interactions). From this list, we selected MRR1, an internal looping example which showed 2 Hi-C loops to *FGF18*, a fibroblast growth factor involved in cell differentiation and cell-to-cell adhesion^46,47^ (Fig. 3a).

We designed the CRISPR deletion site at a 1 kb region in MRR1 (termed “MRR1-A1”) located in the *FBXW11* intronic region that was associated with one of two Hi-C anchors that loop over to *FGF18* (Fig. 3a). This region has high H3K27me3 as validated by ChIP-qPCR (Supplementary Fig. 3b). MRR1-A1 is part of cluster_8 (associated with low levels of cohesin proteins, high binding to GATAD2B; Supplementary Data 3) from Fig. 2g. We performed 4C using MRR1-A1 as the bait to detect all the genomic locations that have chromatin interactions with this region in wild-type K562. The 4C-seq results showed that this region indeed had chromatin interactions with *FGF18* and several other genes such as *NPM1* and *UBTD2* (Fig. 3a).

Next, we performed CRISPR deletion and generated three knock out (KO) clones (Supplementary Fig. 3c). To scan for target genes, we prepared RNA-seq from one KO clone and aligned this data with the 4C-seq data using MRR1-A1 as the bait (Fig. 3a). From RNA-seq fold changes of MRR1-A1 looping genes, we found upregulation of *FGF18* and *UBTD2* (Fig. 3b, Supplementary Fig. 3d). For proximal genes, we found upregulation of genes including *SH3PXD2B* and *C5ORF58* (Fig. 3c, Supplementary Fig. 3e). Among those genes, upregulation of the *FGF18* was further confirmed by RT-qPCR consistently in three different KO clones (Fig. 3d) while *UBTD2* was upregulated significantly in KO1 but not in other clones. Therefore, we focused on *FGF18* gene for further analysis.

Next, we treated the K562 cells with GSK343 (EZH2 methyltransferase inhibitor). Upon GSK343 treatment, *FGF18* gene was upregulated compared with DMSO control. This indicates that *FGF18* gene was upregulated upon H3K27me3 depletion. By contrast, in MRR1-A1 KO clones treated with GSK343, *FGF18* was upregulated to a much smaller extent as compared with wild-type cells (Fig. 3e). This indicates that *FGF18* gene upregulation upon H3K27me3 depletion is partially dependent on intact MRR1-A1, which further suggested that MRR1-A1 is a silencer.

To explore if the MRR1 is cell type-specific, we identified MRRs in seven cell lines and found that MRR1 is specific to two of the seven cell lines, K562 and GM12878 (Supplementary Fig. 4b) which suggested that silencers are specific to different cell types and might control the cell identity related-genes.

**Fig. 4:**
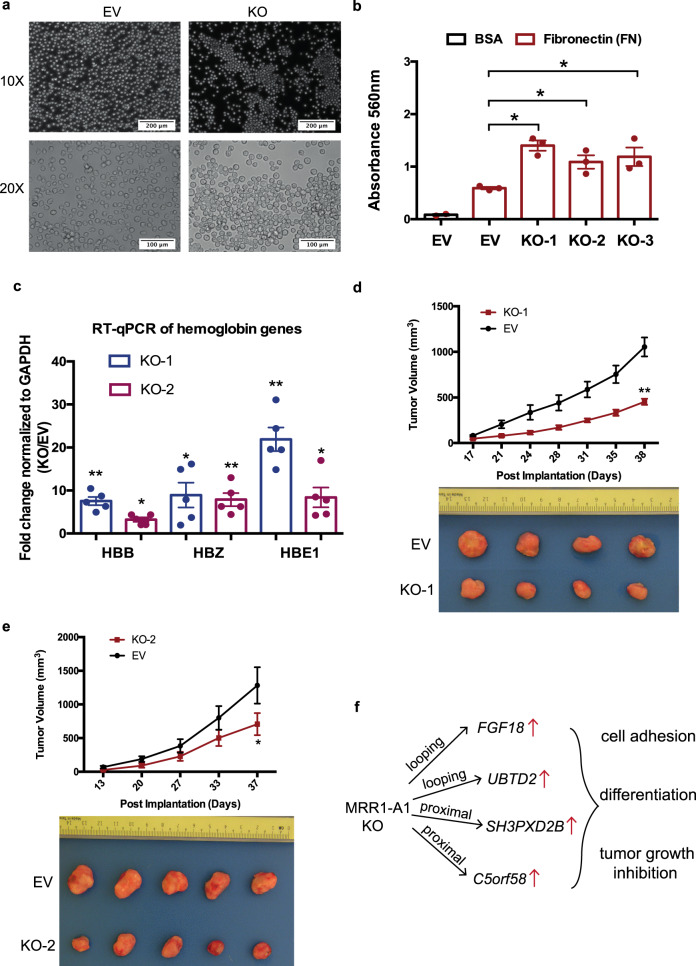
CRISPR excision of MRR1-A1 leads to altered adhesion, erythroid differentiation and tumor growth inhibition. **a** Light microscopy photos of empty vector (EV) and CRISPR knockout clones (KO) showing increased cell adhesion and aggregates in the KO clones. ×10 and ×20 magnification were shown. The results were repeated independently five times. **b** A fibronectin adhesion assay showed increased adhesion of the three CRISPR knockout clones (KO) as compared with empty vector (EV). Bovine Serum Albumin (BSA) was used as a negative control. *N* = 3 for each clone. **c**. RT-qPCR of haemoglobin genes (*HBB*, *HBZ* and *HBE1*) in EV and two KO clones. *N* = 5 for each clone. **d**, **e** Tumor growth in SCID (Severe Combined Immunodeficiency) mice injected with MRR1-A1 knock out clones and empty vector cells (EV). The upper panel shows the tumor growth curve, and data shown as tumor volume with different post implantation days. The panel below was the representative tumor picture at the final day. *N* = 5 for each group. **f** Schematic model: MRR1-A1 excision leads to changes in gene expression levels of multiple genes which further leads to cell adhesion, differentiation and tumor growth inhibition. All data shown here indicates average + standard error. *P* value is calculated by two-tailed student’s *t*-test. *P* value less than 0.05 is shown as *. *P* value less than 0.01 is shown as **.

Since *FGF18* has been reported to be involved in cell-to-cell adhesion^46,47^, we then asked if KO clones showed changes in adhesion. To address this, we performed gene ontology (GO) analysis which showed that KO clones may undergo cell adhesion and cell differentiation (Fig. 3f). First, we observed that the KO clones show increased adhesion to the cell culture plate surface and formed aggregates while wild type cells remained as suspension cells (Fig. 4a). The adhesion ability was further quantified by cell adhesion assays (Fig. 4b).

Next, because *FGF18* is associated with differentiation^46,47^, we investigated whether KO clones showed an erythroid differentiation phenotype. Cellular aggregates were reported by several publications^48,49^ to be associated with cell differentiation such as erythroid and megakaryocyte lineage of K562 cells. Therefore, we checked the expression of haemoglobin genes which can be the indicator of erythroid lineage differentiation^50^ in the RNA-seq data and further confirmed some of their upregulation (*HBB*, *HBZ* and *HBE1*) by RT-qPCR (Fig. 4c).

To investigate whether the differentiation phenotype might be partially caused by upregulation of *FGF18*, we performed siRNA knock down targeting *FGF18* gene in the KO clones, which led to 60–80% reduction in *FGF18* gene expression levels. Haemoglobin genes can be partially rescued by *FGF18* knocking down (Supplementary Fig. 4a) which suggested that erythroid differentiation was partially caused by *FGF18* upregulation (Fig. 4f). We speculate that *FGF18* siRNA knockdown did not lead to a complete rescue because MRR1-A1 knockout also upregulates other genes in addition to *FGF18*. For example, *SH3PXD2B* may also play roles in controlling erythroid differentiation^51^.

Leukemic cell differentiation induction is associated with cell growth inhibition and small molecule inhibitors such as All-*trans* Retinoic Acid (ATRA) that can induce differentiation have been useful in the treatment of Acute Promyelocytic Leukemia, suggesting that methods to induce differentiation could lead to potential leukemia treatments^50,52^. Therefore, we asked if silencer removal is associated with growth inhibition in vivo, given that silencer removal leads to cell differentiation. To test this, we performed xenograft experiments with two different KO clones. Both KO clones showed inhibition of tumor growth in the mice (Fig. 4d, e). This tumor growth inhibition suggested that MRR1-A1 might play tumor suppressor roles in leukemia and suggests the possibility that silencers can control cell identity through repression of tumor suppressor gene expression. In summary, our analyses suggested that MRR1-A1 can function as a looping silencer of *FGF18* as well as other genes. MRR1-A1 removal leads to cell identity changes such as cell adhesion, cell differentiation and tumor growth inhibition (Fig. 4f).

### CRISPR excision of a looping anchor within an MRR (MRR2-A1) leads to multiple gene upregulation including *IGF2*, cell differentiation and tumor growth inhibition

**Fig. 5:**
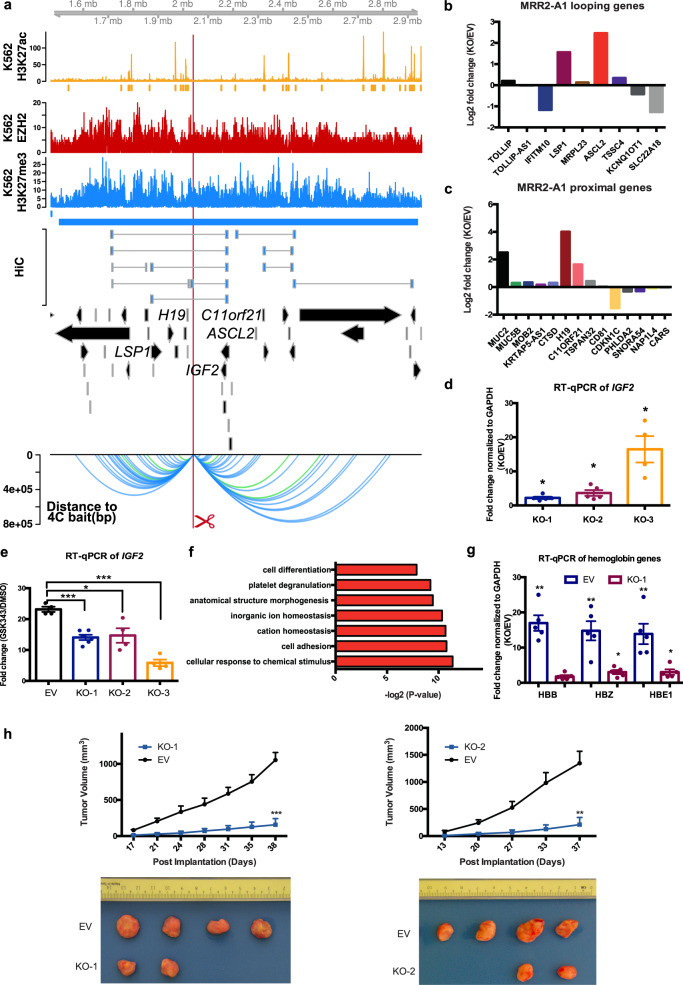
CRISPR excision of MRR2-A1 leads to multiple gene upregulation including *IGF2* gene, erythroid differentiation and tumor growth inhibition. **a** Screenshot showing EZH2 ChIP-seq, H3K27me3 ChIP-seq, H3K27ac ChIP-seq and chromatin interactions as identified from previously published Hi-C data^44^, gene information, and 4C performed on the CRISPR-excised region in wild-type cells confirming chromatin interactions to *IGF2* as well as other genes. The blue bar shows the predicted MRR. The red box with the red scissors indicates the region which was excised. **b** RNA-seq fold changes of MRR2-A1 looping genes in KO as compared with EV. **c** RNA-seq fold changes of MRR2-A1 proximal genes in KO as compared with EV. **d** RT-qPCR of *IGF2* in three different CRISPR-excised clones (KO-1, KO-2, KO-3) as compared with vector control cells (“EV”). *N* = 5 for each clone. **e** RT-qPCR of *IGF2* expression upon GSK343 treatment in EV and three KO clones. Fold change was plotted compared to *GAPDH* for EV and KO cells in DMSO and GSK343 condition. *N* = 5 for each clone. **f** Gene Ontology (GO) was performed using significant DE genes in the RNA-seq data shown as −log2(*p* value). **g** RT-qPCR of haemoglobin genes (*HBB*, *HBZ* and *HBE1*) in EV and two KO clones. *N* = 5 for each clone. **h** Tumor growth in SCID (Severe Combined Immunodeficiency) mice injected with MRR2-A1 knock out cells and empty vector cells (EV). The upper panel shows the tumor growth curve, and data shown as tumor volume with different post implantation days. The panel below was the representative tumor picture at the final day. *N* = 5 for each group. All data shown here indicates average + standard error. *P* value is calculated by two-tailed student’s *t*-test. *P* value less than 0.05 is shown as *. *P* value less than 0.01 is shown as **. *P* value less than 0.001 is shown as ***.

MRR2 was an internal looping example which showed 3 Hi-C loops to *IGF2*, an imprinted gene known to be associated with genomic silencers^53^ and involved in growth, development and cancer^54^ (Fig. 5a). MRR2 was characterized in the same manner as MRR1. Specifically, we designed another 1 kb deletion in MRR2 (termed “MRR2-A1”) located in an intergenic region that was associated with one of three Hi-C anchors looping over to *IGF2* (Fig. 5a). High H3K27me3 signal of MRR2-A1 was confirmed by ChIP-qPCR (Supplementary Fig. 5a) and chromatin interactions to *IGF2* and other genes were confirmed by 4C-seq (Fig. 5a). The MRR2-A1 anchor was in cluster_5 in Fig. 2G, and it has high binding affinity of CTCF, RAD21, SMC3 and REST (Supplementary Data 3).

RNA-seq of one MRR2-A1 KO clone (Supplementary Fig. 5b) showed upregulation of multiple genes which loop to MRR2-A1 (looping genes) including *LSP1*, *ASCL2* and *TSSC4* (Fig. 5b, Supplementary Fig. 5c). For proximal genes, *MUC2*, *H19* and *C11ORF21* were upregulated in KO (Fig. 5c, Supplementary Fig. 5d). H3K27me3 and H3K27ac ChIP-seq of this KO also showed changes in H3K27me3 and H3K27ac levels around MRR2 (Supplementary Fig. 5f). *IGF2* is expressed at a very low level in differentiated cells of the haematopoietic lineage^55^ and detected at very low levels by RNA-seq and therefore not shown in the fold change calculation. As *IGF2* has been previously shown to be regulated by silencers via chromatin interactions in mice^13^, we asked whether RT-qPCR could detect *IGF2* in our clones. Using RT-qPCR, we could detect *IGF2* and we found that *IGF2* was upregulated in all three KO clones (Fig. 5d). By contrast, *H19* was upregulated in one of the three KO clones as measured by RT-qPCR (Supplementary Fig. 5e). This indicated MRR2-A1 can function as a looping silencer to repress *IGF2* in human K562 cells. Again, *IGF2* was upregulated upon GSK343 treatment and the level of upregulation was reduced by MRR2-A1 removal, which showed that MRR2-A1 is a silencer (Fig. 5e). Similar to MRR1, MRR2 was also cell type-specific (Supplementary Fig. 5i).

Through gene ontology (GO) analysis of the RNA-seq on the MRR2-A1 KO clone, we found the term “cell differentiation” (Fig. 5f). Thus, we asked if these KO clones also undergo erythroid differentiation. RT-qPCR showed the haemoglobin genes (*HBB*, *HBZ* and *HE1*) were upregulated in the KO clones (Fig. 5g) and *IGF2* siRNA knock down can partially reduce this upregulation (Supplementary Fig. 5g) which suggests the differentiation was partially caused by *IGF2* upregulation in MRR2-A1 KO clones (Supplementary Fig. 5h). Similar to *FGF18* siRNA knockdown, we did not see a complete rescue of the differentiation phenotype by *IGF2* siRNA, which we speculate might be because MRR2-A1 upregulates other genes besides *IGF2*.

Finally, we tested to see whether the CRISPR KO clones showed tumor growth inhibition in vivo, similar to MRR1. Xenograft experiments showed severe tumor growth inhibition of two different clones (Fig. 5h) which further suggests that silencers can control cancer growth. Therefore, this MRR2-A1 example together with the MRR1-A1 example confirmed the existence of two looping silencers and showed that looping silencers are involved in the control of cell identity and tumor growth.

### *IGF2* looping silencer (MRR2-A1) removal caused changes of distant chromatin interactions

**Fig. 6:**
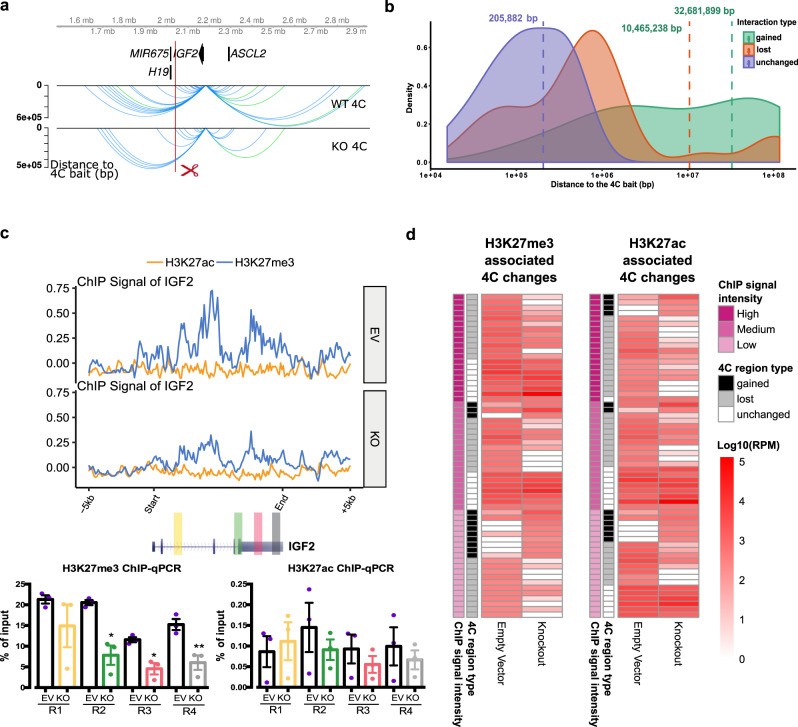
Initial histone states predict the changed loops upon MRR2-A1 removal. **a** Representative chromatin interactions at *IGF2* bait in KO and control clones which shown as loops. **b** The average distance of changed loops (gained loops and lost loops) is greater than unchanged loops upon MRR2-A1 KO when using *IGF2* promoter as the bait. **c** ChIP-seq and ChIP-qPCR of H3K27me3 and H3K27ac for four regions (R1-R4) at *IGF2* gene in EV and KO clones. *N* = 3 for each region. Data shown here are average + standard error. *P* value is calculated by two-tailed student’s *t*-test. *P* value less than 0.05 is shown as *. *P* value less than 0.01 is shown as **. **d** Heatmap about Integrative analysis of 4C, H3K27me3 and H3K27ac ChIP-seq in EV. Left panel: different 4C regions are classified according to their H3K27me3 signal intensity in EV. H3K27me3 signal level at these 4C regions are tertiled in three cohorts: high, medium, and low. 4C region type indicates different categories of 4C regions (Gained, lost and unchanged). The 4C interaction intensities are shown in log10 (RPM). Right panel: different 4C regions are classified according to their H3K27ac signal intensity in EV. Similar to the left panel, the H3K27ac signal level at these 4C regions are tertiled in three cohorts.

Through the previous two examples, we confirmed the existence of looping silencers and demonstrated they can control cell identity. Next, we investigated the epigenomic consequences of a looping silencer removal using the *IGF2* looping silencer (MRR2-A1) example. First, we asked whether the chromatin interaction landscape will be changed upon looping silencer removal. We performed 4C-seq in the KO and control clones. Using *IGF2* as the bait, we detected there are 33 chromatin interactions lost and 12 chromatin interactions gained after MRR2-A1 knocking out while a control bait remains highly unchanged (Fig. 6a, Supplementary Fig. 6a). Several lost loops were confirmed by 3C-PCR (Supplementary Fig. 6b) which indicates that looping silencer removal could lead to alterations in the chromatin interaction landscape.

Next, we classified chromatin interactions into three types: unchanged loops, gained loops and lost loops to explore their features. Through mapping their distance and density, we found that the average distances of changed loops are greater than unchanged loops which indicates that the long-range chromatin interactions which are further away from the bait tend to change (Fig. 6b). Moreover, the long-range chromatin interactions have a greater propensity to be lost than to be gained. Given that long-range chromatin interactions require more energy to be held together^56^, we speculated that when an anchor is lost, the amount of energy present in the system to hold together the long-range chromatin interactions may not be sufficient.

### Integrative analysis of histone modification states and chromatin interactions before and after *IGF2* looping silencer (MRR2-A1) removal

To understand the interplay between histone modification states and chromatin interactions, we performed H3K27me3 and H3K27ac ChIP-seq in the MRR2-A1 KO and control clones (Supplementary Fig. 6c). First, we found that H3K27me3 decreased along *IGF2* gene region upon knockout (Fig. 6c) while a control region remained similar (Supplementary Fig. 6d). This suggested that silencer removal will cause H3K27me3 loss at the target gene region.

Next, we performed integrative analyses of 4C-seq and ChIP-seq. Surprisingly, we found that the initial histone states of the cells before knockout were associated with whether the chromatin interactions would be gained, lost or unchanged upon knockout of MRR2-A1 (Fig. 6d). Specifically, very repressed loops with high H3K27me3 in control cells were unchanged or lost after KO. Loops with high H3K27ac and loops with low H3K27me3 in control cells tend to be easily changed either gained or lost after KO (Fig. 6d, Supplementary Data 4).

**Fig. 7:**
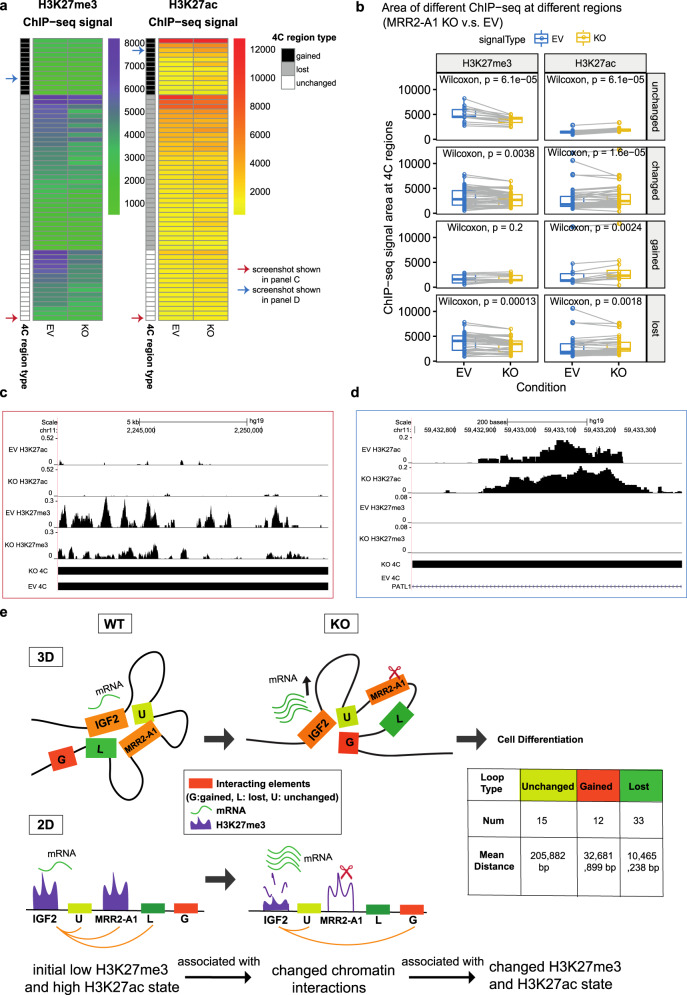
Unchanged loops and gained loops to *IGF2* become increased H3K27ac and decreased H3K27me3 levels upon MRR2-A1 removal. **a** Heatmap of ChIP-seq signal changes of H3K27me3 and H3K27ac at different types of 4C regions (gained, lost and unchanged) in empty vector (EV) and MRR2-A1 KO clones. Blue arrow: this region is shown as a screenshot in **c**. Red arrow: this region is shown as a screenshot in **d**. **b** Boxplots of ChIP-seq signal changes of H3K27me3 and H3K27ac at different types of 4C regions in EV and MRR2-A1 KO clones. The same 4C regions are connected by gray lines. Box and whiskers plot: whiskers were extended to the furthest value that is no more than 1.5 times the inter-quartile range. The boxes represent the 25th percentile, median, and 75th percentile. *p < = 0.05; **p < = 0.01; ***p < = 0.001; ****p < = 0.0001; ns, *p* > 0.05. **c** Zoomed screenshot about one of the unchanged 4C regions indicated in **a** which showed a decrease of H3K27me3. **d** Zoomed screenshot about one of the gained 4C regions in **a** which showed an increase of H3K27ac. **e** 3-dimensional and 2-dimensional cartoon schematics of our proposed model that initial histone states are associated with changed loops and MRR2-A1 removal leads to increase of H3K27ac levels on unchanged loops and gain of chromatin loops in regions with high H3K27ac levels.

Moreover, when we compared the integrative analysis in EV and KO, we observed a significant decrease in H3K27me3 for unchanged loops while levels H3K27ac increased slightly (Fig. 7a, b) which suggested that the repressive ability of the chromatin interaction became weaker and all the chromatin interactions looping to *IGF2* became more active in terms of histone state after MRR2-A1 KO. An example of the unchanged loops is shown in Fig. 7d, which displays unchanged loops to *IGF2* promoter along with decreased H3K27me3 levels in KO. When examining the gained loops to *IGF2* gene, we observed an increase in H3K27ac and no change in H3K27me3 levels (Fig. 7a, b) indicating that *IGF2* promoter could also gain more active loops. An example of the gained loops is shown in Fig. 7d.

Taken together, the regions that loop to *IGF2* in the KO clones are now more active with higher H3K27ac and lower H3K27me3 levels. These findings suggest two potential mechanisms by which I*GF2* might be upregulated in KO clones. First, *IGF2* showed a gain of chromatin loops to more active anchors and losses of loops to several repressive anchors. Second, the retained loops which had strong H3K27me3 levels at the control cells became weaker after KO (Fig. 7e). A combination of these mechanisms may operate in different cellular and physiological contexts.

### MRR-associated gene expression and long-range chromatin interactions are susceptible to EZH2 perturbation

In order to investigate the effects of H3K27me3 on MRR-associated chromatin interactions and target gene expression, we performed EZH2 inhibitor treatment (GSK343) in K562 cells to reduce H3K27me3 levels. Western blot confirmed that even just 1 μM of GSK343 treatment in K562 cells was sufficient to lead to a global loss of H3K27me3 (Supplementary Fig. 7a).

**Fig. 8:**
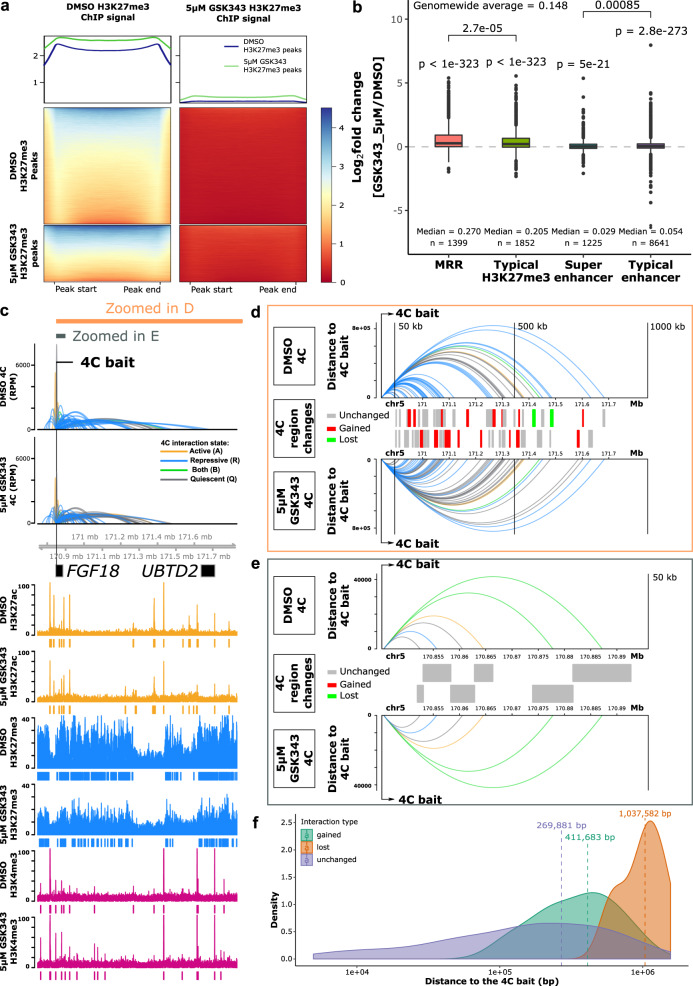
MRR-associated gene expression and chromatin interactions changes after EZH2 perturbation. **a** H3K27me3 ChIP-seq signal at peaks from DMSO-treated and 5 μM GSK343-treated K562 cells. Top panel: average H3K27me3 signal of H3K27me3 peaks in DMSO and GSK343 condition. Middle panel: H3K27me3 signal of DMSO H3K27me3 peaks in DMSO and GSK343 condition. Bottom panel: H3K27me3 signal of GSK343 H3K27me3 peaks in DMSO and GSK343 condition. **b** Expression changes of genes associated with different types of peaks in 5 μM GSK343-treated K562 cells. One-tailed wald test was used for testing significant upregulation. All the *P* values of genes in each category are aggregated using Lancaster aggregatin^90^. *p < = 0.05; **p < = 0.01; ***p < = 0.001; ****p < = 0.0001; ns, *p* > 0.05. Box and whiskers plot: whiskers were extended to the furthest value that is no more than 1.5 times the inter-quartile range. The boxes represent the 25th percentile, median, and 75th percentile. **c** 4C results of *FGF18* in DMSO and 5 μM GSK343-treated K562 cells. The colors of 4C interactions are based on the distal interacting regions. Blue: repressive; orange: active; green: both; gray: quiescent. The height of the 4C is shown in Reads Per Million (RPM). The ChIP-seq signal and peaks of H3K27ac, H3K27me3, and H3K4me3 are shown. **d** Zoomed-in view of 1000 kb region downstream of the 4C bait indicated in **c**. Top and bottom panel: 4C interactions in DMSO and 5 μM GSK343 conditions. Y-axis is scaled to the distance to the 4C bait. The color palette is the same as **c**. Middle panel: types of the 4C HindIII fragment. Gray, unchanged (present in both conditions); Red, gained (only present in 5 μM GSK343 condition); Green, lost (only present in DMSO condition). All the 4C regions are shown in two alternate rows to have a better visual separation. **e** Zoomed-in view of 50 kb region downstream of the 4C bait indicated in **c**. The details of each panel are the same as in **d**. **f** Density plot of different categories of 4C interactions on the same chromosome as the bait. All the 4C interactions that have *p* value < 0.05 on the same chromosome as the 4C bait are included.

H3K27me3 ChIP-Seq of DMSO-treated and 5 μM GSK343-treated K562 cells showed that the levels of H3K27me3 decreased globally, leading to the loss of nearly half of the H3K27me3 ChIP-seq peaks (Fig. 8a, Supplementary Data 5). However, there were still residual H3K27me3 peaks after GSK343 treatment, and these were the regions that had higher H3K27me3 signal before the treatment as compared with the susceptible peaks.

To interrogate the gene expression changes of MRR-related genes, we performed RNA-seq in DMSO-treated and 5 μM GSK343-treated K562 cells. We then investigate the expression of genes that: (1) overlapped with peaks at their transcription start sites (TSS); (2) associated with peaks via Hi–C interaction. The RNA-seq results indicated strong upregulation of H3K27me3-associated genes, while genes associated with H3K27ac peaks (super-enhancers or typical enhancers) underwent minimal net change (Fig. 8b, Supplementary Data 6). Notably, MRR-associated genes were the most strongly upregulated as compared with other categories (typical H3K27me3, super-enhancer and typical enhancers) (Fig. 8b). Similarly, a lower dose of 1 μM GSK343 treatment in K562 also induced H3K27me3 depletion and significant upregulation of MRR-associated genes as compared with other categories (Supplementary Fig. 7b). In addition, cell adhesion-related genes in RNA-seq of K562 cells were significantly upregulated (Supplementary Fig. 7c–e). This is in concordance with the increased cell adhesion in CRISPR KO clones (Fig. 4a, b), possibly due to depression of *FGF18* gene. Taken together, our results showed that MRR-associated genes were highly susceptible to EZH2 inhibition.

To further understand the chromatin interaction changes after EZH2 inhibition treatment, we also performed 4C and ChIP-seq experiments and investigated our candidate genes used in the CRISPR KO experiments in more detail. ChIP-seq data at *FGF18* gene showed that H3K27me3 level was decreased and there were accompanied lost peaks, while the H3K27ac and H3K4me3 signal were mostly unaltered (Fig. 8c). By comparing the 4C interactions at the *FGF18* promoter in DMSO and GSK343-treated conditions, we found that long-range 4C interactions were altered (Fig. 8d), while short-range 4C interactions were almost unchanged (Fig. 8e). A density plot showed that the unchanged 4C interactions have a closer distance relative to the 4C bait compared with gained or lost categories (Fig. 8f). We also performed 4C experiments in 5 μM treated GSK343 K562 cells using MRR1-A1, *IGF2*, and MRR2-A1 as baits, and their interaction profiles showed that short-range interactions are mostly unchanged (Supplementary Fig. 7f, g). To compare the effects of different drug concentrations, we performed all the 4C experiments using the same baits (*FGF18* promoter, MRR1-A1, *IGF2* promoter, and MRR2-A1) in 1 μM treated GSK343 K562 cells. The 4C interaction profiles in 5 μM and 1 μM treated GSK343 K562 cells were very similar (Supplementary Fig. 7h).

In addition, we performed 4C experiments using other baits in 1 μM treated GSK343 K526 cells which show the same conclusion that the short-range chromatin interactions in the vicinity of the 4C baits were largely unchanged (Supplementary Fig. 7i, j). By contrast, the long-range chromatin interactions tend to change.

Taken together, these results demonstrated that H3K27me3 perturbation by EZH2 inhibition, either genetically or pharmacologically, can lead to the alteration of long-range chromatin interactions.

### Integrative analysis of H3K27me3, H3K27ac and chromatin interactions upon EZH2 inhibition

**Fig. 9:**
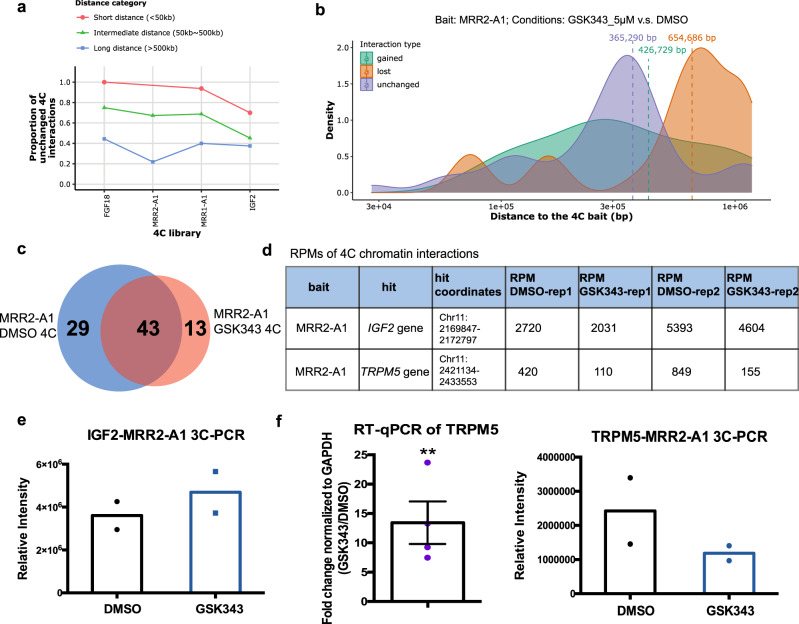
Analysis of stable and changing chromatin interactions upon EZH2 inhibition. **a** Proportions of unchanged 4C interactions in different distance categories (short, intermediate and long) in 5 μM GSK343-treated K562 cells. The bait name is used as the name of the 4C libraries. As the distance of 4C interactions increases, the proportion of unchanged 4C interactions drops, suggesting that long-range interactions are perturbed. **b** The average distance of changed loops (gained loops and lost loops) is greater than unchanged loops upon GSK343 treatment when using MRR2-A1 as the bait. **c** Venn diagram of 4C chromatin interactions using MRR2-A1 as the bait in DMSO and GSK343 condition. **d** Table of Reads Per Million (RPMs) of 4C chromatin interactions in two individual replicates. **e** 3C-PCR of *IGF2*-MRR2-A1 loop in DMSO and GSK343 condition by two independent 3C libraries. The data are shown as relative intensity. **f** RT-qPCR of *TRPM5* gene (*N* = 4) and 3C-PCR of *TRPM5*-MRR2-A1 in DMSO and GSK343 condition by two independent 3C libraries. All data shown here are average + standard error. *P* value is calculated by two-tailed student’s *t*-test. *P* value less than 0.01 is shown as **.

Since several examples including 4C-seq using *FGF18* promoter as the bait showed long-range chromatin interaction changes upon GSK343 treatment which is consistent with previous MRR2-A1 KO results, we wondered if all the 4C libraries showed the same trend. We classified the chromatin interactions into three categories (short distance, intermediate distance and long distance) based on their distance to the bait. We found that the short distance category has the highest proportion of unchanged loops in all the 4C libraries (Fig. 9a). A similar trend was also observed in 1 μM GSK343-treated K562 cells (Supplementary Fig. 8a). The results of all these libraries strengthen the conclusion that long-range chromatin interactions are susceptible to EZH2 inhibition. To note, although EZH2 inhibition and chromatin interaction anchor knockout are two very different types of perturbation experiments, both show that long-range chromatin interactions have a higher tendency to change upon perturbation as compared with short-range chromatin interactions.

Next, we examined the constant and dynamic chromatin interactions in relation to gene upregulation. We chose the MRR2-A1 region for our EZH2 inhibition analyses in order to compare our results with the MRR2-A1 KO results. 4C-seq data with MRR2-A1 as the bait showed 29 lost loops and 13 gained loops upon GSK343 treatment (Fig. 9c). We found the loop to *IGF2* remained unchanged (Fig. 9d, e, Supplementary Fig. 8c) while *IGF2* expression was increased (Fig. 5e). This phenomenon was also observed in MRR1-A1-*FGF18* loop (Supplementary Fig. 8b). Next, to investigate changing chromatin interactions, we selected *TRPM5* gene as an example from 29 lost loops (Fig. 9c). *TRPM5* gene was significantly upregulated upon GSK343 treatment (Fig. 9f). This upregulation was accompanied by disrupted looping to MRR2-A1, which was confirmed by 3C-PCR (Fig. 9d–f, Supplementary Fig. 8d). *TRPM5* gene promoter is more distal than *IGF2* gene promoter in terms of the distance to MRR2-A1 bait, which again supports the conclusion that long-range chromatin interactions tend to change upon EZH2 inhibition.

**Fig. 10:**
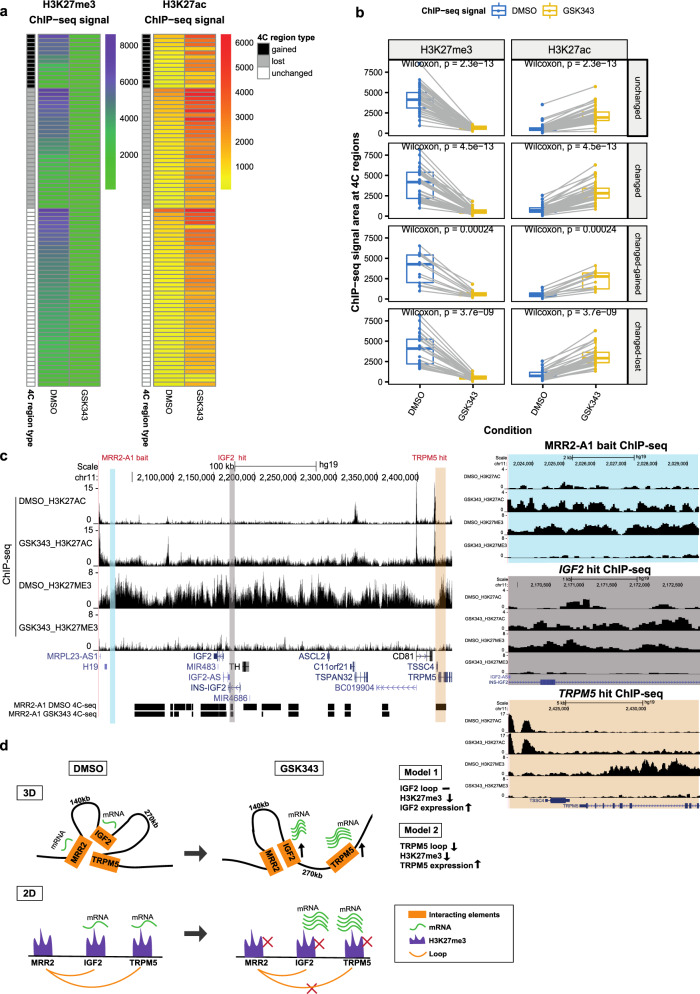
Integrative analysis of H3K27me3, H3K27ac and chromatin interactions upon EZH2 inhibition. **a** Heatmap of ChIP-seq signal changes of H3K27me3 and H3K27ac at different types of 4C regions (gained, lost and unchanged) in DMSO and GSK343 treated K562 cells. **b** Boxplots of ChIP-seq signal changes of H3K27me3 and H3K27ac at different types of 4C regions in DMSO and GSK343 treated K562 cells. The same 4C regions are connected by gray lines. Unchanged (*n* = 86), changed-gained (*n* = 26), changed-lost (*n* = 58), changed (gained plus lost, *n* = 84). Wilcoxon paired test *p* values are indicated. Box and whiskers plot: whiskers were extended to the furthest value that is no more than 1.5 times the inter-quartile range. The boxes represent the 25th percentile, median, and 75th percentile. **c** Screenshot of H3K27me3 and H3K27ac ChIP-seq at MRR2-A1, *IGF2* gene and *TRPM5* gene regions in DMSO and GSK343 as well as 4C-seq using MRR2-A1 as the bait. MRR2-A1 bait, *IGF2* bait and *TRPM5* bait were highlighted and zoomed in for ChIP-seq. **d** 3-dimensional and 2-dimensional cartoon schematics of our proposed model involving two mechanisms of how GSK343 leads to *IGF2* gene and *TRPM5* gene upregulation at stable and changing chromatin interactions respectively.

As the MRR2-A1 KO example demonstrated that initial histone state is associated with chromatin interactions and silencer KO leads to altered chromatin interactions and histone state which demonstrates the interplay between histone modifications and chromatin interactions (Fig. 7e), we asked whether EZH2 inhibition by GSK343 will also lead to histone modifications alterations at changing and unchanging chromatin interactions. We performed integrative analysis using MRR2-A1 4C-seq and ChIP-seq as we did for the KO clones (Fig. 10a, b). Unlike the integrative analysis of MRR2-A1 KO which showed that the initial histone state could predict which chromatin interactions would change (Fig. 6d), the histone states of the DMSO condition could not predict which chromatin interactions would change (Supplementary Fig. 9a).

Upon GSK343 treatment, we observed there are global histone modification changes. H3K27me3 levels decreased and H3K27ac levels increased for all three categories: unchanged, gained and lost loops (Fig. 10a, b), which is consistent with the western blot results showing global loss of H3K27me3 (Supplementary Fig. 7a). In terms of the two upregulated genes (*IGF2* and *TRPM5*) that we explored before, they both demonstrated loss of H3K27me3 and gain of H3K27ac at the 4C interacting anchors (Fig. 10c) although the loop to *IGF2* remained unchanged while the loop to *TRPM5* was reduced. This trend of decreased H3K27me3 and increased H3K27ac was also observed in *FGF18* which showed stable looping in parallel with increased gene expression upon GSK343 treatment (Supplementary Fig. 9b).

Taken together, the integrative analysis combined with the 3C-PCR showed two models regarding how EZH2 inhibition functions (Fig. 10d). Model 1 showed decreased H3K27me3 levels with stable loops upon GSK343 treatment which applies to both *IGF2* gene and *FGF18* gene. However, we noticed differences between 4C-seq using the *IGF2* promoter as the bait and 4C-seq using the *FGF18* promoter as the bait (Supplementary Fig. 9c, d). Specifically, there are many repressive loops lost at the *IGF2* promoter while there are only a few repressive loops lost at the *FGF18* promoter in GSK343 condition which may explain the differences in gene upregulation upon GSK343 treatment (Figs. 3e and 5e). Model 2 showed decreased H3K27me3 levels with disrupted loop upon GSK343 treatment as observed with the *TRPM5* gene (Fig. 10d). Therefore, in terms of the relationship between H3K27me3, chromatin interactions and gene upregulation, we think that there are two different models. The first model is that long-range chromatin interactions facilitate the deposition of H3K27me3 modification onto the target gene promoter by MRRs to repress target genes (e.g. *IGF2* and *FGF18*). The second model is that depletion of H3K27me3 abrogates long-range chromatin interactions and upregulates target genes (e.g., *TRPM5*). H3K27me3 might facilitate the MRR2-TRPM5 chromatin interaction, which in turn silences *TRPM5*; alternatively, H3K27me3 might facilitate the MRR2-TRPM5 chromatin interaction and *TRPM5* silencing in parallel.

## Discussion

Silencers are important regulatory elements for gene regulation, and several studies have suggested that they loop to target genes, in a manner analogous to enhancers. Although there are several examples of proposed silencers that have been experimentally validated (Supplementary Table 1) and several methods have been proposed to identify silencer elements (Supplementary Table 2), there is however no consensus on their foolproof identity yet. In addition, while looping silencers have been shown to exist in *Drosophila*^12^ and in mice^13^, no silencers that work via a looping mechanism have been characterized in humans yet. Here, we propose a method to identify H3K27me3-rich regions (MRRs) or putative “super-silencers” through clustering and ranking H3K27me3 signals.

We found that MRRs are highly associated with chromatin interactions and can be perturbed by EZH2 inhibition. Through H3K27me3 clustering, ranking and associate them with chromatin interactions, we validated two looping silencer examples (MRR1-A1 and MRR2-A1). We showed that silencer removal causes cell identity changes and further related to tumor growth inhibition. Moreover, MRR2-A1 example demonstrated that silencer removal will cause changes of long-range chromatin interactions and high H3K27ac loops were gained to activate *IGF2* gene expression.

The mechanism of how silencers function to repress genes will be an important topic to explore. Through the *IGF2* silencer example, we showed that that looping silencer removal causes distant loops to change and histone states in the initial conditions can predict loop changes. Importantly, we found that loops with high H3K27ac and low H3K27me3 tend to change, which provides evidence that histone modifications can affect overall genome architecture. Secondly, we found that short-range loops tend to remain unchanged while long range loops are disturbed either upon silencer KO or GSK343 treatment which is in line with the finding that showed PRC1 and PRC2 are necessary to maintain the chromatin interactions landscape^17,57^. Thirdly, there are multiple regions inside an MRR that are involved in chromatin interactions and may also function as silencers. It would be informative to see whether the putative silencers in an MRR function similarly or differently and to dissect different functional mechanisms of silencers. Fourthly, transcription factors can contribute to the chromatin interaction landscape and cell type-specific transcription factors may result in different chromatin interaction landscape^58^. Therefore, elucidating the transcription factors involved in silencer functioning would be an important future direction for research.

Another question concerns the relative importance of H3K27me3 at distal silencers as compared with target gene promoters in terms of controlling gene expression levels. In our CRISPR knockout results, we observed that excision of a distally interacting MRR region (MRR2-A1) to *IGF2* can lead to *IGF2* upregulation, which indicated the repressive ability of H3K27me3 at distal MRRs. This question could be further addressed by more targeted perturbation of H3K27me3 at both or either end of the chromatin interactions and investigating how gene expression levels change.

Our EZH2 inhibition treatment showed that MRR-associated genes as well as long-range chromatin interactions were susceptible to the depletion of H3K27me3 histone marks. MRR-associated genes were more susceptible to the depletion of H3K27me3 marks than genes associated with typical H3K27me3 peaks. This result suggested that the response of different genes to H3K27me3 loss may correlate with their chromatin state. Although differences in chromatin interactions have been observed in cells at different developmental stages^59,60^, whether chromatin interactions can be affected by the perturbation of histone modifications is still an open question.

Next, we and other people found that silencers are cell-type specific and highly context-dependent^27–29^ (Supplementary Table 2). Specifically, the same genomic region was a silencer in one cell line but a super-enhancer in another cell line. Not surprisingly, this change is associated with different gene expression in the different cell lines. Moreover, it has been shown that silencers can transit into active enhancers during differentiation^13^. Thus, the study of relationships between cell types and silencers can shed light on cell type-specific regulation of gene expression. The mechanism by which important oncogenes such as *TERT* are silenced in normal cells is unclear. It would be important to investigate whether *TERT* is regulated by MRRs that transit into active enhancers in cancer cells.

We found that silencer removal leads to cell differentiation and tumor growth inhibition, which is in line with previous observed studies that showed that gain of function mutations in EZH2 led to increased levels of H3K27me3 at TADs and repression of tumor suppressor genes^57^. It will be relevant to study the detailed mechanism of how silencers regulate tumor suppressor genes. In this way, it may be possible for us to activate tumor suppressor gene expression by perturbing silencers, just as super-enhancer perturbation can result in loss of oncogene expression^32^.

Notably, the question of whether super-enhancers are indeed different from enhancers is not settled yet^61^. Our research raises similar questions: are MRRs, which are potential “super-silencers”, different from typical silencers? The regions of the long MRR that are critical for silencer function are not fully elucidated yet. Here we showed that the components of the MRRs that are involved in looping interactions are important in repressing long-range chromatin interactions, while the roles of other components of the MRRs are not yet known. Moreover, we found that different anchors within the same MRR can be associated with different proteins, suggesting that these different anchors may play different roles within the MRR. Detailed dissection of the different anchors and other components of MRRs will be required to answer these questions in future work.

Moreover, it will be informative to explore how looping is mediated at MRRs. Given that super-enhancers have been shown to be involved in phase condensation^62^, and HP1 which is a component of constitutive heterochromatin associated with H3K9me3 has also been shown to be able to form phase condensates^63^, it would be important to explore whether the PRC2 complex and H3K27me3 can give rise to phase condensates that result in the formation of “super-silencers”.

In conclusion, maintenance of cellular identity requires that the right genes are expressed and other genes are silenced. Distal looping silencers have been well explored in *Drosophila* and mice^64^ but there are no known examples in human. Our results add to the understanding of silencers by identifying silencer elements in human and demonstrating the existence of looping silencers in human. Just as the concept of “super-enhancers” has been useful in identifying oncogenes and therapeutic vulnerabilities in cancer cells, the concept of silencers calling by clustering of H3K27me3 may be useful in identifying genes involved in controlling cellular identity and cancer progression.

## Methods

We performed Hi-C interaction analysis, ChIP-seq, RNA-seq, gene expression analyses, cell culture, RT-qPCR, CRISPR excision, 4C, 3C, xenograft models, western blot, adhesion assays, and growth curves as described in the following sections. A list of all libraries used and generated is provided in Supplementary Data 7. A list of all the primers used is provided in Supplementary Table 3.

### Definition of H3K27me3-rich regions (MRRs)

H3K27me3 ChIP-seq signal and peaks were obtained from ENCODE and used as inputs of an in-house customized script that mimicked the signal calculation of the ROSE (0.1) package. Missing H3K27me3 peaks from ENCODE were called using MACS2 (2.1.0.20150731)^65^ with pooled replicates using option “–broad −q 0.05”. First, ChIP-seq peaks of H3K27me3 were stitched using a window size of 4 kb. After stitching, the treatment and control ChIP-seq signal of the stitched peaks were calculated and used to rank all the stitched peaks. The rank-ordered signal with a slope of 1 was used as the cut-off for defining H3K27me3-rich regions (MRRs). Super-enhancers were called in a similar manner except that a stitching window of 12.5 kb was used and H3K27ac ChIP-seq signal was used in the ranking process.

### Cell culture

GM12878 normal lymphoblastoid cell line and Chronic Myelogenous Leukemia cell line K562 were cultured in RPMI-1640 supplemented with 10% Fetal Bovine Serum (FBS) and 1% penicillin-streptomycin. HAP1 cell line is a haploid human cell line that was derived from KBM7 cells^66^. HAP1 wild-type cells (purchased from Horizon) were cultured in IMDM supplemented with 10% FBS and 1% penicillin-streptomycin. All cultures were maintained at 37 °C, 5% CO_2_ in a humidified incubator.

### Circular chromosome conformation capture (4C)

4C-seq assays were performed according to Splinter et al.^67^ with slight modifications. Briefly, 4 × 10^7^ cells were cross-linked with 1% formaldehyde. The nuclei pellets were isolated by cell lysis with cold lysis buffer (10 mM Tris-HCl, 10 mM NaCl, 5 mM EDTA, 0.5% NP 40) supplemented with protease inhibitors (Roche). First step digestion was performed overnight at 37 °C with HindIII enzyme (NEB). Digestion efficiency was measured by RT-qPCR with HindIII site-specific primers. After confirmation of good digestion efficiency, DNA was ligated overnight at 16 °C by T4 DNA ligase (Thermo Scientific) and de-crosslinked. Following de-crosslinking, DNA was extracted by phenol-chloroform and this is the 3C library. The DNA was then processed for second digestion with DpnII enzyme (NEB) overnight at 37 °C. After final ligation, 4C template DNA was obtained, and the concentration was determined using Qubit assays (Thermo Scientific). The 4C template DNA was then amplified using specific primers with Illumina Nextera adapters and sent for sequencing on the MiSeq system. All the 4C genome coordinates are listed in Supplementary Table 4.

### 3C-PCR

The 3C libraries were generated as described in the 4C section. Digestion efficiency was checked by gel electrophoresis and concentration was determined by Qubit assay. The Taq PCR core kit (Qiagen) was used for PCR reactions with 600 ng 3C library template using the following protocol: 98 °C 3 min, 33 cycles [94 °C 1 min, 60 °C 1 min, 72 °C 20 sec], 72 °C 10 min. PCR products were run on 1.5% agarose gels. After gel electrophoresis, bands corresponding to the expected products were gel excised (Qiagen) and purified for Sanger sequencing. The intensities of the bands were measured by Image Lab. Primers were designed for 3C-PCR following the unidirectional strategy^68^. Primers used are listed in Supplementary Table 3. At least two replicates were performed for 3C analyses.

### ChIP-seq and ChIP-qPCR

ChIP-seq was performed according to Robertson et al.^69^ with slight modifications. Briefly, cells were crosslinked with 1% methanol-free formaldehyde (Thermo Scientific) at room temperature for 10 min, followed by quenching with glycine for 5 min at room temperature. The fixed cell pellet was lysed in 1% SDS lysis buffer supplemented with protease inhibitor cocktail tablet (Roche), and sonicated using Bioruptor (Diagenode).

The cell lysate was precleared through centrifugation in dilution buffer and incubation with Protein G Dynabeads (Invitrogen) overnight at 4 °C. The precleared lysate was added into the prepared antibody-conjugated beads, and incubated overnight at 4 °C, with rotation. The beads were then washed thrice in 0.1% SDS lysis buffer, once in high salt wash buffer, once in lithium chloride wash and once in TE buffer. The beads were eluted in elution buffer treated with RNase A (Qiagen) followed by decrosslinking with Proteinase K (Ambion) at 37 °C overnight. ChIP DNA was cleaned up with QIAquick PCR purification kit (Qiagen), and quantitated using Qubit High Sensitivity dsDNA Assay (Invitrogen).

ChIP DNA was used for the construction of DNA library for Illumina HiSeq 4000 NGS sequencing using ThruPLEX DNA-seq 48D Kit (Rubicon) according to the instruction. Antibodies used include H3K4me3 (#ab8580, Abcam), H3K27me3 (C36B11, Cell Signaling Technologies), H3K27ac (#ab4729, Abcam) and mouse IgG (#sc-2025, Santa Cruz). 3.5 μg of antibodies were used for each ChIP.

ChIP-qPCR reactions were performed in triplicates on a Quantstudio 5 quantitative PCR machine (Life Technologies) using GoTaq qPCR Master Mix (Promega). Primers used are listed in Supplementary Table 3.

### EZH2 inhibitor treatment

Small molecular inhibitor GSK343 (Sigma-Aldrich) targeting EZH2 were solubilized in DMSO (Sigma-Aldrich) according to the manufacturer’s instructions and used at a final concentration of 5 μM. K562 cells with GSK343 or DMSO vehicle control were incubated at 37 °C, 5% CO_2_ humidified incubator for 48 h before harvesting for various experiments.

### RNA extraction and RT-qPCR

Total RNA were isolated from the cells using RNeasy Mini Kit (Qiagen) with on-column DNase digestion (Qiagen). 1 ug of total RNA was then reverse transcribed to cDNA using the SuperScript III first-strand synthesis system using oligodT (Invitrogen). The expression levels of various genes were analysed by real-time PCR. Quantitative real-time PCR (qPCR) was performed using the Applied Biosystems QuantStudio 3 Real-Time PCR system using SYBR Green PCR Master Mix and appropriate primers. Primers used are listed in Supplementary Table 3 and tested by plotting the standard curve for various dilution ratios. We only accepted the primers whose efficiency were between 80%–120%. The transcript levels of genes were analysed by 2^−ΔΔCt^ method^70^.

### RNA-seq

Total RNA was extracted from cells using RNeasy Mini Kit (Qiagen), with on-column DNase I treatment (Qiagen). The quality of the RNA extracted was checked using Agilent RNA 6000 Nano Kit (Agilent), and quantitated using Nanodrop ND1000 (Thermo Scientific). 850 ng of RNA was used for the construction of cDNA library for Illumina HiSeq 2500 High Output v4 NGS sequencing using TruSeq Stranded Total RNA LT (w/ Ribo- Zero Gold) Set A (Illumina) as per protocol.

### Protein extraction and western blot

Proteins were extracted from the cells using RIPA buffer (Sigma-Aldrich) with protease inhibitor cocktail (Life Technologies). Protein concentrations were determined using BCA assay (Thermo Scientific). 20 μg of proteins were separated in 4–20% Mini-PROTEAN^®^ TGX™ Precast Gels (Bio-Rad) and transferred to PVDF membrane. After blocking with TBST containing 5% nonfat dried milk for 1 h at room temperature, the membrane was washed twice with TBST and incubated with primary antibodies: EZH2 (Cell Signaling Technology AC22), beta-Actin (abcam ab6276), total H3 (abcam ab1791) and H3K27me3 (Cell Signaling Technology C36B11) overnight at 4 °C. Primary antibodies are diluted at 1:1000 with 5% non-fat dried milk (dissolved in TBST). The membrane was washed three times with TBST for 10 min and then incubated for 1 h at room temperature with either mouse (CST 7076) or rabbit (CST 7074) HRP-conjugated secondary antibodies (Cell Signaling Technology) diluted at 1:5000 or 1:2000 with 5% non-fat dried milk (dissolved in TBST), respectively. After extensive washing, bands were detected by enhanced chemiluminescence reagent (Bio-Rad) and imaged using the ChemiDoc™ imaging system (Bio-Rad) or ImageQuant™ LAS 500 (GE healthcare).

### CRISPR excision

CRISPR excision was performed using all-in-one CRISPR/Cas9 vector system^71^. Briefly, gRNAs were designed using Zhang Feng’s website (http:// CRISPR.mit.edu)^72^ and two gRNAs were designed for each region. Single gRNA was cloned into either pX330A/pX330S vector (gift from Li Shang, pX330A modified to include GFP reporter marker) followed by Sanger sequencing to confirm insertion of single gRNA. Golden gate assembly of two gRNAs was performed according to Sakuma et al.^71^. Positive two gRNAs insertion plasmids were confirmed by Sanger sequencing using CRISPR-step2-F and CRISPR-step2-R primers (sequences were shown in Supplementary Table 3). The plasmid was then electroporated into the K562 cell line using the Neon transfection system (Thermo Fisher). After 48 h, transfected cells were FACS sorted into 96-well plates as single-cell colonies based on GFP signal.

Cells were harvested from each clone, pelleted and lysed in lysis buffer. Genotyping was carried out using an internal and a flanking primer pair. Final PCR products were imaged by agarose gel electrophoresis, and successful clones were confirmed through Sanger sequencing (First Base).

### Adhesion assay

Cell adhesion assay was performed using CytoSelect 48-Well Adhesion Assay (Cell Biolabs, San Diego, CA) according to the manufacturer’s protocol. Briefly, a cell suspension (5 × 10^5^ cells/200 ml FBS-free medium) was added to fibronectin-coated wells and BSA-coated wells (negative control) respectively. After 3 h of incubation, cells were washed with PBS, stained with crystal violet and then eluted with extraction solution. The levels of adhesion were quantified by optical absorbance at 560 nm using the Tecan plate reader.

### Growth curve assay

1000 cells/well were seeded in 96 well plates and cell growth was measured at day 0, day 1, day 2, day 3, day 4 and day 5 using the CellTiterGlo assay kit (Promega, G7571). Luminescence was measured on a Tecan plate reader.

### SiRNA knock down experiment

siRNAs (Thermo Fisher) were introduced into cells using Neo transfection system (Thermo Fisher) according to the manufacturer’s instructions. *FGF18*-siRNA-1, 5′-GAGACGGAAUUCUACCUGUtt-3′, *FGF18*-siRNA-2, 5′-AGACACCUUCGGUAGUCAAtt-3′, *IGF2*-siRNA-1, 5′-CCAUGCAAAUGAAAUGUAAtt-3′ *IGF2*-siRNA-2, 5′-GGAAGCACAGCAGCAUCUUtt-3′ were used for RNA interference. After 48 h incubation, cells were harvested for RT-qPCR. Primers used were listed in Supplementary Table 3.

### Xenograft experiment

All animal experiments were carried out in accordance with ethical guidelines and approved by the Institutional Animal Care and Use Committee (IACUC), Biological Resource Center (BRC) A*STAR under protocol ID #161111.

Mice were purchased from InVivos, Singapore and fed with standard laboratory diet and distilled water ad libitum. The animals were kept on a 12 h light/dark cycle at 22 ± 2 °C in individually ventilated caging system with 50–65% humidity in the Biological Resource Centre, A-Star, Singapore.

Female CB17 SCID mice (6–8 weeks old) were used for the present study. The mice (*n* = 5) were injected subcutaneously. All the mice were monitored for tumor growth at the site of inoculation and the tumor volume was measured twice a week using Vernier caliper for 40 days or till the tumor volume reaches 1000 mm^3^ whichever is earlier. The tumor volume was calculated using the following formula V = a × b2 × 0.52, where a is the largest and b the smallest diameter of the tumor. At the end of the experimental period, the tumors were resected out and each tumor piece was then divided into 2 pieces. One piece of the tumor was fixed in 10% NBF for 24 h at room temperature and then paraffin embedded for H&E & IHC analysis. The other piece was snap frozen for RNA and protein analysis.

KO-1 and EV were injected into separate mice in Experiment 1. In view of the concern that the differences in tumor growth seen in Experiment 1 might be because the KO-1 and EV were grown in different mice, in experiment 2, KO-2 and EV were injected in the same mice (left side and right side).

### Randomization process to overlap ReSE list

MRR and typical H3K27me3 peaks were first randomly shuffled to another position on the same chromosome for 1000 times. After that, the resulting genomic regions of the 1000 times shuffling were overlapped with ReSE^29^ list and calculate the overlap percentage. (1) First randomly shuffled 1000 times on MRR/typical H3K27me3 peaks on the same chromosome; (2) calculated overlap percentage using the 1000 time randomly shuffled regions.

### Expression changes associated with state changes in different cell lines

Genes were classified based on the states of their overlapping peaks in different cell lines: [state in the first cell line] vs. [state in the second cell line], where the state can be super-enhancer (SE), H3K27me3-rich region (MRR), typical enhancer (H3K27ac), typical H3K27me3 peak (H3K27me3), or no overlapping peaks (Others). The expression data is from Epigenetic RoadMap68 and in-house HAP1 WT RNA-seq.

### Definition of different categories of Hi–C interactions

Hi-C anchors are classified by whether they overlap with H3K27me3 or H3K27ac peaks. A (active) overlap with only H3K27ac peaks; R (repressive), overlap with only H3K27me3 peaks; Q (quiescent), overlap with neither H3K27ac nor H3K27me3 peaks; B (both), overlap with both H3K27ac and H3K27me3 peaks. The height of Hi–C interactions (arcs) represents the highest read counts in the interacting regions.

### RNA-seq, ChIP-seq and 4C data analysis

For reads of RNA-seq and ChIP-seq, adaptors are trimmed off by trimmomatic (0.38)^73^ with option “TruSeq3-PE.fa:2:30:10 LEADING:3 TRAILING:3 SLIDINGWINDOW:4:15 MINLEN:36” and retained only those properly-paired reads after trimming. RNA-seq reads were analyzed with kallisto (0.44.0)^74^ with option “-b 100”. Differentially expressed genes were called using sleuth (0.29.0)^75^ with gene-level aggregation and wald test. ChIP-seq reads of H3K2me3 and H3K27ac were mapped by BOWTIE2 (v2.2.5)^76^ using default parameters in pair-end mode and filter out alignment with a mapq score smaller than 30. The two replicates were combined and peaks and bigWig files were generated by MACS2 (2.1.0.20150731)^65^ using option “-q 0.01” for H3K27ac and H3K4me3 and “–broad –broad-cutoff 0.1 -q 0.05” "for H3K27me3. 4C reads were trimmed off HindIII digestion site using tagdust (2.33)^77^ and only those remained paired were mapped by BOWTIE2 (v2.2.5)^76^ with option “–end-to-end” in single-end mode. R3Cseq (1.24.0)^78^ was used to call significant interactions against Hind III digested genome background with a cut-off *p* value of 0.05. Two replicates were performed for each 4C analysis, and significant interactions from the two replicates were pooled.

### Expression of genes involved in proximal, distal, and internal chromatin interactions

Genes were first associated with Hi–C interactions. Proximal/distal/internal labels were assigned according to the positional relationship between the gene and MRRs as described in Fig. 2c. The control category is generated by (1) first filtering out genes that are overlapped with ENCODE blacklist regions and also H3K9me3 peaks as H3K9me3 is associated with constitutive heterochromatin and such regions are likely to be highly silenced; (2) only retaining genes that overlapped with Hi–C interactions; (3) randomly sampling the same amount of genes as the average gene number in proximal/distal/internal categories.

### Visualization of chromatin interactions, and ChIP-seq data

Hi-C and 4C interactions were drawn in arc style using Sushi (1.16.0)^79^ from Bioconductor. The heights of Hi-C and 4C were the largest read counts at Hi-C interacting regions and RPM at 4C interacting regions, respectively. The colors of Hi-C and 4C interactions were decided by the state on the distal interacting regions relative to gene TSS or 4C baits, respectively. Blue: repressive; orange: active; green: both; gray: quiescent. Tracks of ChIP-seq signal and peaks were generated by Gviz (1.22.3)^80^ from Bioconductor.

### Feature enrichment, gene ontology and pathway enrichment analysis

Genomic feature enrichment analysis was performed using R package annotatr (1.8.0)^81^. Gene ontology, pathway enrichment analysis (REACTOME & KEGG) and map-view representation of enriched pathways were performed using R package clusterProfiler (3.10.1)^82^ and ReactomeRA (1.26.0)^83^.

### Gene expression specificity

Cell line expression data were obtained from Epigenetic RoadMap (K562, GM12878, H1hESC, HeLaS3 and HepG2)^84^, CCLE (KARPAS, Pfeiffer, and WSUDLCL2)^85^. Gene expressions in each cell line were compared with 69 facets of annotated CAGE clusters with normalized expression data from FANTOM5^86^. The grouping facets were obtained from Andersson et al.^87^. The average expression of all samples in a facet was assigned to that facet. For each cell line, gene expression was considered as 1 facet and combined with the other 69 facets from FANTOM5 to form 70 facets in total. Gene expression specificity was calculated on these 70 facets for each cell line independently. The specificity of each gene X is calculated by: Specificity(X) = 1 − (entropy(X)/log2(N)) where X is the vector of expression values of the cluster in across all facets, and *N* = | × | . The definition of specificity is identical to that used by Andersson et al.^87^. Quartile Q1 and Q3 are used as cut-off of “Low specificity” and “High specificity”, respectively.

### Transcription factor binding enrichment analysis

ChIP-seq peaks of CTCF (“ENCFF738TKN”), RAD21 (“ENCFF002CXU”), SMC3 (“ENCFF041YQC”), REST (“ENCFF895QLA”), ZNF143 (“ENCFF114IWY”), EZH2 (“ENCFF083IDB”), GATAD2B (“ENCFF549KOD”), and YY1 (“ENCFF557DSM”) from K562 were downloaded from ENCODE. Interacting regions of MRRs were generated by overlapping MRRs with Hi-C loops. The number of overlapping ChIP-seq peaks was calculated by overlapping interacting regions with different TF. These numbers were then standardized into *Z* score across all interacting regions and subjected to heatmap clustering using pheatmap (1.0.12) (https://CRAN.R-project.org/package=pheatmap)^88^.

### Analysis of changing as compared with unchanging chromatin interactions

All the 4C interactions that passed the threshold of *p* value < 0.05 in each 4C library were used. The 4C interactions in different conditions were compared to the control condition (DMSO-treated K562). After that, 4C interactions were classified into gained, lost, or unchanged. Gained, 4C interactions present in the experimental condition but not in the control condition; lost, 4C interaction present in the control condition but not in the experimental condition; unchanged, 4C interactions present in both control and experimental conditions. The proportions of unchanged 4C interactions in different distance categories were calculated as unchanged 4C interactions divided by the total number of 4C interactions in that distance category. Categories with fewer than 3 4C interactions were excluded in this analysis.

### Heatmap and boxplot of RPM signal and ChIP-seq signal at different 4C regions

4C regions are classified as gained, lost, and unchanged according to their presence in experiment versus control condition. For GSK343-treated K562 4C data, the experiment condition is GSK343-treated K562, while the control condition is DMSO-treated K562. Similarly, for CRISPR KO K562 4C data, the experiment condition is either *FGF18*-MRR1-A1 KO or *IGF2*-MRR2-A1 KO clones, while the control condition is the EV clones. Gained, 4C interactions only present in the experiment condition but not in the control condition; Lost, 4C interactions only present in the control condition but not in experiment condition; unchanged, 4C interactions present in both control and experiment conditions. In this comparison, only those 4C interactions with *p* value < 0.05 are considered.

For RPM signal heatmap, different types of 4C regions (gained/lost/unchanged) are first classified according to their H3K27ac/H3K27me3 ChIP-seq signal levels in the control condition. Tertiles are used to classify these 4C regions into high, medium, or low category. The 4C intensity in RPM of each 4C regions are shown in a color-scaled manner.

For the ChIP-seq signal heatmap, ChIP-seq signal different types of 4C regions (gained/lost/unchanged) are calculated as the area of signal at these regions. Deeptools computeMatrix^89^ is used to calculated ChIP-seq signal at these 4C regions, and then the total signal areas are calculated as: Total Signal Area = sum (Sig *BS), where BS is the size of bins used when summarizing RPKM, and Sig is the ChIP-seq signal in RPKM at individual bin. For ChIP-seq signal boxplot, the same 4C region (gained/lost/unchanged) in different conditions are connected using gray line. Wilcoxon paired test are used, and *p* value are indicated accordingly: ns, *p* > 0.05, *p < = 0.05, **p < = 0.01, ***p < = 0.001, ****p < = 0.0001.

### Data deposition

ChIP-seq, 4C-seq, and RNA-seq sequencing data generated in this study have been deposited in GEO with the “GSE133183” accession code.

### Reporting summary

Further information on research design is available in the Nature Research Reporting Summary linked to this article.

## Supplementary information

Supplementary Information

Description of Additional Supplementary Files

Supplementary Data 1

Supplementary Data 2

Supplementary Data 3

Supplementary Data 4

Supplementary Data 5

Supplementary Data 6

Supplementary Data 7

Reporting Summary

## Data Availability

All relevant data supporting the key findings of this study are available within the article and its Supplementary Information files or from the corresponding author on reasonable request. Processed Hi–C interactions in K562, GM12878, and HAP1 were obtained from GEO “GSE63525”. H3K27me3 and H3K27ac ChIP-seq peaks in K562 and GM12878 obtained from ENCODE at UCSC (“wgEncodeEH000031”, “wgEncodeEH000044”, “wgEncodeEH000030”, “wgEncodeEH000043”). Other H3K27me3 ChIP-seq data in H1hESC, HeLaS3, HepG2, and MCF7 are obtained ENCODE (“ENCSR000ALU”, “ENCSR000APB”, “ENCSR000AOL”, and “ENCSR768LHG”). H3K27me3 ChIP-seq data in KARPAS-422, Pfeiffer, and WSU-DLCL2 were obtained from GEO “GSE40970”. EZH2 ChIP-seq data in K562, GM12878, H1hESC, HepG2, and HeLaS3 were obtained from ENCODE (“ENCFF083IDB”, “ENCSR000ARD”, “ENCSR000ASY”, “ENCSR000ARI”, and “ENCSR000ATC”). All other relevant data supporting the key findings of this study are available within the article and its Supplementary Information files or from the corresponding author upon reasonable request. A reporting summary for this article is available as a Supplementary Information file. Source data are provided with this paper.

## Source data

Source Data

## Code availability

The code for MRR calling used in this study can be found here: https://bitbucket.org/YichaoCai/rose_strict_share/src/master/.
