# Supplementary material for: H3K27me3-rich genomic regions can function as silencers to repress gene expression via chromatin interactions
Source: Nat Commun. 2021 Jan 29;12:719. doi: 10.1038/s41467-021-20940-y (PMC7846766; doi:10.1038/s41467-021-20940-y)
Supplement: Supplementary file 1 — Supplementary Information [file 41467_2021_20940_MOESM1_ESM.pdf]

Supplementary Information

Contents:

1. **Supplementary Note I**
2. **Supplementary Tables**
3. **Supplementary Figures**
4. **Supplementary References**

Supplementary Note I

Selection of regions to CRISPR

We found that there are 974 MRRs in K562 cell line and their median size is 92,170bp. We aligned these MRRs with Hi-C anchors and found that there are 560 MRRs associated with interactions. Next, through aligning with gene annotation, we found that there are 237 MRRs which are associated with genes.

These MRRs can be further classified into three different categories: (1) proximal looping (MRR overlap with target gene promoter), (2) distal looping (MRR loops over to the promoter of target gene while not overlap with its promoter) and (3) internal looping (MRR loops over to the promoter of target gene and at the same time overlap with gene promoter). There are 54 internal looping MRRs and since we want to investigate the relationship between H3K27me3, chromatin interactions and gene expression, we chose CRISPR examples from this category.

Firstly, we filtered out some known translocated regions and rearrangements such as *BCR-ABL* and the *IGHV* cluster. Next, we selected 13 MRRs whose H3K27me3 signal rank is lower than 80 and loop number is equal or larger than 10. Among those MRRs, we further chose those contain genes which loop to more than one anchor. Finally, we selected two MRRs associated with cell identity including MRR1 whose target gene is *FGF18* and MRR2 whose target gene is *IGF2* (Supplementary Figure 3a).

Supplementary Tables

Supplementary Table 1. Putative human silencer examples.

Supplementary Table 2. Comparison between different identification methods of human silencers**.**

Supplementary Table 3. Primers used for ChIP-qPCR, RT-qPCR, Sanger Sequencing, 4C-seq and guide RNAs used for CRISPR.

Supplementary Table 4. Genomic coordinates of 4C viewpoints.

Supplementary Table 1. Putative human silencer examples.

Element and locus	Gene
An AT-rich octamer –binding domain located -140bp from TSS	Human thyrotropin-β gene (*hTSHβT*)[2]
This silencer (5’ CTCTCTAGAGAG 3’) is located -1800 bp from the transcription initiation site	plasminogen-activator-inhibitor type-2 gene (*PAI-2*)[3]
PRE2-S5	*CCND1* gene[4]
5’ untranslated region of the first exon	human α1-chimaerin gene[5]
8bp silencer elements (-97 to -90bp) in the 5’ promoter	human Pi Class glutathione S-transferase (*GSTP1*) gene[6]
5’-GGCATAATGGGTCTGTCTCATCGTC-3’ (-211bp to -186bp)	Human interferon-γ gene[7]
MECP2-F3 (in the 210kb region encompassing *MECP2* gene)	*MECP2* gene[8]
5’- TTCAGCACCNCGGACAGNGCC-3’ (-235bp to -199 bp)	Human synapsin I[9]
Not shown	human INK4b-ARF-INK4a locus[10]
A GU-rich region located downstream of splicing site 2	human papillomavirus late mRNA[11]
5’-GGGGAGGGGG-3’ (-1418bp to -1388bp)	platelet-derived growth factor A-chain gene[12]
A 24bp element within the third intron of the human collagen type IV gene	human collagen type IV gene[13]
First intron of the human apolipoprotein A-II gene	human apolipoprotein A-II gene[14]
A 190bp fragment from the first intron of the human *CD4* gene	*CD4* gene[15]
A 1.8-Mb region of human chromosome 7[16]	Not shown
T39, located on the short arm of the X chromosome (p22.2) in the first intron of the gene *ARHGAP6*[17]	Not shown
Not shown	*BDNF* gene in Huntington’s disease[18]

Supplementary Table 2. Comparison between different identification methods of human silencers.

	Huang *et al*[19]	Jayavelu *et al*[20]	Pang and Snyder[21]	Ours
Methods	Authors focused on the DHSs overlapping within H3K27me3 peaks (H3K27me3-DHSs), and then explore their correlation with expression of nearby genes across tissues. In this way, H3K27me3-DHS were categorized into three groups: positively correlated (posCOR), negatively correlated (negCOR) and uncorrelated (unCOR). NegCORs were identified as silencers	Authors considered DHS from 82 human cell lines (ENCODE+Roadmap) or from 22 mouse cell line (ENCODE) and the exclude all that overlapped with chromatin features of enhancers, promoters or insulators. The remaining DHS were filtered for cell-type specificity and considered “putative silencer elements”	Authors used the high-throughput ReSE lentiviral screen system. If silencers (fixed size=200bp) are inserted, cells wont undergo apoptosis	We first identified H3K27me3 peaks through H3K27me3 ChIP-seq data in 7 cell lines, then clustered nearby peaks, and ranked the clustered peaks by average H3K27me3 signals levels. The top clusters with the highest H3K27me3 signal were called H3K27me3-rich regions (MRRs)
Numbers of silencers	1334 per cell line	Each cell type contains tenths of thousand elements	2664 potential silencers in k562 cells	Unique to different cell line, eg:974 MRR in K562
Specificity	Tissue specific	Cell type specific. Silencers can act as enhancers in other cell types	Tissue specific	Cell type specific
TF binding enrichment	Common Repressors: MECOM, SMAD family, FOXG1,	Enriched for binding of REST, YY1, ZBTB33, SUZ12 and EZH2 (based on ChIP-seq)	AP2 binding domains were found in both K562 and	Different silencer clusters have different TFs

		CTCF, TCF and SOX21. Tissue specific TFs: STAT family, EHF, TFAP2A, NKX6-1 and E2F4	and also for motifs of other repressor TFs	HepG2. CDH4 and NCoR enriched in K562 while EZH2 and REST enriched in K562	
	Sequence signatures	Not explored	Many silencers are hypermethylated and poor conservation	Not explored	No difference in terms of CpG islands
	Chromatin interactions	Used Hi-C to link distal genes but only focused on proximal genes	Majority promoter interactions of silencers correspond to non-expressed or lowly expressed genes	ReSE silencers can directly interact with many gene promoters	MRRs are highly associated with chromatin interactions than typical ones and based on the Hi-C, can be classified into distal, internal looping and proximal silencers
	Disease traits (SNPs)	6.7% of silencers harbor at least one GWAS SNP	25% disease traits present are enriched at silencers, similar to other cis-regulatory elements	Not explored	Not explored
	Validation	Five out of ten predicted silencers have decreased luciferase reporter gene activity. Sharpr-MPRA results showed predicted silencers showed repressive impact	51% of 7500 selected silencers in K562 was validated through MPRA	Three silencers were validated using CRISPR	Validate two candidate (*FGF18* and *IGF2*) from internal looping category in K562 by CRISPR excision and RT-qPCR
	Mechanism	Not explored	Not explored	Not explored	Based on the *IGF2* silencer example, chromatin interactions and histone state control the *IGF2* expression.MRR removal cause

					chromatin interactions landscape change. Specifically, more active loops gained and repressed CI lost which finally leads to H3K27me3 loss at *IGF2* region and *IGF2* expression upregulation

Supplementary Table 3. Primers used for ChIP-qPCR, RT-qPCR, Sanger Sequencing, 4C-seq, 3C-PCR and guide RNAs used for CRISPR.

Primer name	Sequence (5’ to 3’)
ChIP-qPCR primers	
MRR1-R1-F	TGGTCCATAATGGCTTATCATCCT
MRR1-R1-R	GAAACACTTGGCTGTCTGGC
MRR1-R2-F	AGCCTGCAGAAATTGACATTGC
MRR1-R2-R	CTCTGGCTCCTTTTGGCTCA
MRR2-R1-F	AAGAGGGGACCCATCAGGG
MRR2-R1-R	TGGGTTTTCTGGAGCCAAGG
MRR2-R2-F	TTGTTAGGGACGTCAGTGGC
MRR2-R2-R	TGGAGGGAAACGGCAAAAGA
HAP1-SE-WT-2ND-F	AGGACAGTTGGGAAGGAGGA
HAP1-SE-WT-2ND-R	CAGGGCTGCTGCACCTAC
HAP1-SE-WT-3RD-F	CGAGTCCTTCCCGGTGAGAT
HAP1-SE-WT-3RD-R	AAGTCCACGGAACAACGACA
HAP1-SS-WT-2ND-F	GTATAACACGCCTGCACAGACA
HAP1-SS-WT-2ND-R	TCCCAATCCACGTGAGGGA
HAP1-SS-WT-4TH-F	CAGAACTTTGCAGCAGGCAG
HAP1-SS-WT-4TH-R	GGAGCAGCAGCATCCACTTA
IGF2-R1-F	CACCGCGTCAACATACCAGG
IGF2-R1-R	CATGTGTGATTCGTGCCTTGC
IGF2-R2-F	AAGCAAGGAAGTCACGGGTC
IGF2-R2-R	GAGAAATAGGGCTTCGGGCG
IGF2-R3-F	GGGCATCTCTGTCATGGTGG
IGF2-R3-R	GGCATTTGGGATACACCCGT
IGF2-R4-F	GCGAGGTAAACCTCCCAGAG
IGF2-R4-R	CGGGTCTGGTGATGCCATAG
IGF2-KOsite-F	GCACTCCAAGAAAAGGCCAG
IGF2-KOsite-R	GACGTCCCTAACAAAGTGCC
IRF2BP2-ChIP-F	GGACAGTGAACAGCGGTCAA
IRF2BP2-ChIP-R	CTGAGAGTGCTGCTGGGAAA
RT-qPCR primers	
GAPDH-F	GCACCGTCAAGGCTGAGAAC
GAPDH-R	GGATCTCGCTCCTGGAAGATG
FGF18-RT-F	GACGATGTGAGCCGTAAGCA
FGF18-RT-R	GAGCTGGGCATACTTGTCCC
UBTD2-RT-F	CACCGGAGTTGCTCTAGGTC
UBTD2-RT-R	TCCCTCTTGCTGCGTAGTTG
FBXW11-RT-F	CCAGTGTCAGATGTCTCCAGATAA
FBXW11-RT-R	AGTTTCCTTCTGATGGCCTCTT
IGF2-RT-F	TCCTGTGAAAGAGACTTCCAGC
IGF2-RT-R	TTGGTGTAGCTCAGCAGAAGG
H19-RT-F	CAGGAGTGATGACGGGTGGAG
H19-RT-R	CCCTTCTTTCCAGCCCTAGCTC
KCNA2-RT-F	ATCCGGTTGGAACGCAGAC

KCNA2-RT-R	AGGTGCAGTCATGTGAGGTG
BARX2-RT-F	TGCATTCCTGTACGGGCTC
BARX2-RT-R	CAGGTGGGAGATGACAGTGG
ADAMTS8-RT-F	AGACTGTCTCCTGGATGCCC
ADAMTS8-RT-R	AAAGATCTGCCTGCACTGCT
SFRP5-RT-F	CTGAAGGGCACTCCTCCTTG
SFRP5-RT-R	CCCATCCCTTAGGCCTTGTG
HOXB7-RT-F	TCGAGCCGAGTTCCTTCAAC
HOXB7-RT-R	TCAGTTCCTGAGCTTCGCAT
HS3ST3A1-RT-F	GTCCAAGAGCAGTTTGGAGC
HS3ST3A1-RT-R	GCCCGAGTTCAGGTTCTCTC
HAS2-RT-F	GGCCGGTCGTCTCAAATTCA
HAS2-RT-R	TCACAATGCATCTTGTTCAGCTC
NTS-RT-F	GAACAGCCCAGCTGAGGAAA
NTS-RT-R	CCTGGATTAACTCCCAGTGTTGA
MEIS1-RT-F	ACGGCATCTACTCGTTCAGG
MEIS1-RT-R	CCATCACCTTGCTCACTGCT
HOXB9-RT-F	TGGGACGCTTAGCAGCTATT
HOXB9-RT-R	CGTACTGGCCAGAAGGAAAC
EPHB6-RT-F	CGGCCAACGGGAAGAAATAAA
EPHB6-RT-R	TGTCACAATGAAGACAAACAGGC
GABRQ-RT-F	CCAGGGTGACAATTGGCTTAA
GABRQ-RT-R	CCCGCAGATGTGAGTCGAT
CDK14-RT-F	CCAAGGAGTTGCTGCTTTTC
CDK14-RT-R	GAATGAACTCCAGGCCATGT
TMEM108-RT-F	TTTCTCCTGAGCCGTCGGA
TMEM108-RT-R	GATTCTGTCCTGGAGTAGAGGG
CD276-RT-F	CTCTGACAGCAAAGAAGATGATGG
CD276-RT-R	TCCTTTGGAGAAGGAGCCCA
SRSF4-RT-F	TCATTCAAGGTCTCGCTCTCG
SRSF4-RT-R	ACCTGGACCGAGATCTACTCT
RAI1-RT-F	GATGCCTCCACACCTACCAC
RAI1-RT-R	GCAGCCTCTTATGTTTGGGAC
SRGAP1-RT-F	CCAACATTGATGCCTGTCC
SRGAP1-RT-R	TCTCATAAACAGGGCCATCC
TRIM13-RT-F	CCTTTCCCACTAGCCGGAGTA
TRIM13-RT-R	TCCATCACATCCTCTGTCTCCT
LIMK1-RT-F	ACCTCAACTCCCACAACT
LIMK1-RT-R	TCTCGCACAGGACGATCC
PCBP1-RT-F	AAAGGCGGGTGTAAGATCAAAG
PCBP1-RT-R	GGCAAATCTGCTTGACACACTC
DUSP12-RT-F	ATCTATGGCGCCTCTTCGTG
DUSP12-RT-R	CTGCATGACAGTGCACCAAC
INTS7-RT-F	ATCCTGTGGCAAGAGCCATC
INTS7-RT-R	TGTGCAGAGAAGTTTGCAGC
VPS45-RT-F	GTACTTGTCAATTCGCCGCC
VPS45-RT-R	ACTCACTATGCCAGTCGTCTC
DLX3-RT-F	CTTACTCGCCCAAGTCGGAA

DLX3-RT-R	TCCTTCACCGACACTGGGT
ZNF639-RT-F	ACCCTTCTCGTTATTCAGATTCCT
ZNF639-RT-R	GGCTGTCTCATAGAACAGACACT
HOXD13-RT-F	GGCACGAGGCCTACATCTC
HOXD13-RT-R	TTAGAGCCACATCCCCTGGA
HBB-RT-F	GAGAACTTCAGGCTCCTGGG
HBB-RT-R	GCGAGCTTAGTGATACTTGTGG
HBZ-RT-F	CCGGTCAACTTCAAGCTCCT
HBZ-RT-R	CTCAGCGGTACTTCTCGGTC
HBE1-RT-F	TCACTAGCAAGCTCTCAGGC
HBE1-RT-R	AAACAACGAGGAGTCTGCCC
IRF2BP2-F	GGCCCTTCGAGAGCAAGTTT
IRF2BP2-R	CTTGCAACTGCTTTAGACCCG
CRISPR genotyping primers and guide RNAs	
CRISPR-step2-F	GCCTTTTGCTGGCCTTTTGCTC
CRISPR-step2-R	CGGGCCATTTACCGTAAGTTATGTAACG
MRR1-gRNA-F-F	CACCGTTTTTCCTGTCACGCTGCTC
MRR1- gRNA-F-R	AAACGAGCAGCGTGACAGGAAAAAC
MRR1- gRNA-S-F	CACCGCTCTGCTTTAAGAGCATCAC
MRR1- gRNA-S-R	AAACGTGATGCTCTTAAAGCAGAGC
MRR2- gRNA-F-F	CACCGCTTGGAAGAGGGGACCCATC
MRR2- gRNA-F-R	AAACGATGGGTCCCCTCTTCCAAGC
MRR2- gRNA-S-F	CACCGGGGCTCAAGGGCATGCTACG
MRR2- gRNA-S-R	AAACCGTAGCATGCCCTTGAGCCCC
MRR1-flanking-F	TGGGCTTTTTCTTAAGCTGCC
MRR1-flanking-R	TGGCCAAGTTGATTGTGTTAGT
MRR1-internal-F	CACCTCAACAAACCGATCACC
MRR1-internal-R	GAATCTAATAGCTGAGGACGAGC
MRR2-flanking-F	GATTCGGCCCCTACTTGGAT
MRR2-flanking-R	GTGGTTTGTAGAGGGGTGGC
MRR2-internal-F	CCCCTGACAGAGGGGCA
MRR2-internal-R	GGTGGTTTGTAGAGGGGTGG
4C Primers	
IGF2-Outer-F	TCTCACGGAGCATCTGTCC
IGF2-Outer-R	TTACAGAGCTAGCACCTGGG
IGF2-Nest-F	GAAGCCCACCTTCCACTCA
IGF2-Nest-R	GGCTGGTCTCGACAACAAAG
FGF18-Outer-F	TCAGGCCTCCTTGCAAGCTAT
FGF18-Outer-R	GAACGAAGCTGCCTAGTATGC
FGF18-Nest-F	ACATCGGACACAAGTGCAGAA
FGF18-Nest-R	TCCAAAGCTGGCTCGCCTA
MRR1-Outer-F	CTGGCAGAACGGTTTAGACT
MRR1-Outer-R	CAGGGAGGTTACTGCACTTCAT
MRR1-Nest-F	CCTCCATACTTTCCCAGGGC
MRR1-Nest-R	AAATCCTTGTTCAATGCTTCCC
MRR2-Outer-F	GACCCTTGACTTGGGAGTGG

MRR2-Outer-R	CATGCCAGGCCTTTCAACTG
MRR2-Nest-F	GTTTGCATTCACACGCCCTC
MRR2-Nest-R	GCCCTGTGGGTTTGAGAAACT
HOXD13-Outer-F	TCATCGGCCATTTCCCTGAG
HOXD13-Outer-R	TATCTTACTGGCGACCGTGG
HOXD13-Nest-F	GTCAACTGCTCTGTGCAGACTG
HOXD13-Nest-R	TCTTACTGGCGACCGTGGAC
MYC-Outer-F	TGAAAGAATAACAAGGAGGTGGC
MYC-Outer-R	AGAAGGTCCGAAGAAAGAGGA
MYC-Nest-F	ATGGAGAACCGGTAATGGCA
MYC-Nest-R	AAGGAGGTGGCTGGAAACTT
PSMD5-Outer-F	AAAATGAGGAAGACTTGGCTTGC
PSMD5-Outer-R	GCAGTTACCACATGATTGCAACT
PSMD5-Nest-F	TTTGGCCTTGGACAGATAAT
PSMD5-Nest-R	GTTGTCACGATGTAACTTGAAC
TOR1A-Outer-F	TGAAATGAGTGAGCCCGGAAA
TOR1A-Outer-R	AACCCGATGACATCCAGGAAG
TOR1A-Nest-F	TGCGTGGAGCAGTTAATACC
TOR1A-Nest-R	GTTCAGACCACCCTCGTAAAT
LINC00910-Outer-F	GACCAAATGCACCAAGAGGG
LINC00910-Outer-R	ACCGTGACCCAAACTCTCAT
LINC00910-Nest-F	TCTGAAGGTACACAGTGACCA
LINC00910-Nest-R	TCCATTCATGTCACAGGTGGA
ZDHHC11-Outer-F	TGGTGTCACATTTAGAGGACCA
ZDHHC11-Outer-R	AAACAGGACCATGGTTCTTTGG
ZDHHC11-Nest-F	TCCCAAAGAGACAACAAGGACT
ZDHHC11-Nest-R	TCGCCAAGTCACATTGGTAAAA
CCND3-Outer-F	CACGTATTGTCTCCCCACTTT
CCND3-Outer-R	TGGTCGGTGTAGATGCACAG
CCND3-Nest-F	TATTGTCTCCCCACTTTCCAGG
CCND3-Nest-R	TGGGGACGCAAGACAGGTAG
TMCO4-Outer-F	CTACGCCTCAGTTTGCTGC
TMCO4-Outer-R	CCGTACCTACCATCACCCTG
TMCO4-Nest-F	GCAGTGGCTCACACCTGTAA
TMCO4-Nest-R	TGTAAAATGCCCAGCACAAT
3C-PCR primers	
IGF2-promoter-3C-F	GTCACACTTGAGCACCTCCTGGTAACTG
F1-R	CATGGAGGTTGGCCTCGTTCTTCTTG
F2-R	CTAACCTGACCTGCTCCTTCGACATCTA

Supplementary Table 4. Genomic coordinates of 4C viewpoints.

Viewpoint region	Genomic coordinates
IGF2 promoter	chr11:2175368-2175873
FGF18 promoter	chr5:170845209-170845956
HOXD13 promoter	chr2:176961136-176961617
MRR1-A1	chr5:171338492-171339433
MRR2-A1	chr11:2042072-2043745
MYC promoter	chr8:128,748,315-128,753,680
PSMD5 promoter	chr9:123,578,332-123,605,299
TOR1A promoter	chr9:132,575,221-132,586,441
LINC00910 promoter	chr17:41,299,393-41,546,115
ZDHHC11 promoter	chr5:655,809-883,388
CCND3 promoter	chr6:41,902,671-41,909,552
TMCO4 promoter	chr1: 20127601-20133430

Supplementary Figures

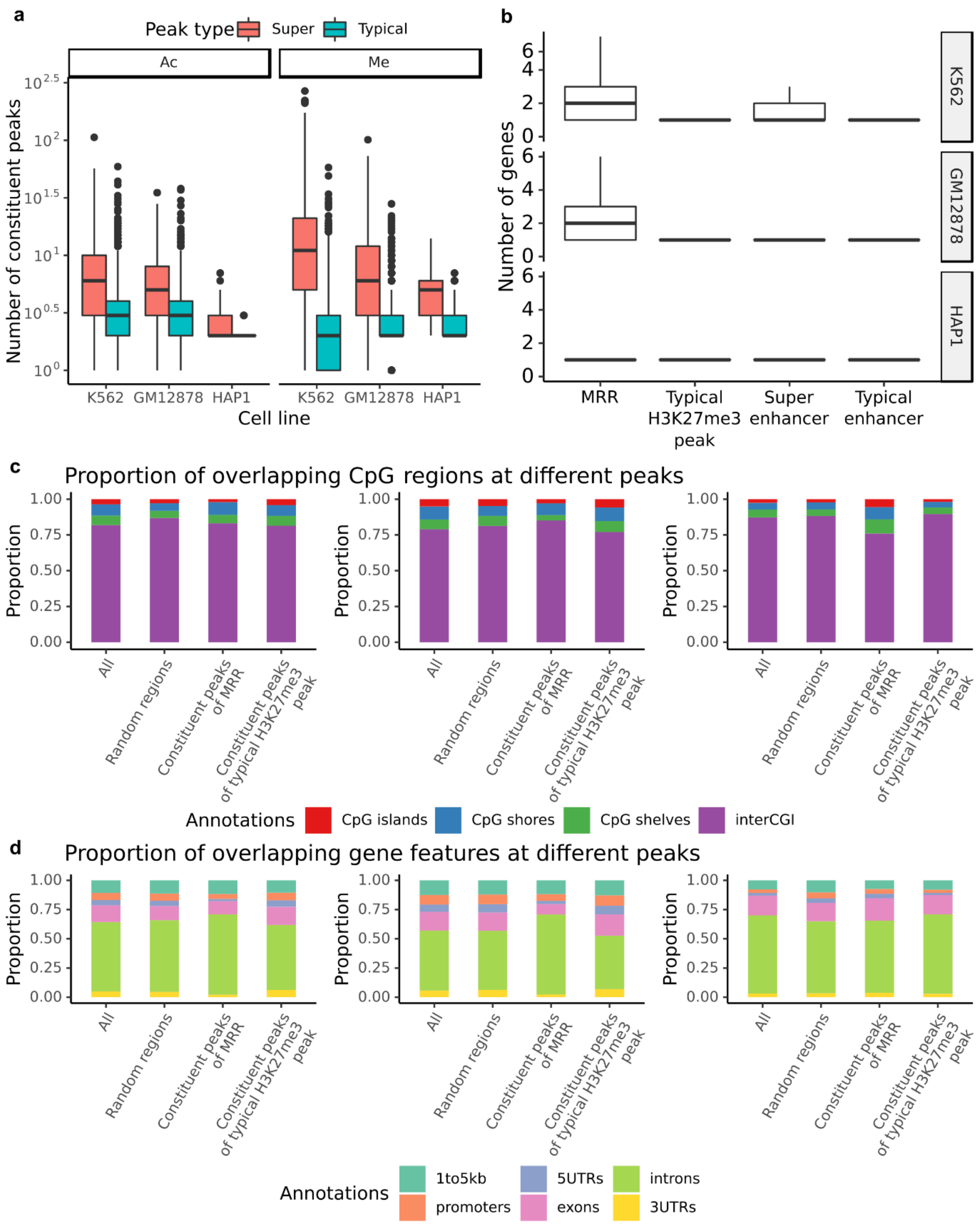

Supplementary Figure 1. Identification of H3K27me3-rich regions (MRRs). **a.** Number of constituent peaks in typical H3K27me3 peaks, MRRs, typical enhancers, and super-enhancers in K562, GM12878, and HAP1 cells, respectively. For K562:

typical H3K27me3 peak (n=41339), MRR (n=974), typical enhancer (n=41729), super-enhancer (n=1225); For GM12878: typical H3K27me3 peak (n=26935), MRR (n=900), typical enhancer (n=40199), super-enhancer (n=1546); For HAP1: typical H3K27me3 peak (n=27034), MRR (n=709), typical enhancer (n=7753), super-enhancer (n=467). **b.** Number of overlapping genes at typical H3K27me3 peaks, MRRs, typical enhancers, and super-enhancers in K562, GM12878, and HAP1 cells, respectively. The sample number are the same as in **Supplementary Figure 1a**. **c.** Proportion of constituent peaks of typical H3K27me3 and constituent peaks of MRR that were overlapped with CpG island. All, all the constituent peaks including typical H3K27me3 peaks and MRRs; random regions, randomly shuffled regions of all the constituent peaks. **d.** Proportion of constituent peaks of typical H3K27me3 and constituent peaks of MRR that were overlapped with different gene features. All and random regions were generated as described in **Supplementary Figure 1c**. Box and whiskers plot: whiskers were extended to the furthest value that is no more than 1.5 times the inter-quartile range. The boxes represent the 25th percentile, median, and 75th percentile.

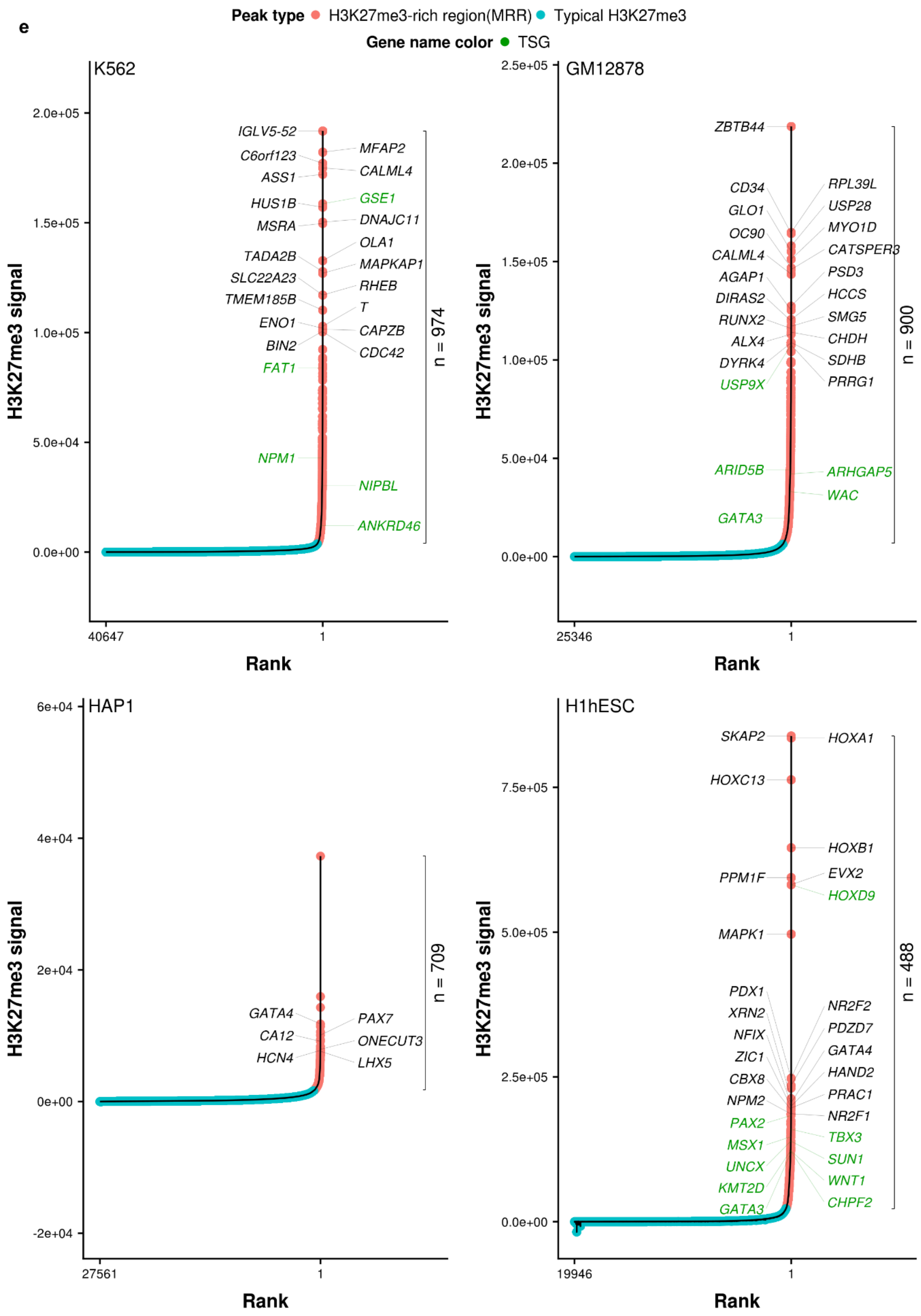

Supplementary Figure 1. **e.** H3K27me3-rich regions (MRR) and typical H3K27me3 peaks and their associated genes in K562, GM12878, HAP1, and H1hESC. One of the genes that had TSS overlapped with top 10 MRR were shown here. TSG, predicted tumor suppressor genes by TUSON.

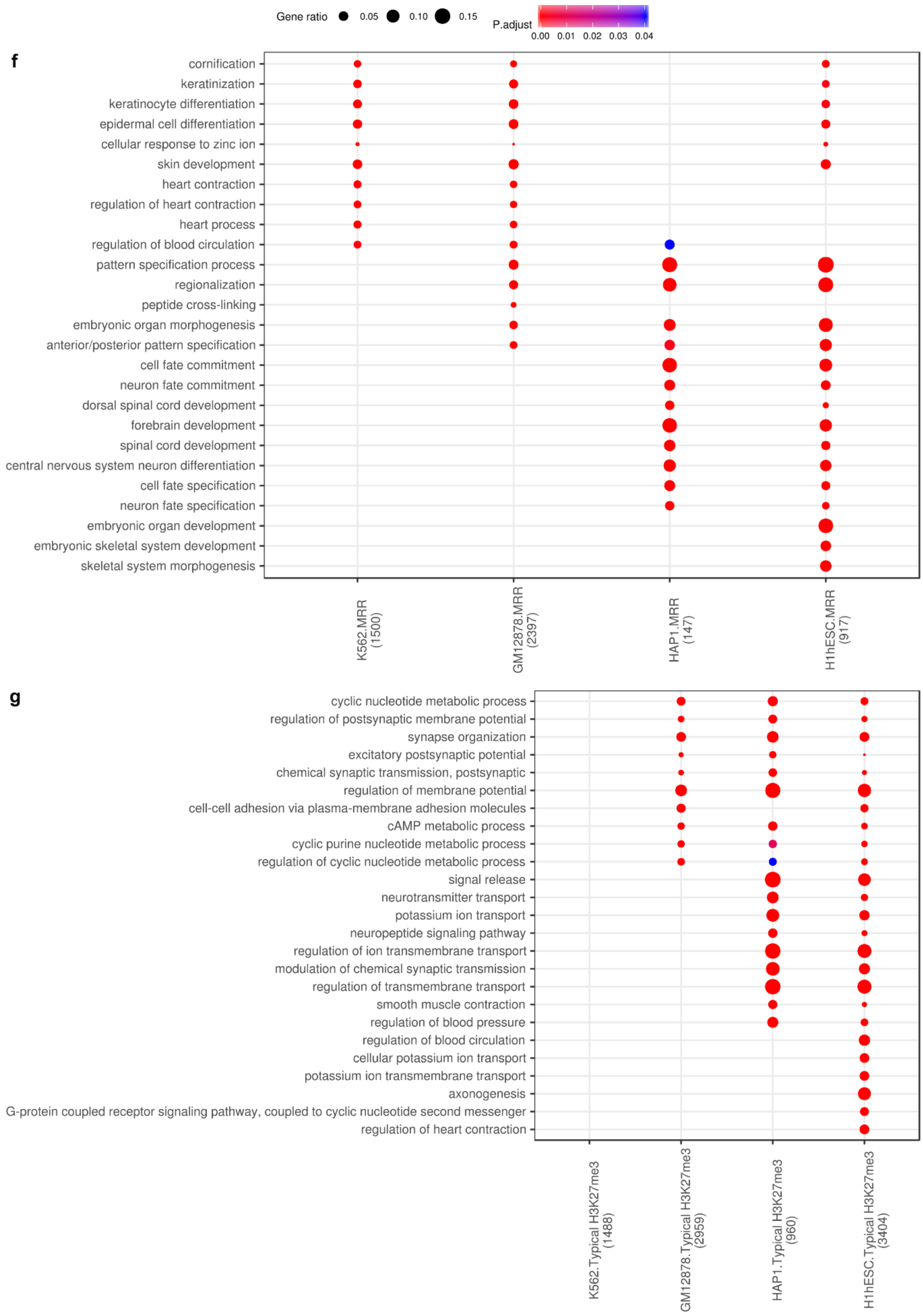

Supplementary Figure 1. f & g. Gene ontology analysis of gene associated with MRR (F) or typical H3K27me3 (G) in K562, GM12878, HAP1 and H1hESC.

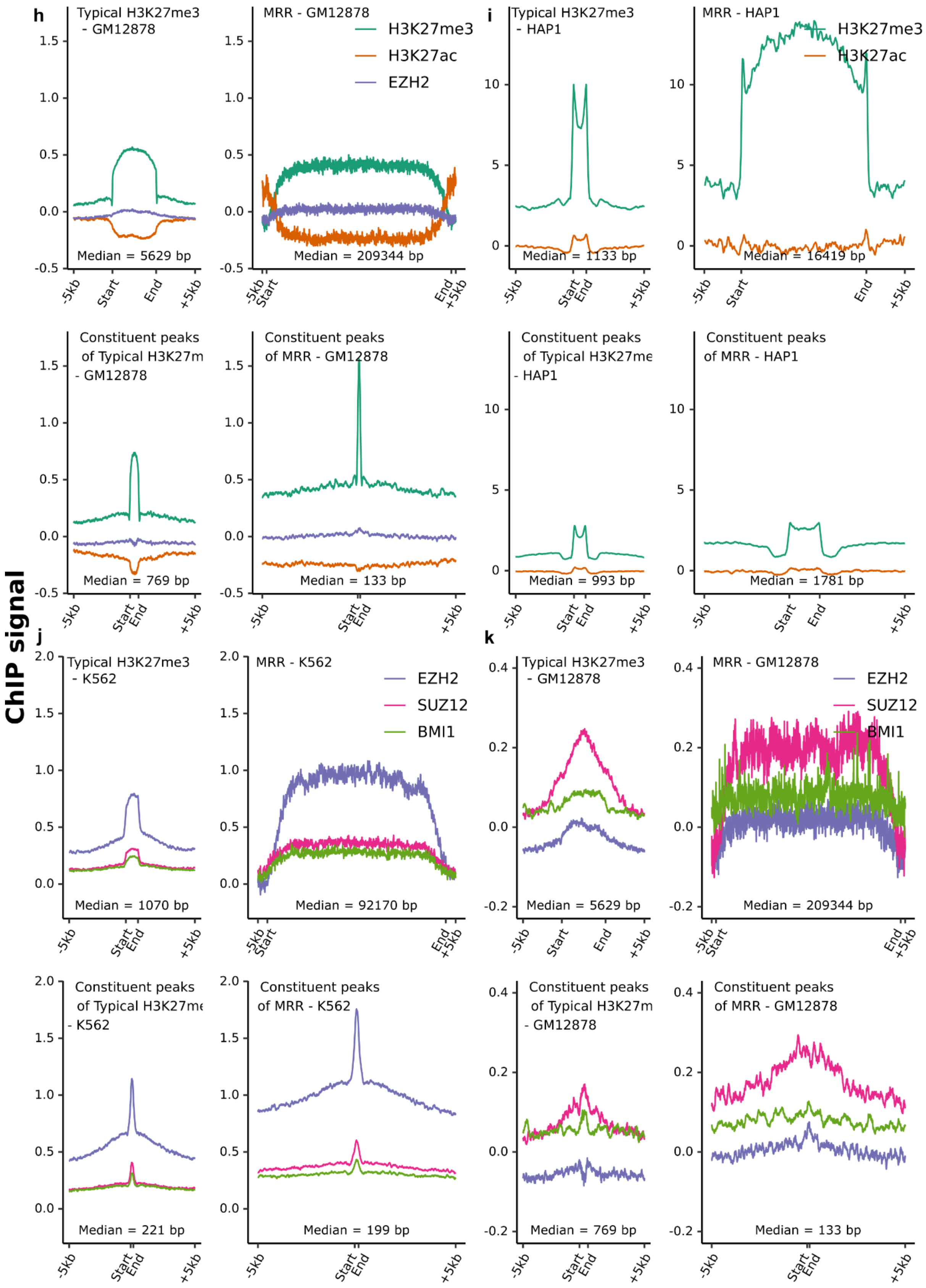

Supplementary Figure 1. h & i. H3K27me3, H3K27ac, and EZH2 ChIP-seq signal on typical H3K27me3, MRR, constituent peaks of typical H3K27me3 peaks, and constituent peaks of MRRs in GM12878 and HAP1. Peaks were scaled to the same median length of peaks in typical H3K27me3, MRR or constituent peaks, and the

ranges were expanded by 5kb on both sides of the peaks. **j & k.** EZH2, SUZ12, and BMI1 ChIP-seq signal on typical H3K27me3, MRR, constituent peaks of typical H3K27me3 peaks, and constituent peaks of MRRs in K562 and GM12878.

I

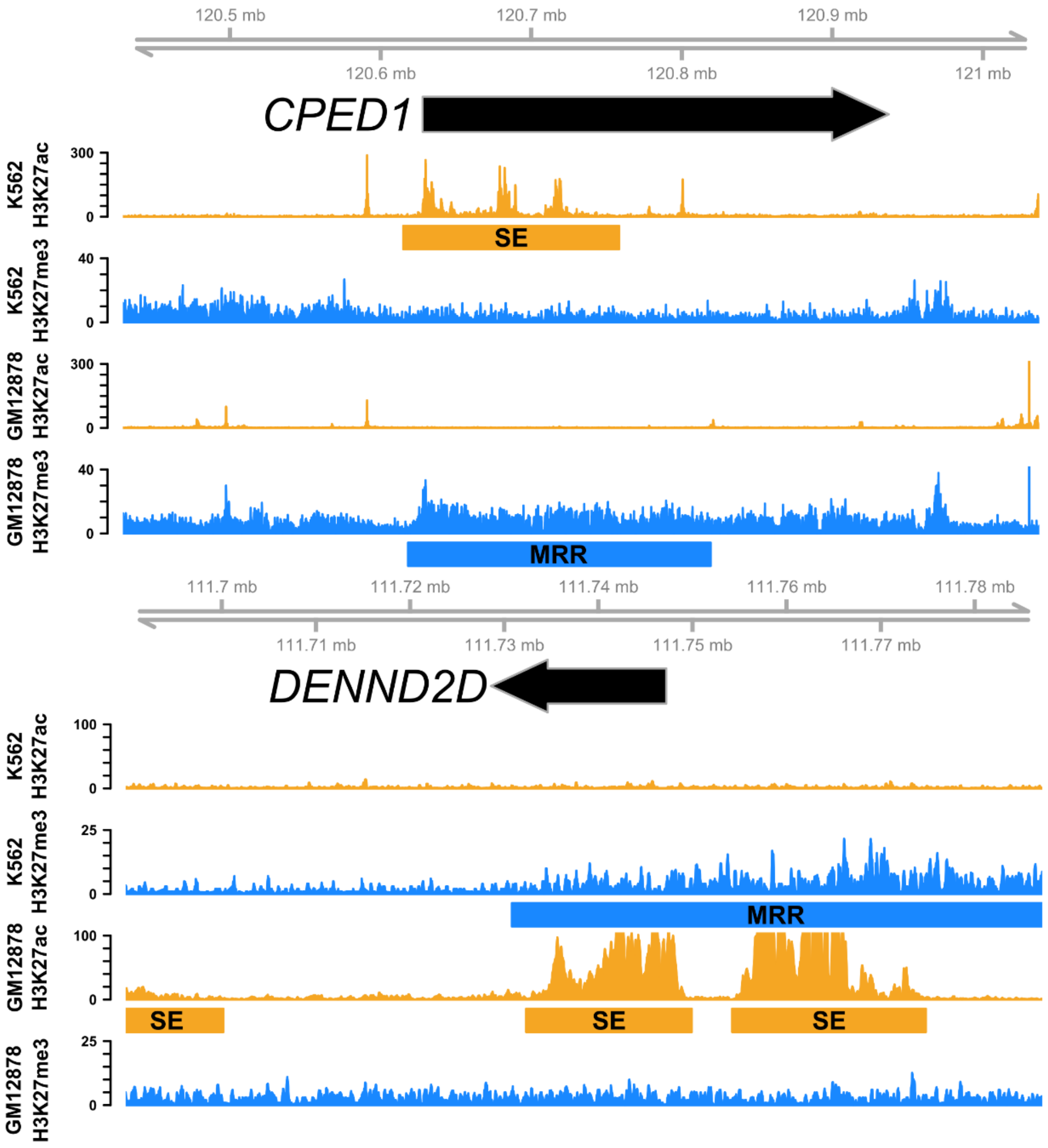

Supplementary Figure 1. I. Example of CPED1 and DENND2D and their associated MRR/SE in K562 and GM12878 cell lines. MRR and SE could be interchangeable in different cell lines. SE, super enhancers; MRR, H3K27me3-rich regions. Expression

level of CPED1 is 107.826 and 0.029 in K562 and GM12878, respectively; expression level of DENND2D is 0.002 and 78.004 (expression in RPKM).

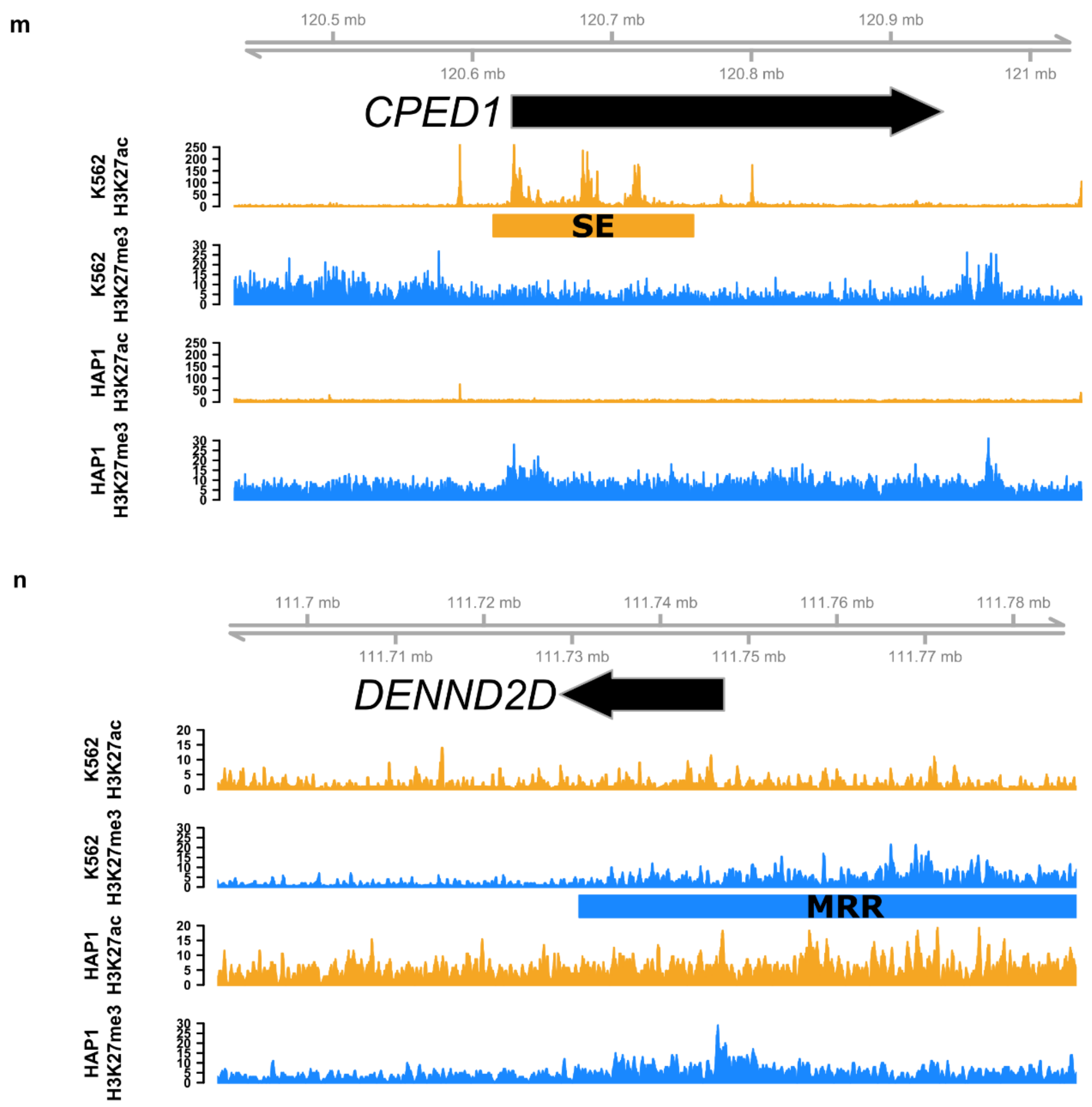

Supplementary Figure 1. m & n. Examples of CPED1 and DENND2D and their associated MRR/SE in K562 and HAP1 cells. SE, super enhancers; MRR, H3K27me3-rich region. Expression level of CPED1 is 107.826 and 0.67 in K562 and HAP1, respectively; expression level of DENND2D is 0.002 and 0.14 (expression in RPKM).

o

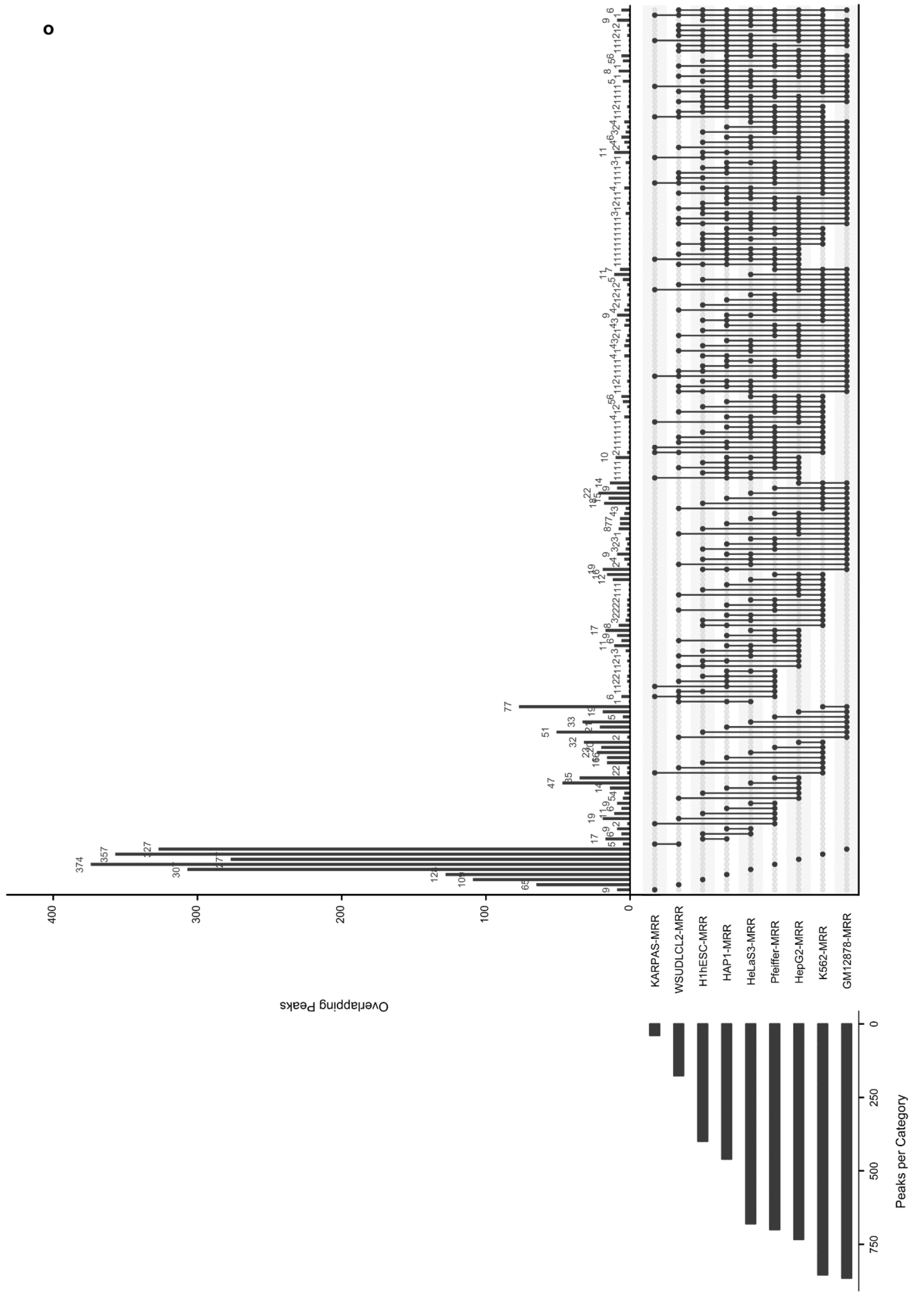

Supplementary Figure 1. **o.** UpSet plot of how MRRs overlap with each other in different cells. HOMER mergePeak command is used to overlap MRRs in different cells.

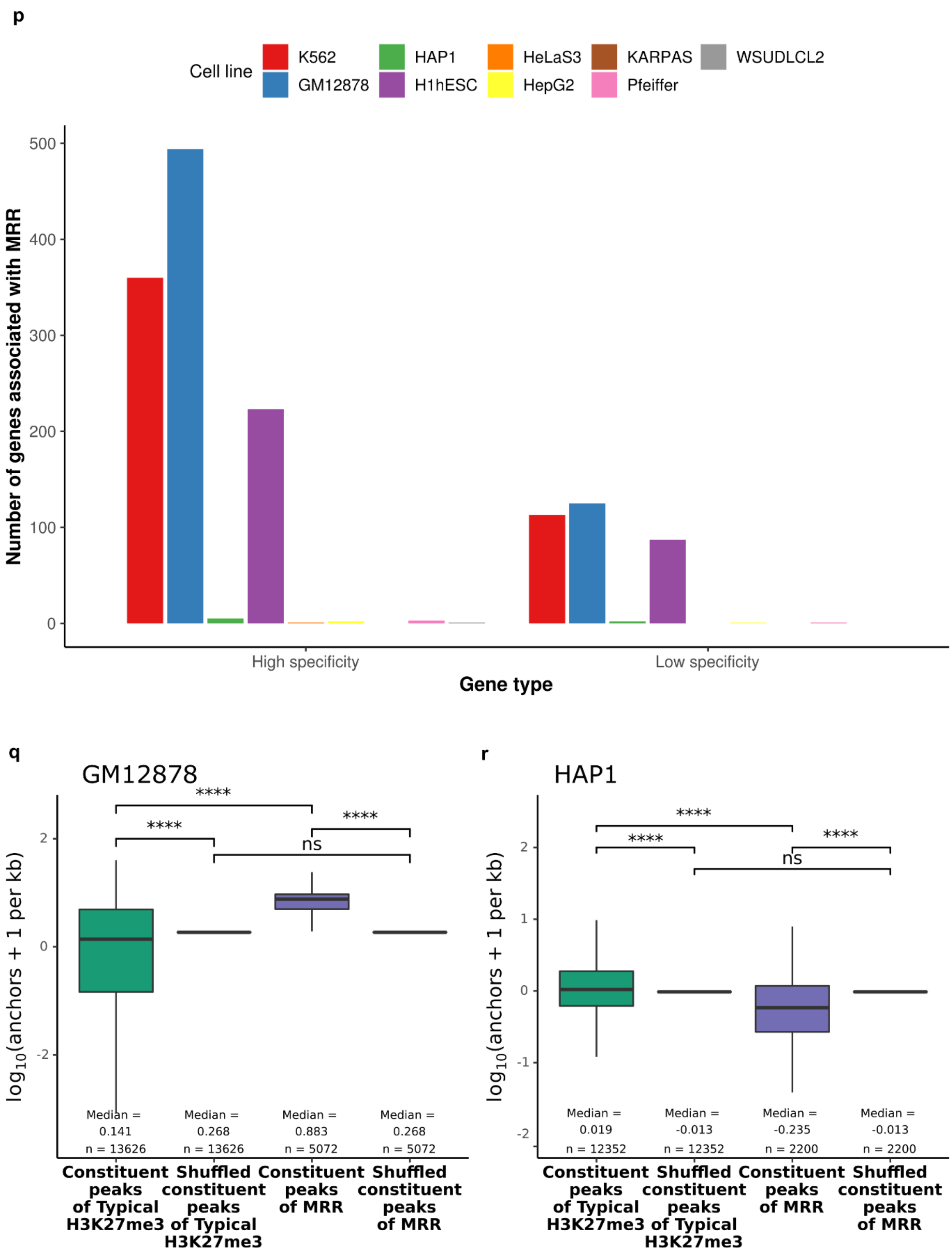

Supplementary Figure 1. **p.** Number of genes with different expression specificities that are associated with MRR. Gene expression in each cell line are compared with 69 facets of tissue/cell expression data from FANTOM. Specificity of each gene X is calculated by: Specificity(X)=1−(entropy(X)/log2(N)). Quartile Q1 and Q3 are used as

cut-off of ‘Low specificity’ and ‘High specificity’, respectively. **q & r.** Density of Hi-C anchors overlapped with constituent peaks of typical H3K27me3 peaks and constituent peaks of MRRs in GM12878 and HAP1 cells. The shuffled peaks were generated by expanding the midpoint of each constituent peaks to the median length of all the constituent peaks, and then followed by random genomic region shuffling. Two-tailed Wilcoxon test p values are as indicated. Box and whiskers plot: whiskers were extended to the furthest value that is no more than 1.5 times the inter-quartile range. The boxes represent the 25th percentile, median, and 75th percentile. *: p <= 0.05; **: p <= 0.01; ***: p <= 0.001; ****: p <= 0.0001; ns: p > 0.05. (For GM12878, constituent peaks of Typical H3K27me3 vs. shuffled constituent peaks of typical H3K27me3: p=1.8e-67; constituent peaks of Typical H3K27me3 vs. constituent peaks of MRR: p=1.4e-157; constituent peaks of MRR vs. shuffled constituent peaks of MRR: p= 2.6e-120; shuffled constituent peaks of typical H3K27me3 vs. shuffled constituent peaks of MRR: p=0.67; For HAP1, constituent peaks of Typical H3K27me3 vs. shuffled constituent peaks of typical H3K27me3: p= 6.7e-19; constituent peaks of Typical H3K27me3 vs. constituent peaks of MRR: p= 1.4e-157; constituent peaks of MRR vs. shuffled constituent peaks of MRR: p= 2.6e-120; shuffled constituent peaks of typical H3K27me3 vs. shuffled constituent peaks of MRR: p=0.67)

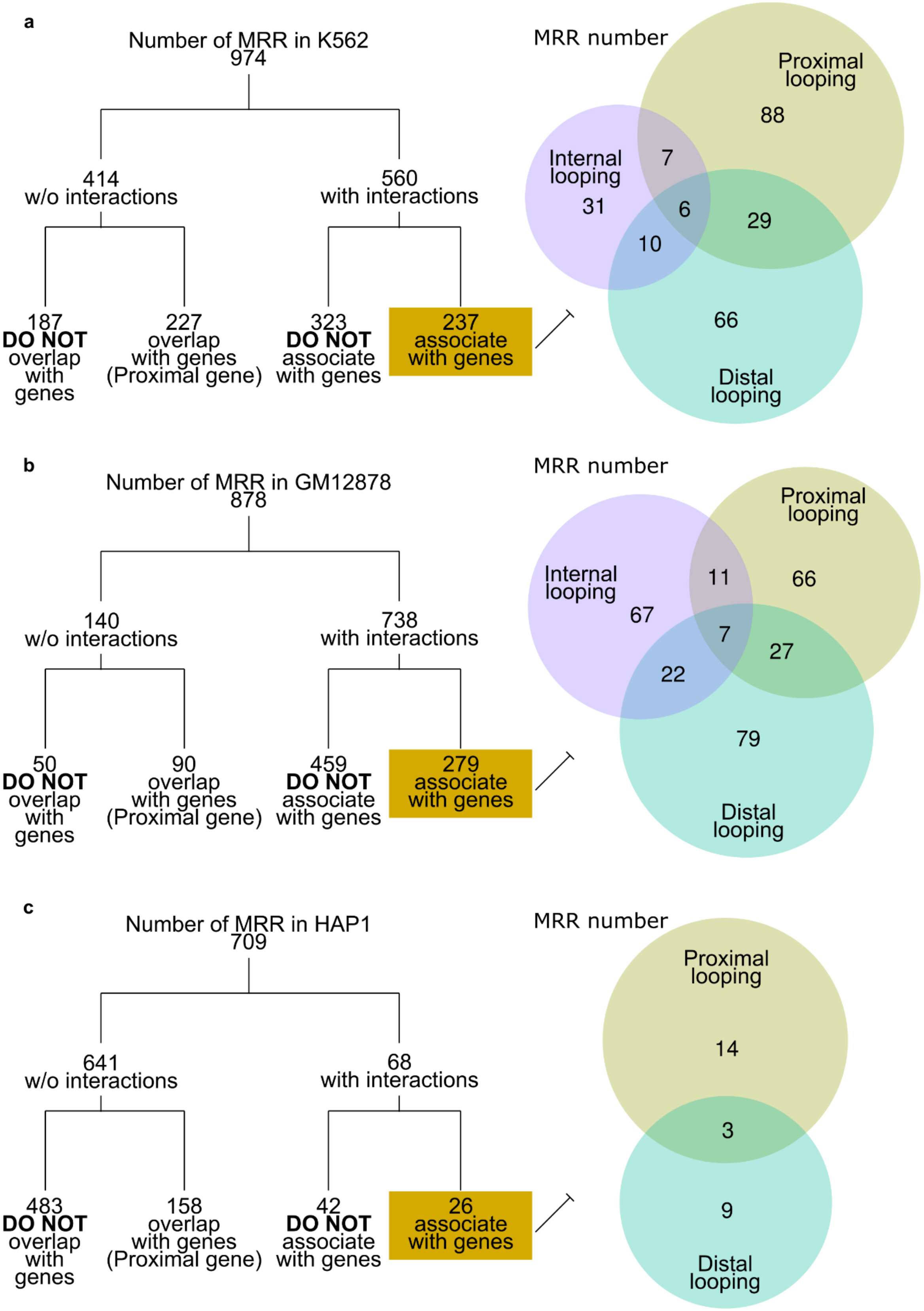

Supplementary Figure 2. Analysis of chromatin interactions associated with MRRs. **a, b & c.** Number of MRRs that involved in chromatin interactions and are associated with genes in K562, GM12878, and HAP1 cells. Venn diagrams showed number of

MRR associated with genes through chromatin interactions in different scenarios described in **Figure 2c**.

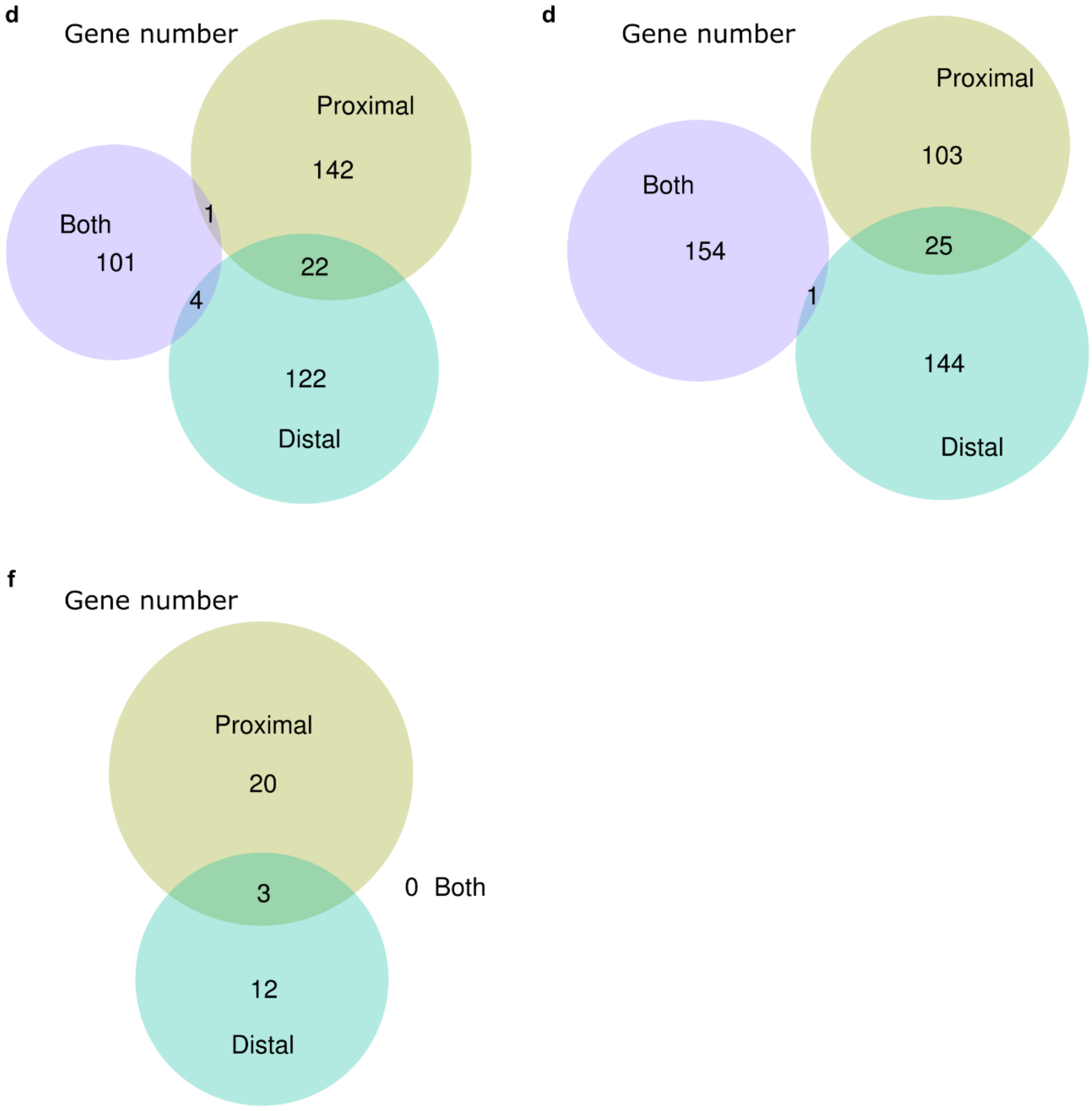

Supplementary Figure 2. d, e & f. Number of genes that are associated with MRRs in different positional relationships. These numbers were not necessarily the same as

the numbers in Supplementary Figure 2a-2c, because the numbers were genes and MRRs, respectively.

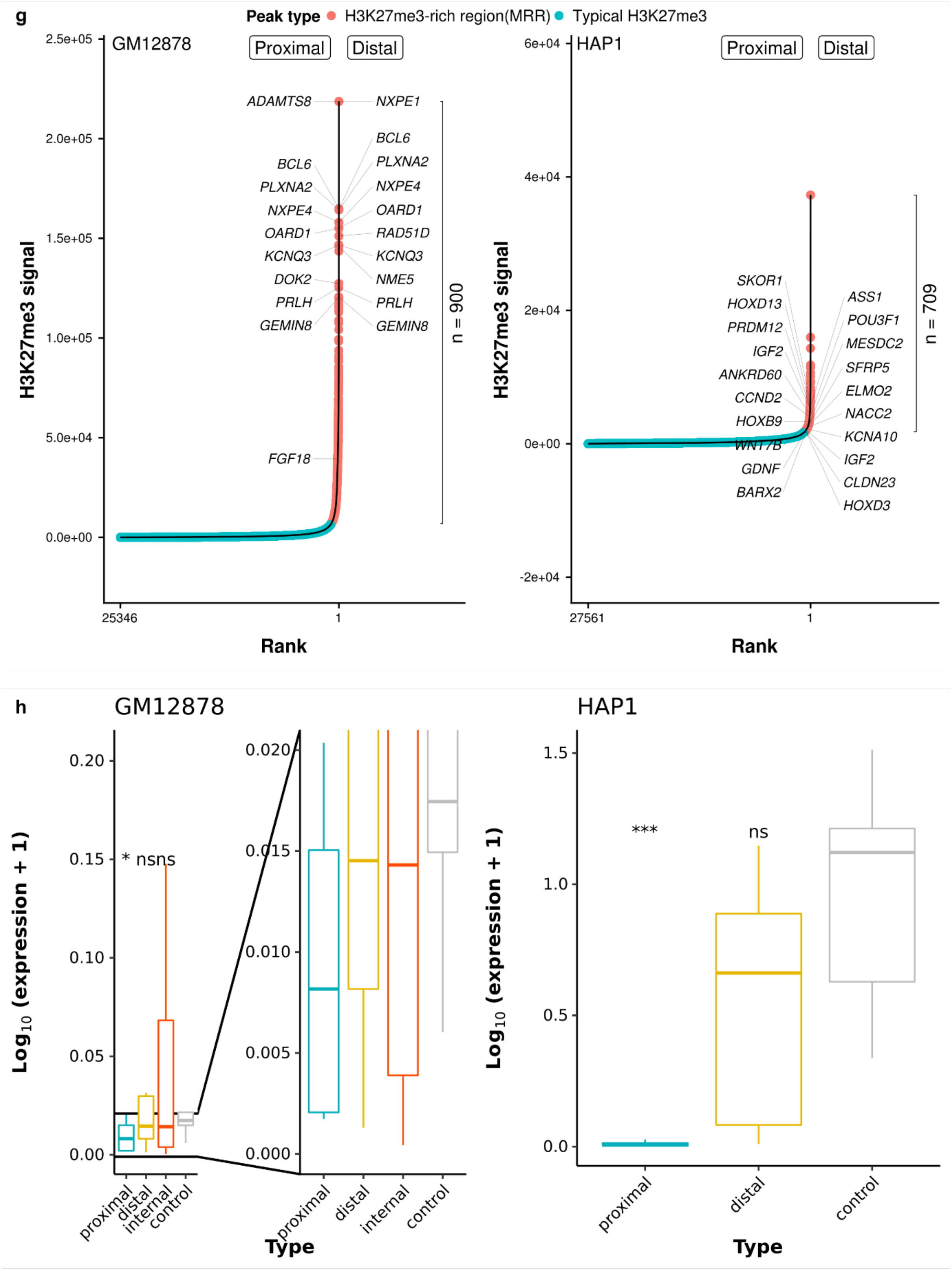

Supplementary Figure 2. **g.** H3K27me3-rich regions (MRR) and typical H3K27me3 peaks in GM12878 HAP1, and their associated genes through chromatin interactions. Proximal, gene and peak occupy the same Hi-C anchor, or gene with promoter overlapping peak; Distal, the peak is connected to the gene via Hi-C interactions (they

occupy different anchors of a Hi-C interaction). **h.** Expression of genes that are associated with MRR in proximal, distal, and internal category in GM12878 and HAP1 cells. The three categories are described in **Figure 2c**. The control category is generated by: 1) first filter out genes that are overlapped with ENCODE blacklist regions and also H3K9me3 peaks; 2) only retain genes that are overlapped with Hi-C interactions; 3) randomly sample the same amount of genes as the average gene number in proximal/distal/internal category. For GM12878, proximal (n=36, p=0.066), distal (n=31, p=0.93), internal (n=63, p=0.93), control (n=43). For HAP1, proximal (n=22, p=0.00064), distal (n=10, p=0.16), internal (n=0), control (n=10). Wilcoxon test p values are indicated, ns: p > 0.05, *: p <= 0.05, **: p <= 0.01, ***: p <= 0.001, ****: p <= 0.0001. Box and whiskers plot: whiskers were extended to the furthest value that is no more than 1.5 times the inter-quartile range. The boxes represent the 25th percentile, median, and 75th percentile.

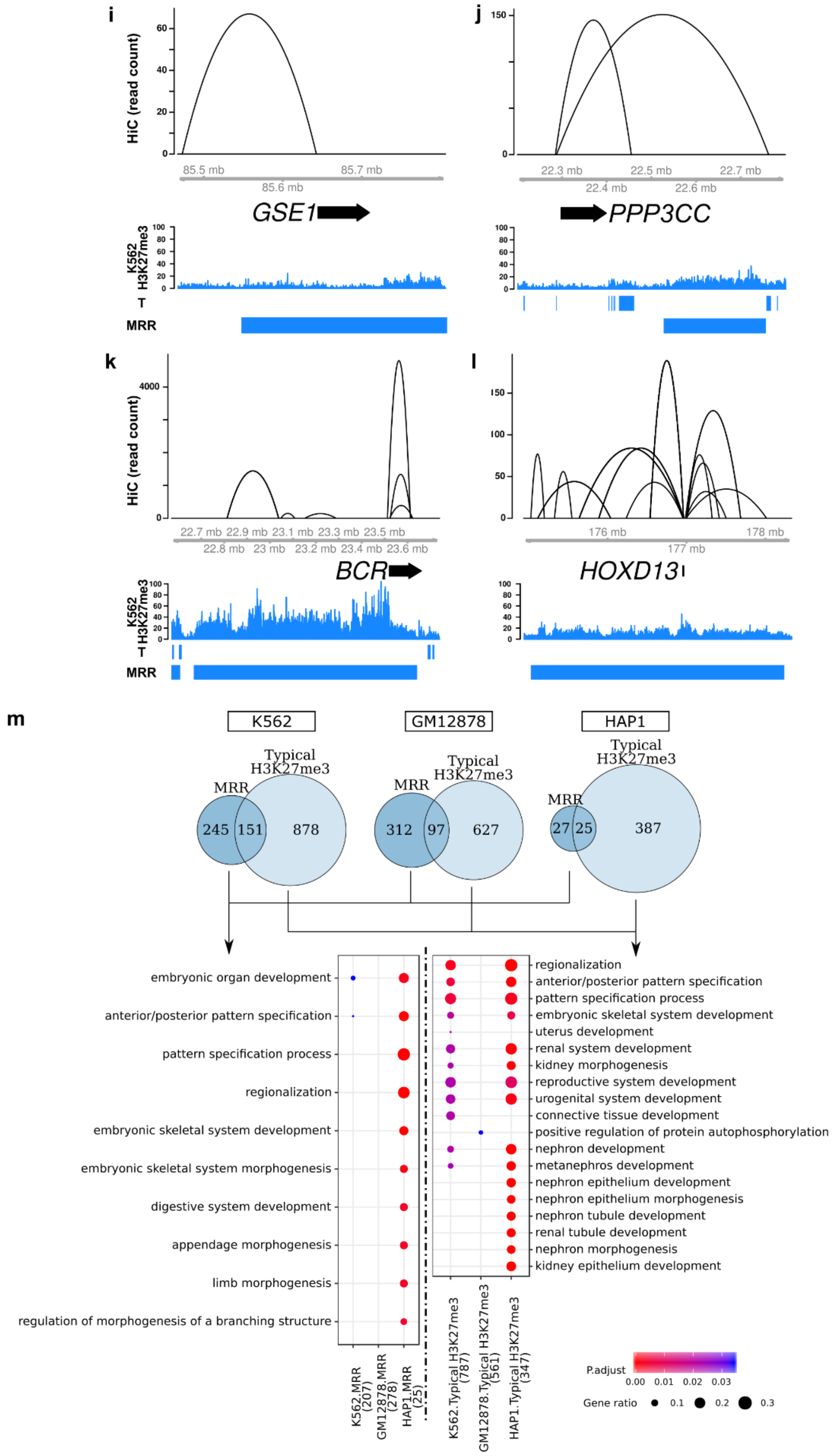

Supplementary Figure 2. i, j, k & l. Examples of genes that were associated with MRRs in different categories as described in **Figure 2c**. **m.** Gene ontology analysis of MRR and typical H3K27me3 associated genes through chromatin interactions.

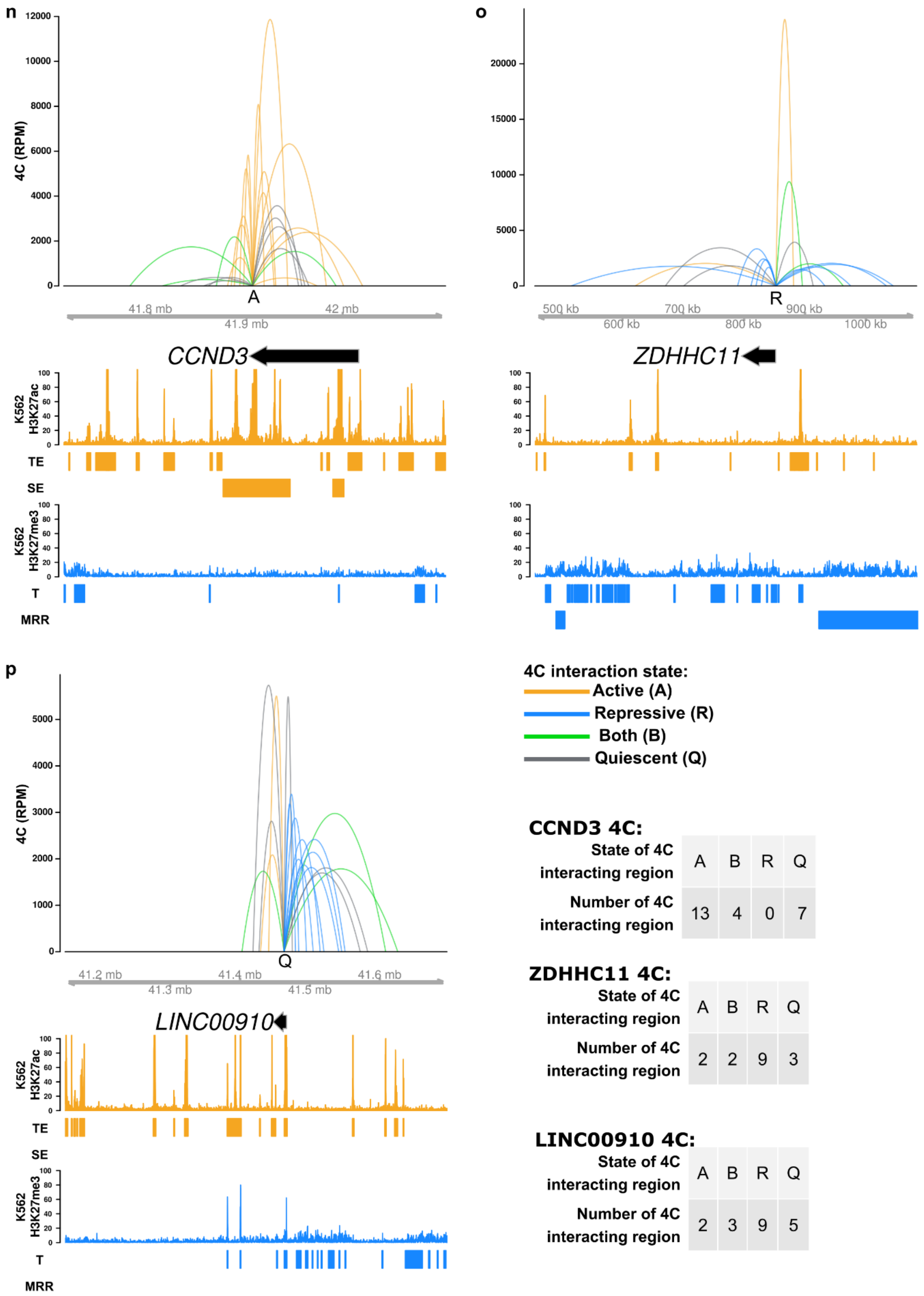

Supplementary Figure 2. n, o & p. Examples of 4C interactions of different candidate regions of different states in K562 cells. The colors of 4C interactions are based on the distal interacting regions to the 4C bait. Blue: repressive; orange: active; green:

both; grey: quiescent. The state of the 4C bait is labeled by text. The numbers of different states of 4C interactions were given in table.

q

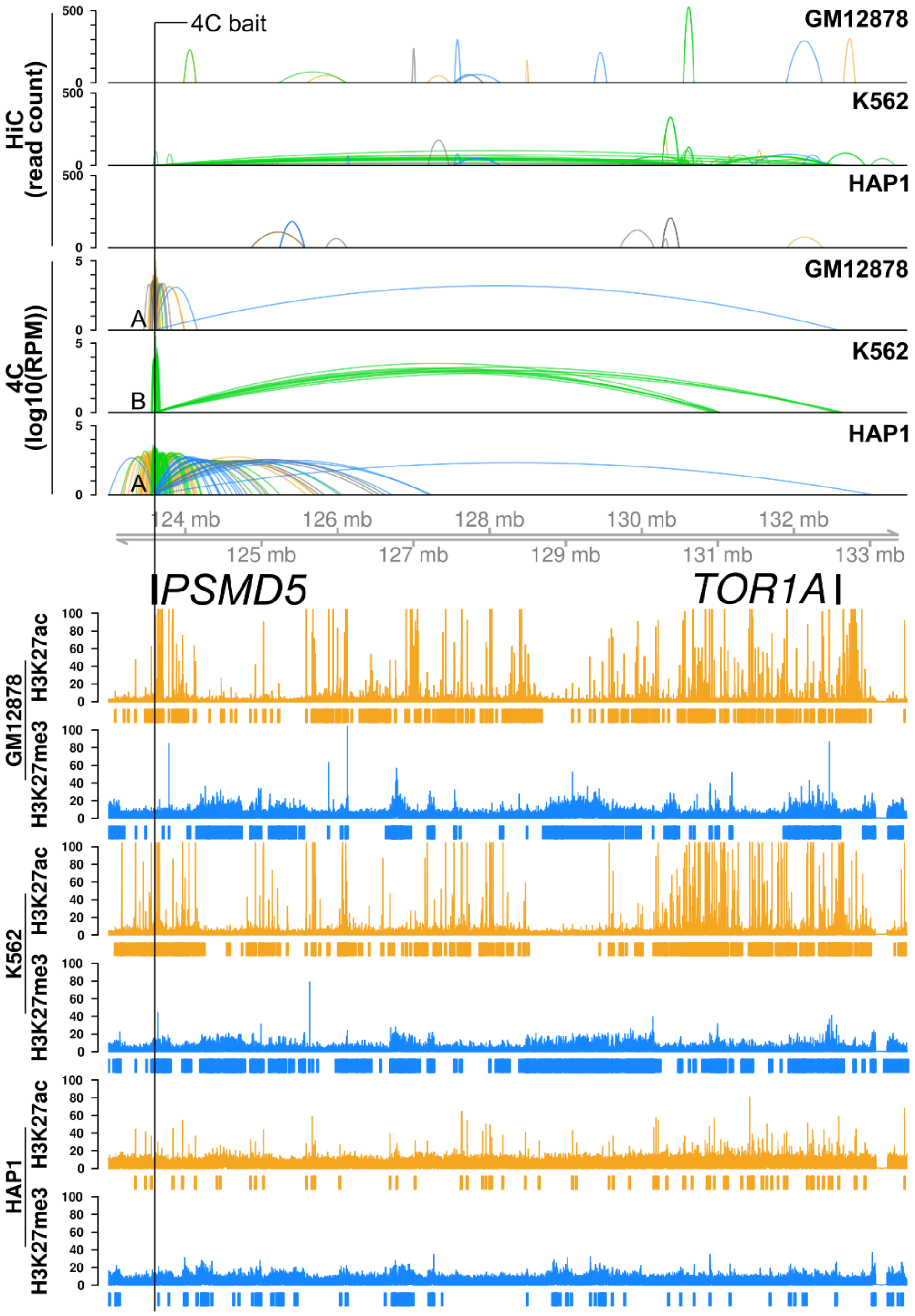

Number of different states of 4C interactions:

	A	B	R	Q
**GM12878**	15	1	2	10
**K562**	0	29	0	0
**HAP1**	39	49	17	7

Supplementary Figure 2. **q.** Validation of Hi-C interactions using *PSMD5* as 4C bait. Extensive ‘B-B’ interactions (green arcs) connecting *PSMD5* gene are shown in Hi-C and validated by 4C in K562 cells. The colors of Hi-C and 4C interactions are based on the distal interacting regions to the gene’s promoter and the 4C bait, respectively.

Blue: repressive; orange: active; green: both; grey: quiescent. The numbers of different states of 4C interactions were given in table.

r

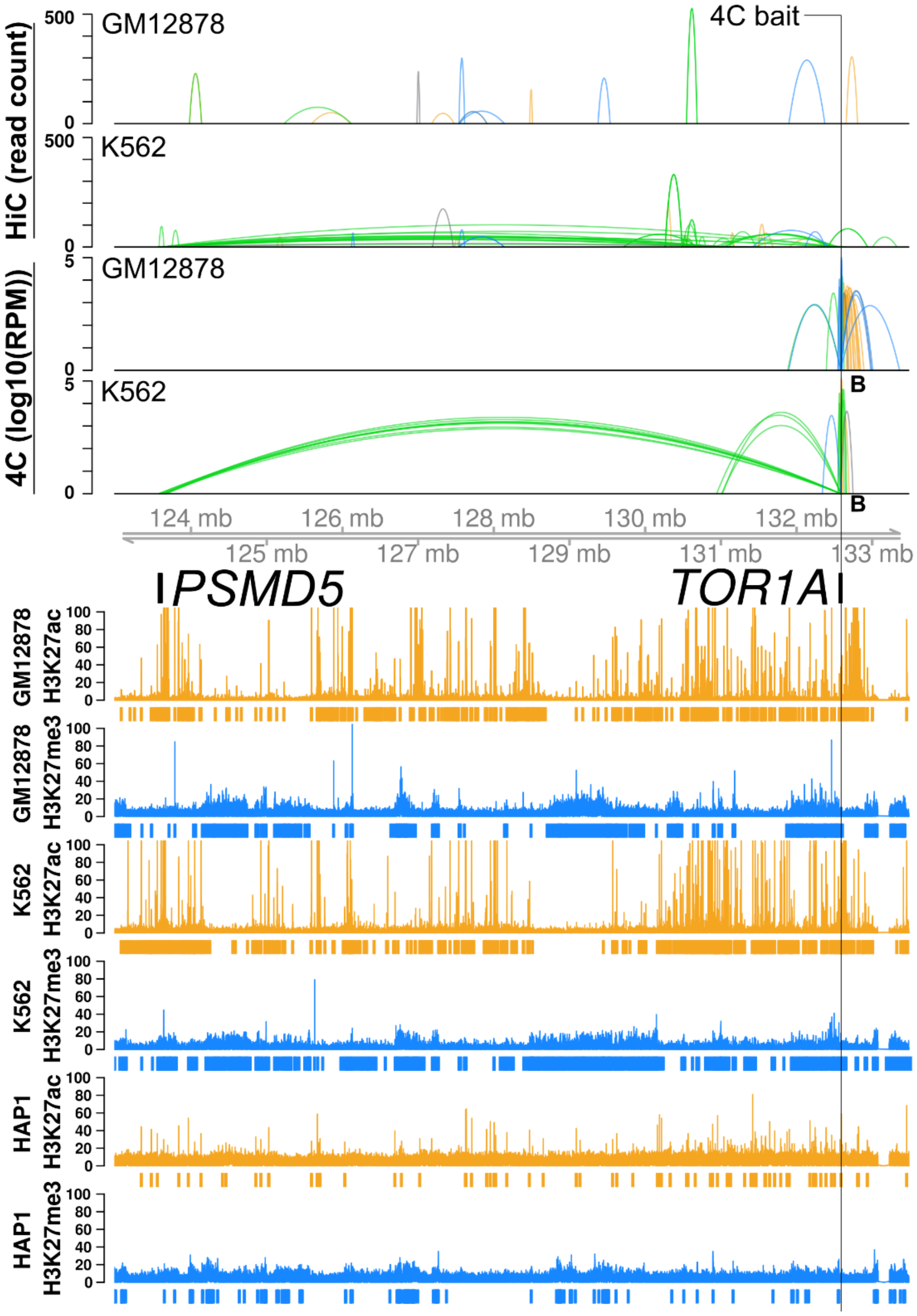

Number of different states of 4C interactions:

	A	B	R	Q
**GM12878**	12	3	8	2
**K562**	3	16	1	1

Supplementary Figure 2. **r.** Validation of Hi-C interactions using *TOR1A* as 4C bait. Extensive 'B-B' interactions (green arcs) connecting *TOR1A* gene are shown in Hi-C and validated by 4C in K562 cells. The colors of Hi-C and 4C interactions are based on the distal interacting regions to the gene's promoter and the 4C bait, respectively.

Blue: repressive; orange: active; green: both; grey: quiescent. The numbers of different states of 4C interactions were given in table.

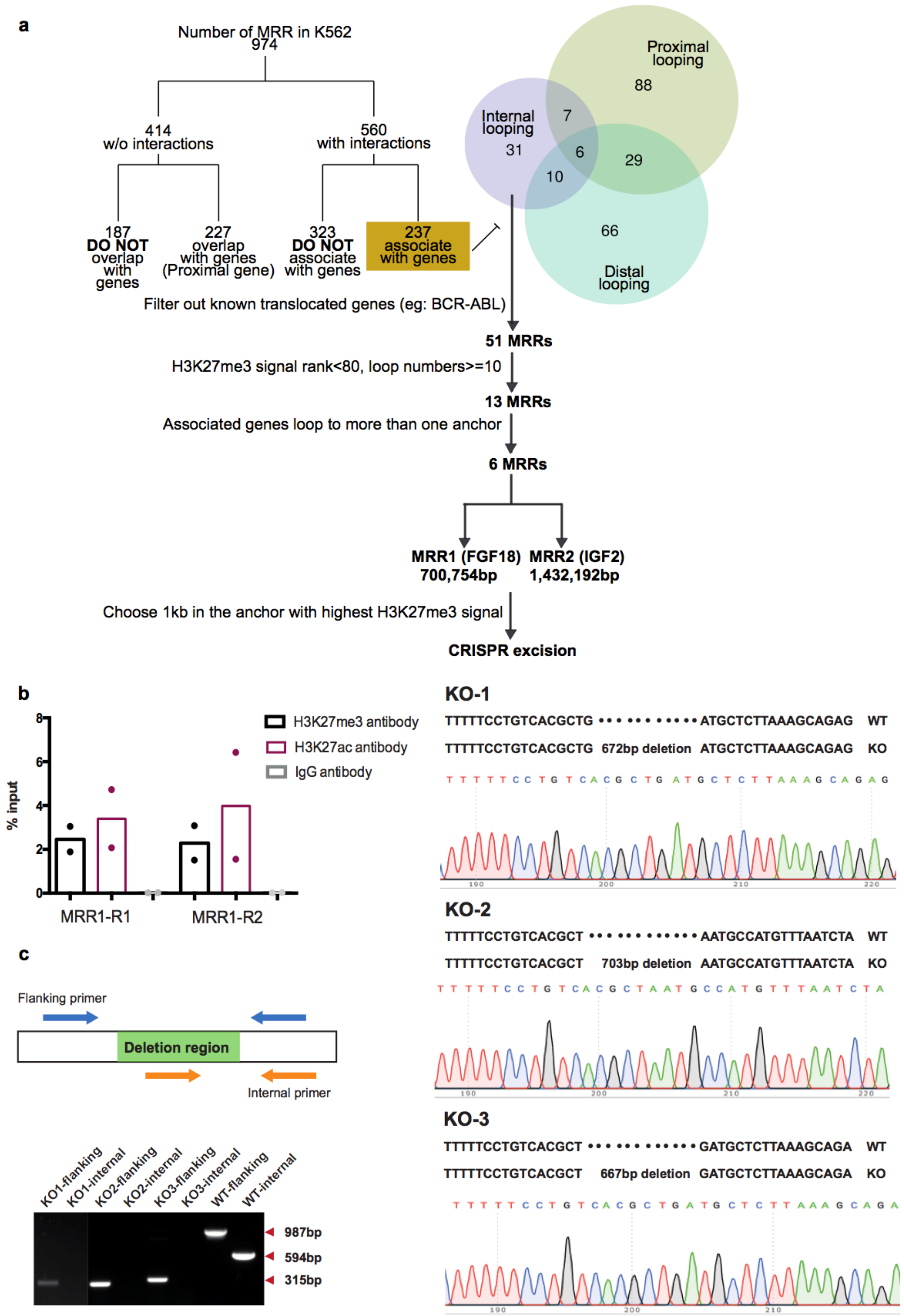

Supplementary Figure 3. CRISPR candidate selection process and MRR1-A1 knock out leads to *FGF18* upregulation. **a**. Selection process of H3K27me3-rich regions (MRRs) candidates to perform CRISPR. **b**. ChIP-qPCR of the MRR1 in wild-type K562 cells using H3K27me3 antibody, H3K27ac antibody and IgG antibody. Two regions

(MRR1-R1 and MRR1-R2) within MRR1 were tested (N=2). The y axis indicates as percentage of total input. The data shows average + standard error. **c**. Genotyping results of three MRR1-A1 knockout clones (KO-1, KO-2 and KO-3). Genotyping is performed using flanking primers and internal primers along the deletion region and results are shown by agarose gel electrophoresis and Sanger sequencing. This genotyping is repeated 3 times. Raw gel images are provided in Source Data file.

d

MRR1-A1 looping genes	Fold change in KO
RANBP17	-0.087
NPM1	-0.137
FGF18	0.4
FBXW11	-0.417
STK10	0.079
UBTD2	2.5

e

Nearby genes	Fold change in KO
SH3PXD2B	1.132
NEURL1B	0.158
DUSP1	0.46
GABRP	0.122
KCNMB1	-0.098
LCP2	0.21
C5orf58	5.8
DOCK2	-3.53

Supplementary Figure 3. d & e. Table of MRR1-A1 looping genes and proximal genes. The fold changes in RNA-seq of KO clones of the looping and proximal genes are shown.

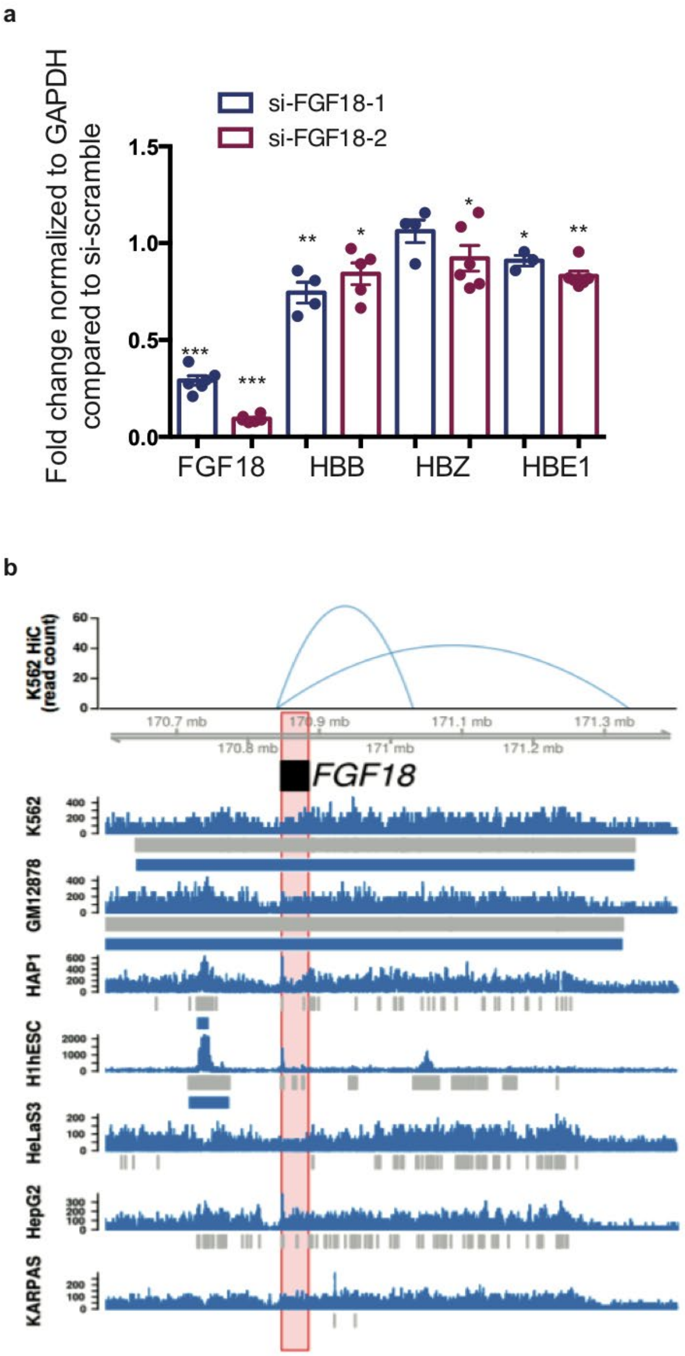

Supplementary Figure 4. MRR1-A1 KO leads to erythroid differentiation. **a**. Hemoglobin genes (*HBB*, *HBZ* and *HBE1*) expression decreased upon siRNA knocking down *FGF18* gene (N=5). **b.** MRR calling at *FGF18* region in seven cell lines

(K562, GM12878, HAP1, H1hESC, HeLaS3, HepG2 and KARPAS). MRRs are indicated by blue bars and the *FGF18* gene region is highlighted by the red box. All data shown here are average + standard error. P value is calculated by two-tailed student's t-test. P value less than 0.05 is shown as *. P value less than 0.01 is shown as **. P value less than 0.001 is shown as ***.

a

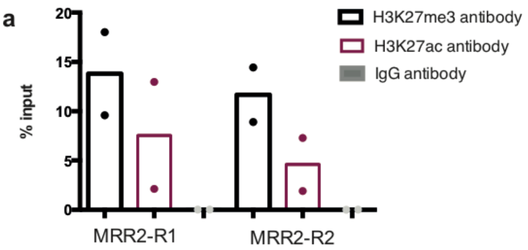

b

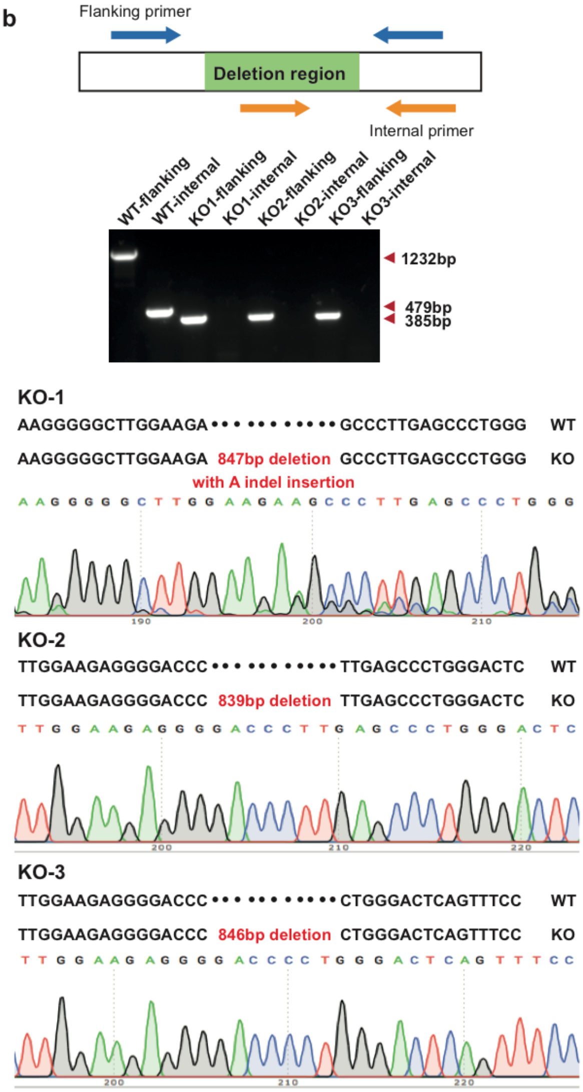

c

MRR2-A1 looping genes	Fold change in S1KO
TOLLIP	0.204
TOLLIP-AS1	-0.018
IFITM10	-1.17
LSP1	1.56
MRPL23	0.126
ASCL2	2.46
TSSC4	0.34
KCNQ1OT1	-0.425
SLC22A18	-1.27

d

Nearby genes	Fold change in KO
MUC2	2.5
MUC5B	0.298
MOB2	0.337
KRTAP5-AS1	0.167
CTSD	0.301
H19	4.010
C11ORF21	1.64
TSPAN32	0.43
CD81	0.0376
CDKN1C	-1.53
PHLDA2	-0.32
SNORA54	-0.30
NAP1L4	-0.08
CARS	-0.06

e

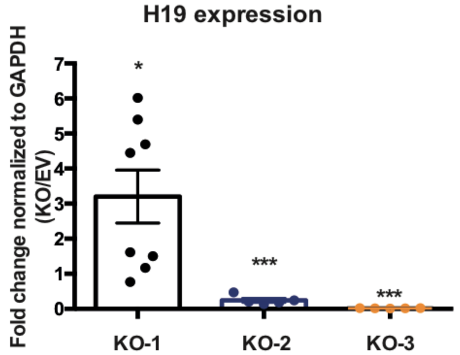

Supplementary Figure 5. MRR2-A1 knock out leads to upregulation of multiple genes including *IGF2* gene. **a**. ChIP-qPCR of the MRR2 in wild-type K562 cells using H3K27me3 antibody, H3K27ac antibody and IgG antibody. Two regions (MRR2-R1 and MRR2-R2) within MRR2 were tested (N=2). The y axis indicates as percentage of

total input. The data shows average + standard error. **b**. Genotyping results of three MRR2-A1 knockout clones (KO-1, KO-2 and KO-3). Genotyping is performed using flanking primers and internal primers along the deletion region and results are shown by gel electrophoresis and Sanger sequencing. Genotyping is repeated three times. **c & d.** Table of MRR2-A1 looping genes and proximal genes, and the fold changes in the RNA-seq of KO clones. **e.** RT-qPCR of *H19* gene shown in empty vector cells (EV) and three MRR2-C1 knock out cells (KO-C1, KO-C2 and KO-C3). N=5 for each clone. The data shows average + standard error. P value is calculated by two-tailed student's t-test. P value less than 0.05 is shown as *. P value less than 0.001 is shown as ***. Raw gel images are provided in Source Data file.

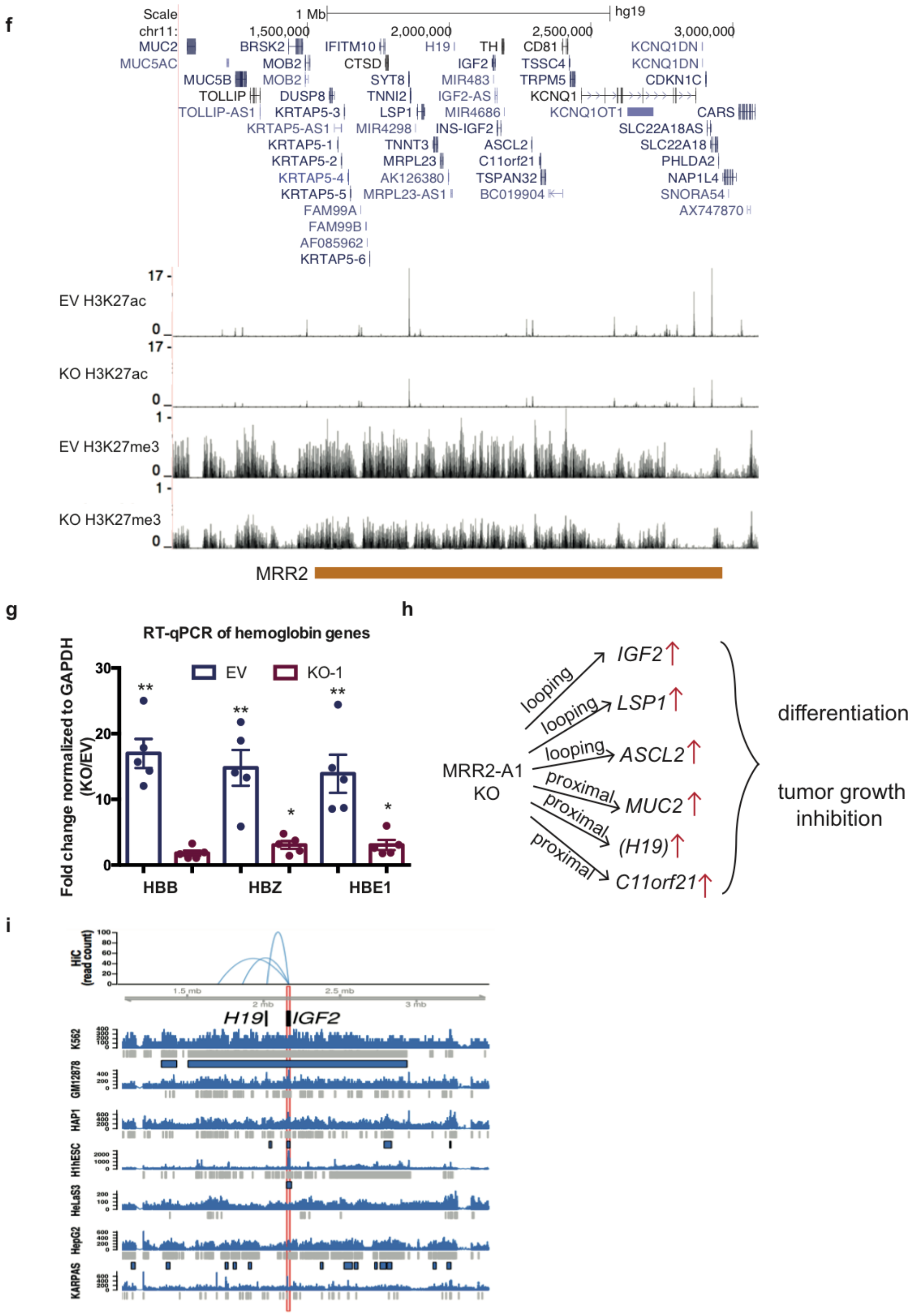

Supplementary Figure 5. f. Screenshot of MRR2 nearby regions with ChIP-seq of H3K27ac and H3K27me3 in EV and KO clones. MRR2 was indicated by yellow bar. **g.** Hemoglobin genes (*HBB*, *HBZ* and *HBE1*) expression decreased upon siRNA knockdown of *IGF2* gene (N=5). **h.** Model of MRR2-A1 knockout which leads to gene

upregulation of multiple looping and proximal genes. Collectively, these changing gene expression levels lead to cell differentiation and tumor growth inhibition. **i.** MRR calling at *IGF2* region in seven cell lines (K562, GM12878, HAP1, H1hESC, HeLaS3, HepG2 and KARPAS). MRRs are indicated by as blue bars and *IGF2* gene region is highlighted by the red box. All data shown here are average + standard error. P value is calculated by two-tailed student's t-test. P value less than 0.05 is shown as *. P value less than 0.01 is shown as **.

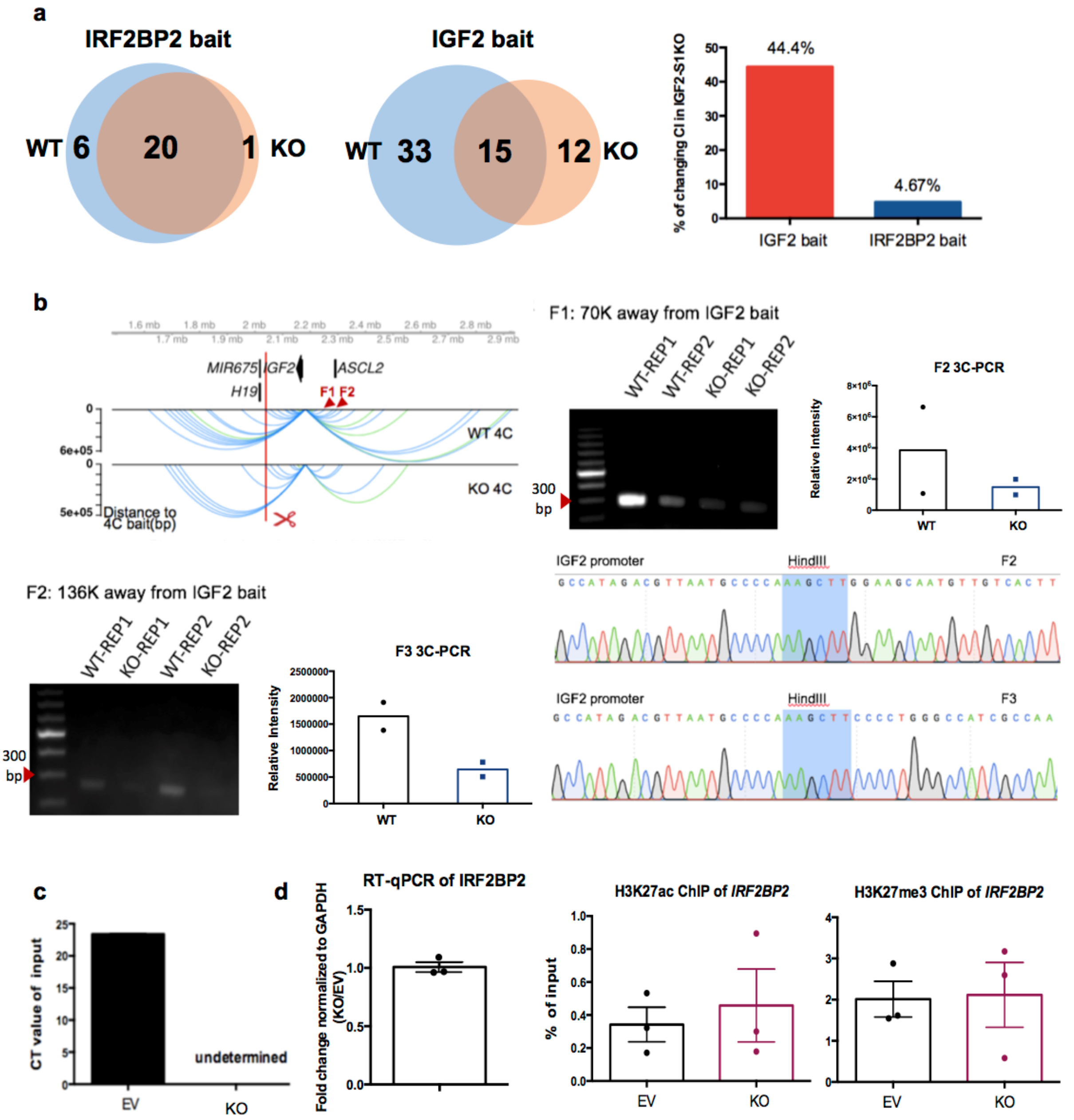

Supplementary Figure 6. Chromatin interactions changes upon MRR2-A1 KO were confirmed by 3C-PCR. **a.** Chromatin interactions changed at *IGF2* bait compared to *IRF2BP2* (control) bait after silencer KO. The Y-axis shows the percentage of

changing loops in KO clones**. b.** The losses of two different loops (F1 and F2) were validated by 3C-PCR followed by Sanger sequencing. Two different 3C libraries were used here. 3C-PCR was repeated by three times **c**. ChIP-qPCR at *IGF2* CRISPR excised region showed this region was completely deleted in the total input. **d**. *IRF2BP2* gene (control used in 4C-seq) had similar expression in EV and KO (N=3). ChIP-qPCR of H3K27me3 and H3K27ac for the *IRF2BP2* gene did not change after KO (N=3). Data shown here are average + standard error. Raw gel images are provided in Source Data file.

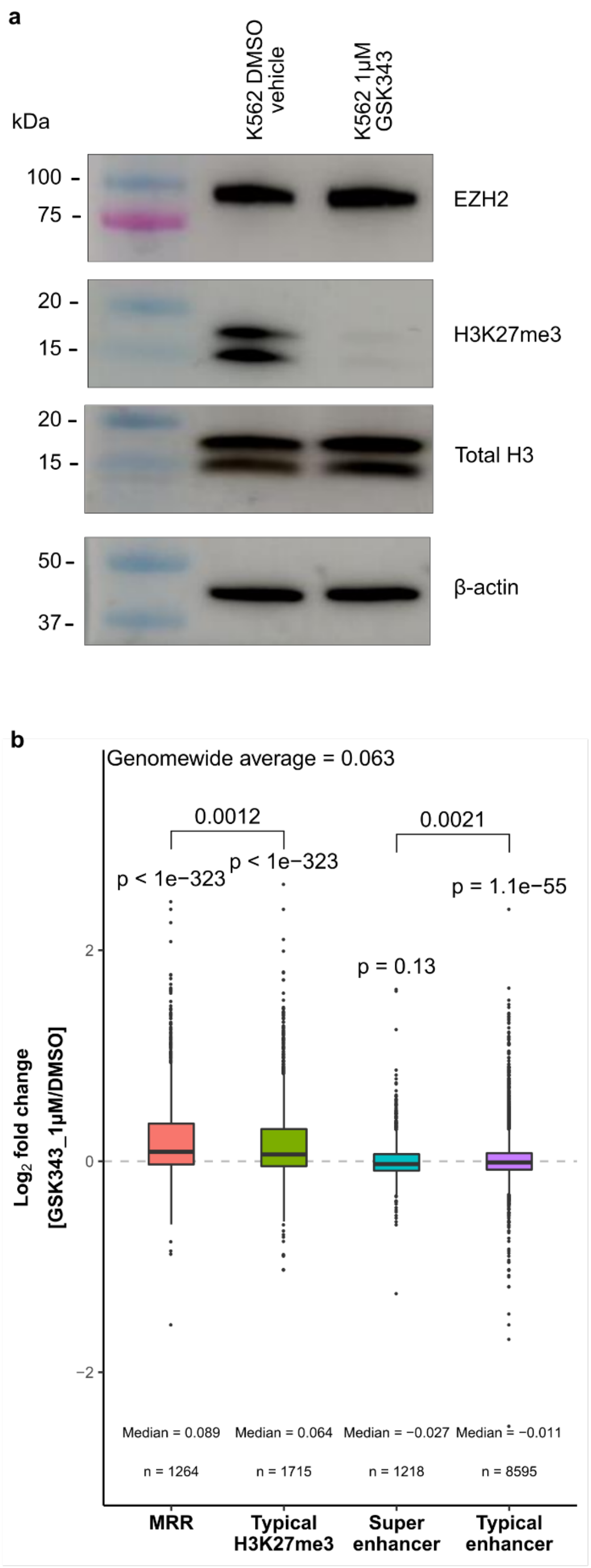

Supplementary Figure 7. Histone modifications, gene expression, and chromatin interactions changes upon EZH2 inhibition. **a** Western blot of EZH2 and H3K27me3 in DMSO and 1μM GSK343-treated K562 cells. The western blot was repeated for 3 times with similar results. **b.** TPM changes of genes associated with different types of

peaks in 1μM GSK343-treated K562 cells. Genes included: 1) Genes overlapped with different peaks as it is normally considered; 2) Genes associated with different peaks through Hi-C interaction. Box and whiskers plot: whiskers were extended to the furthest value that is no more than 1.5 times the inter-quartile range. The boxes represent the 25th percentile, median, and 75th percentile. Wilcoxon test p value, ns: $p > 0.05$, *: $p <= 0.05$, **: $p <= 0.01$, ***: $p <= 0.001$, ****: $p <= 0.0001$. Raw gel images are provided in Source Data file.

c

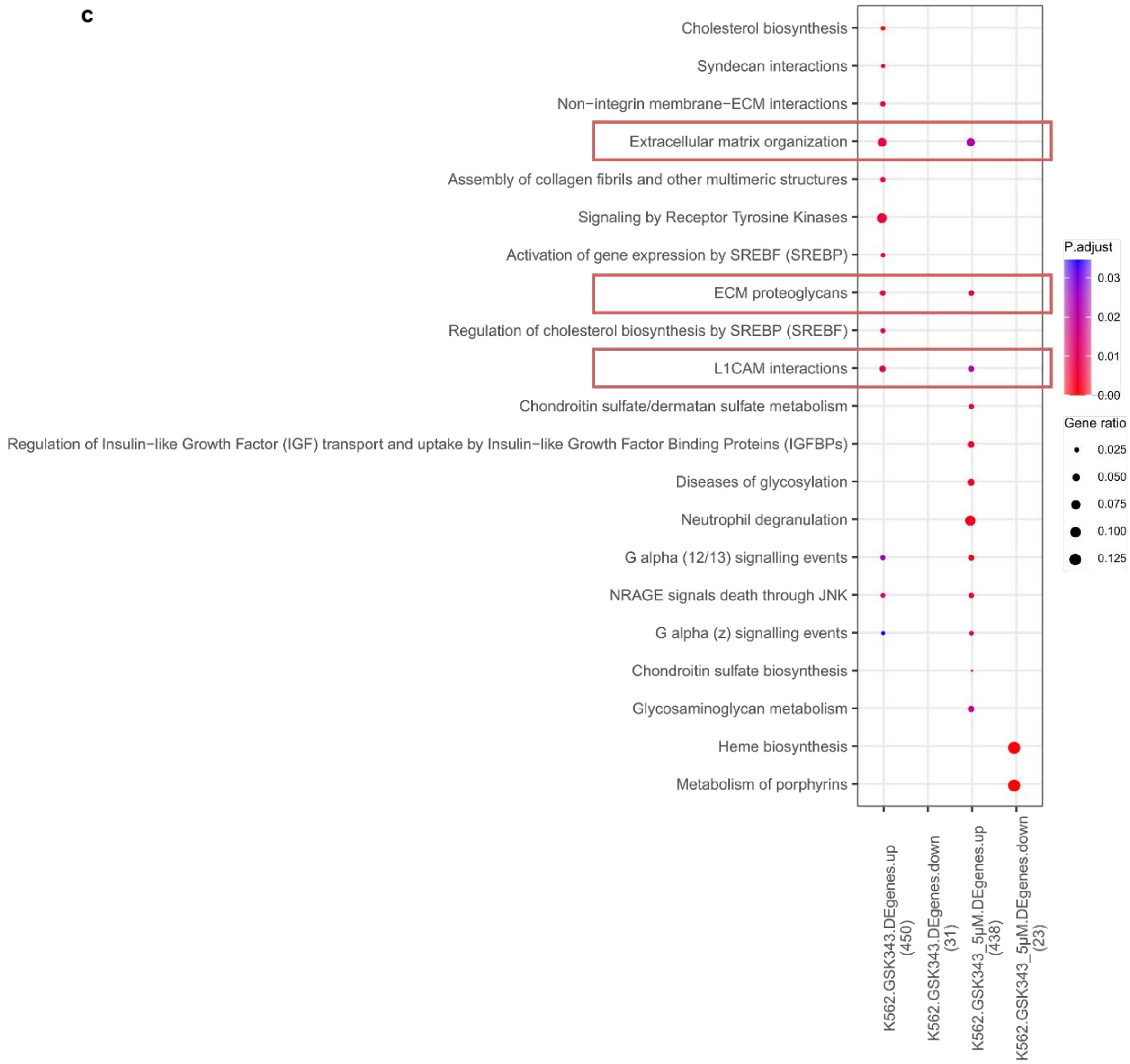

Supplementary Figure 7. c. REACTOME pathway enrichment analysis of differentially expressed genes in 1μM GSK343-treated K562 and 5μM GSK343-treated K562. Up and down stand for the direction of expression changes.

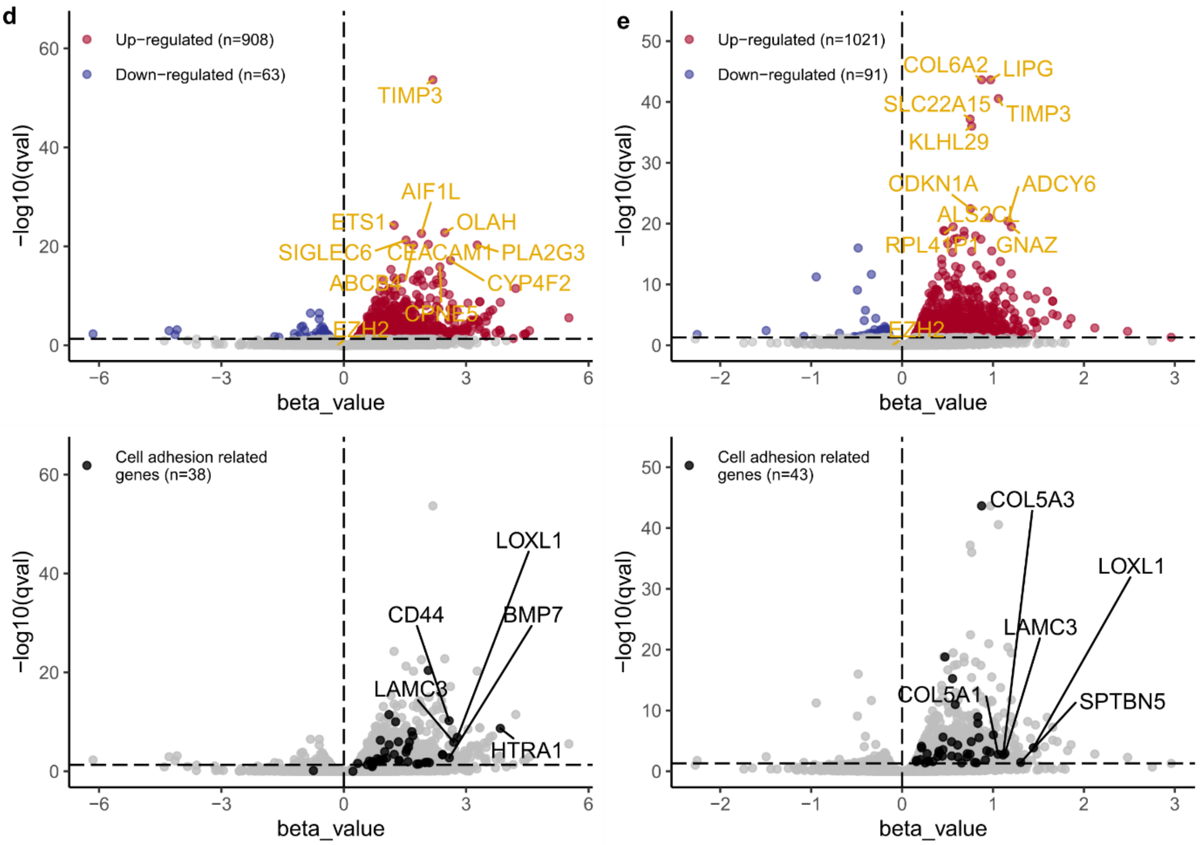

Supplementary Figure 7. **d & e.** Volcano plot of differentially expressed genes in 1μM GSK343-treated K562 and 5μM GSK343-treated K562. In each subfigure, top panels are: Top 10 most significant DE genes. Significance threshold of q value < 0.05 is used. Beta value is an estimator of fold change calculated by sleuth in natural log;

bottom panels are: volcano plot with cell-adhesion-related genes labeled. These genes are under REACTOME pathways of “Extracellular matrix organization”, “Non-integrin membrane-ECM interactions”, “Non-integrin membrane-ECM interactions”, “L1CAM interactions”, and “ECM proteoglycans”.

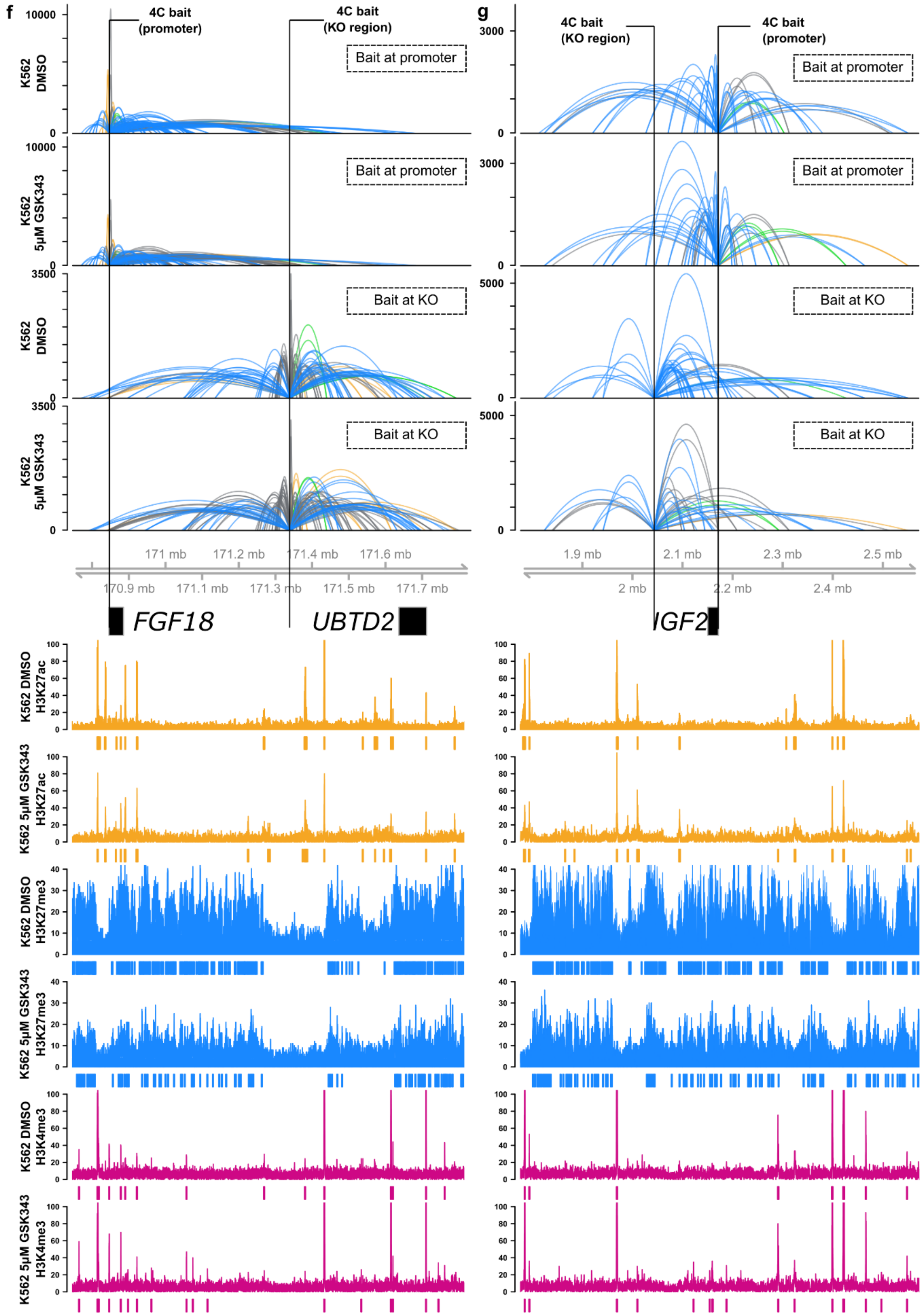

Supplementary Figure 7. f & g. 4C results using the bait of *FGF18*, *FGF18* KO region (MRR1-A1), *IGF2*, and *IGF2* KO region (MRR2-A1) in DMSO and 5µM GSK343-treated cells. The baits of 4C baits were indicated by vertical lines. The baits of each 4C panel were indicated by textboxes. For the baits at the gene promoters, the 4C

baits were designed at around gene promoter. For the bait at knockouts (KOs), the 4C baits were designed at the CRISPR KO regions (in Figure 3 & Figure 5). The colors of 4C interactions are based on the distal interacting regions to the 4C bait. Blue: repressive; orange: active; green: both; grey: quiescent. Height of 4C is in RPM. ChIP-seq signal and ChIP-seq peaks of H3K27ac, H3K27me3, and H3K4me3 in DMSO and 5μM GSK343-treated K562 cells were given in the lower panels.

h

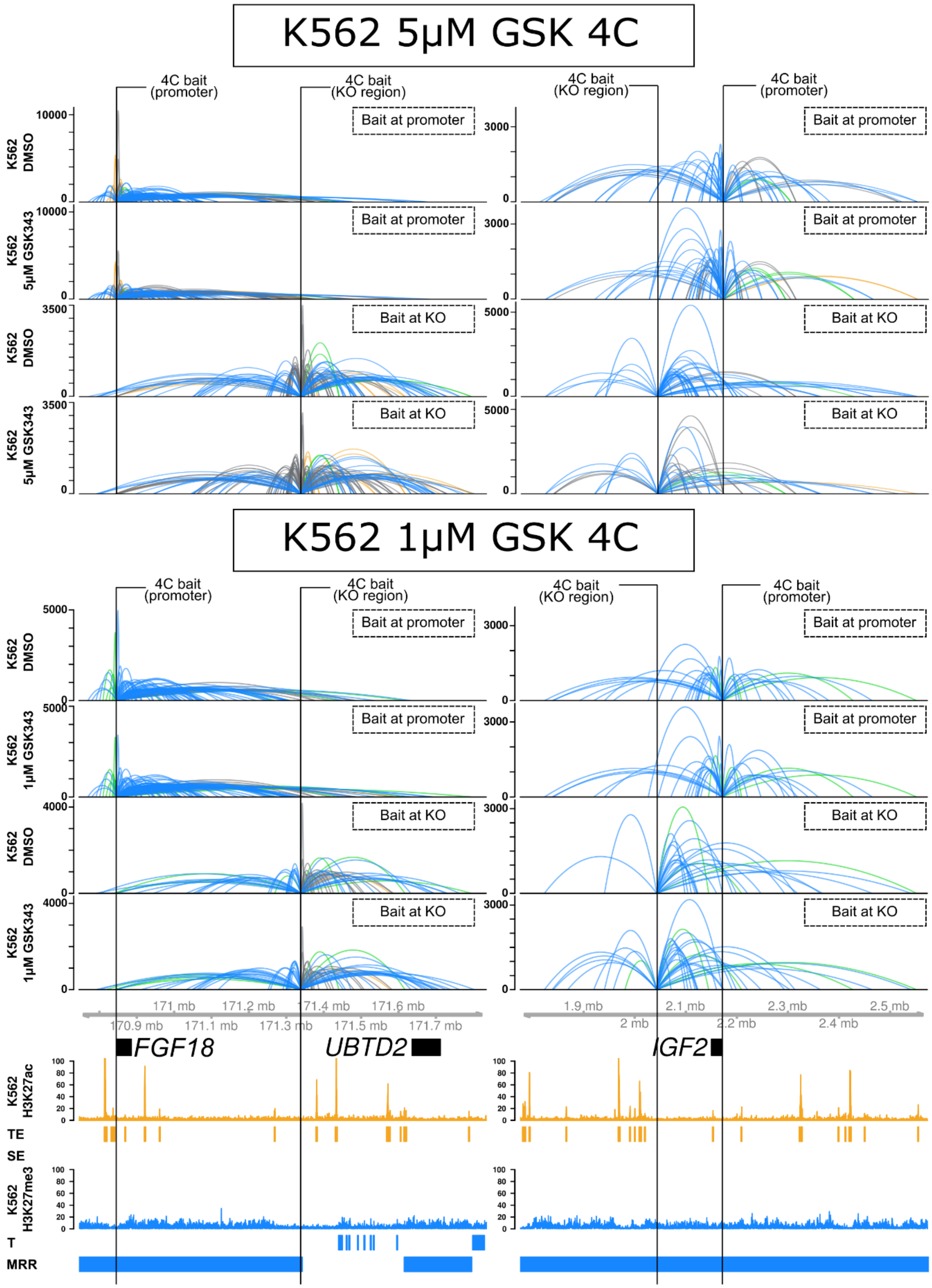

Supplementary Figure 7. **h.** Comparison of 4C results in 5μM GSK343 and 1μM GSK343 treatment using baits of *FGF18*, *FGF18* KO region (MRR1-A1), *IGF2*, and *IGF2* KO region (MRR2-A1). ChIP-seq signal and ChIP-seq peaks of H3K27ac, H3K27me3, and H3K4me3 in WT K562 cells were given in the lower panels. Details

of the representation of 4C and ChIP-seq are the same as in **Supplementary Figure 7f-7g**.

i

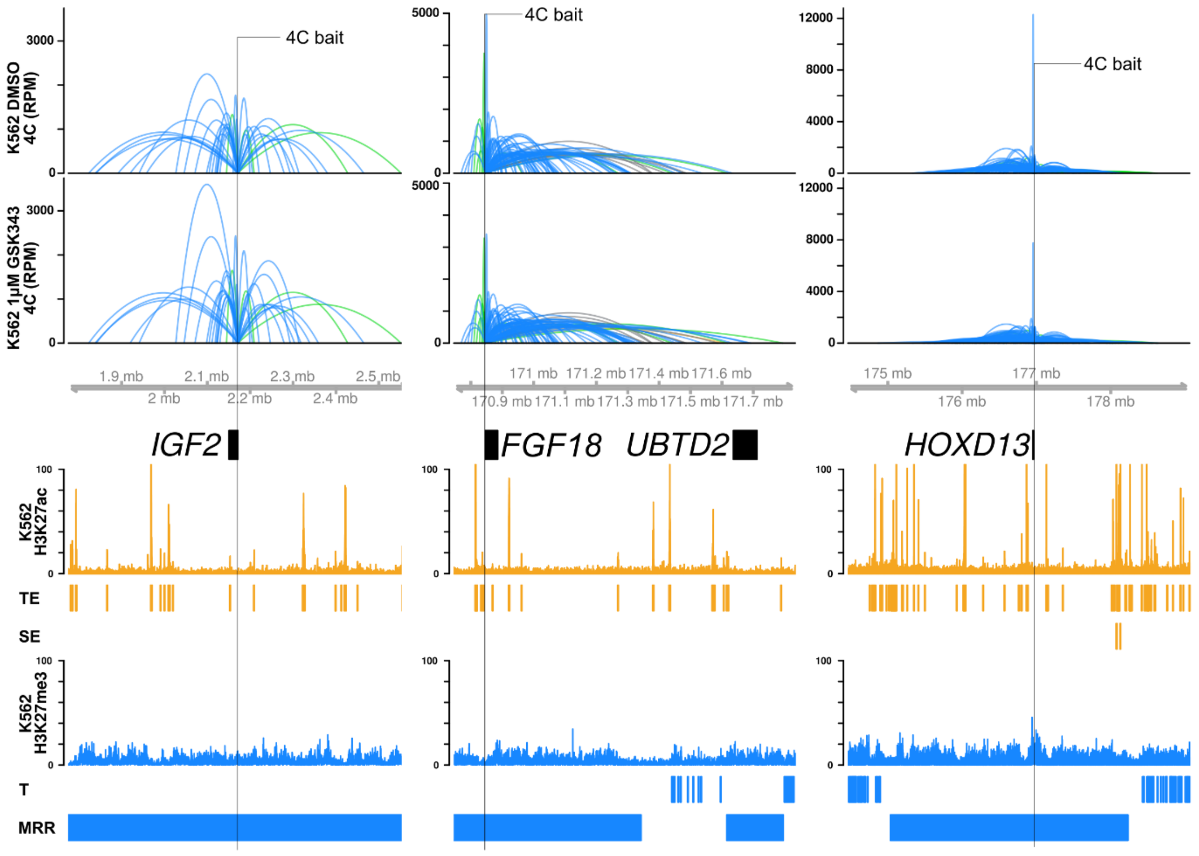

Supplementary Figure 7. **i.** 4C results of *IGF2*, *FGF18* and *HOXD13* in K562 DMSO and 1μM GSK343 treated cells. ChIP-seq signal and ChIP-seq peaks of K562 cells were given in the lower panels. ChIP-seq signal and ChIP-seq peaks of H3K27ac, H3K27me3, and H3K4me3 in WT K562 cells were given in the lower panels. Details

of the representation of 4C and ChIP-seq are the same as in **Supplementary Figure 7f-7g**.

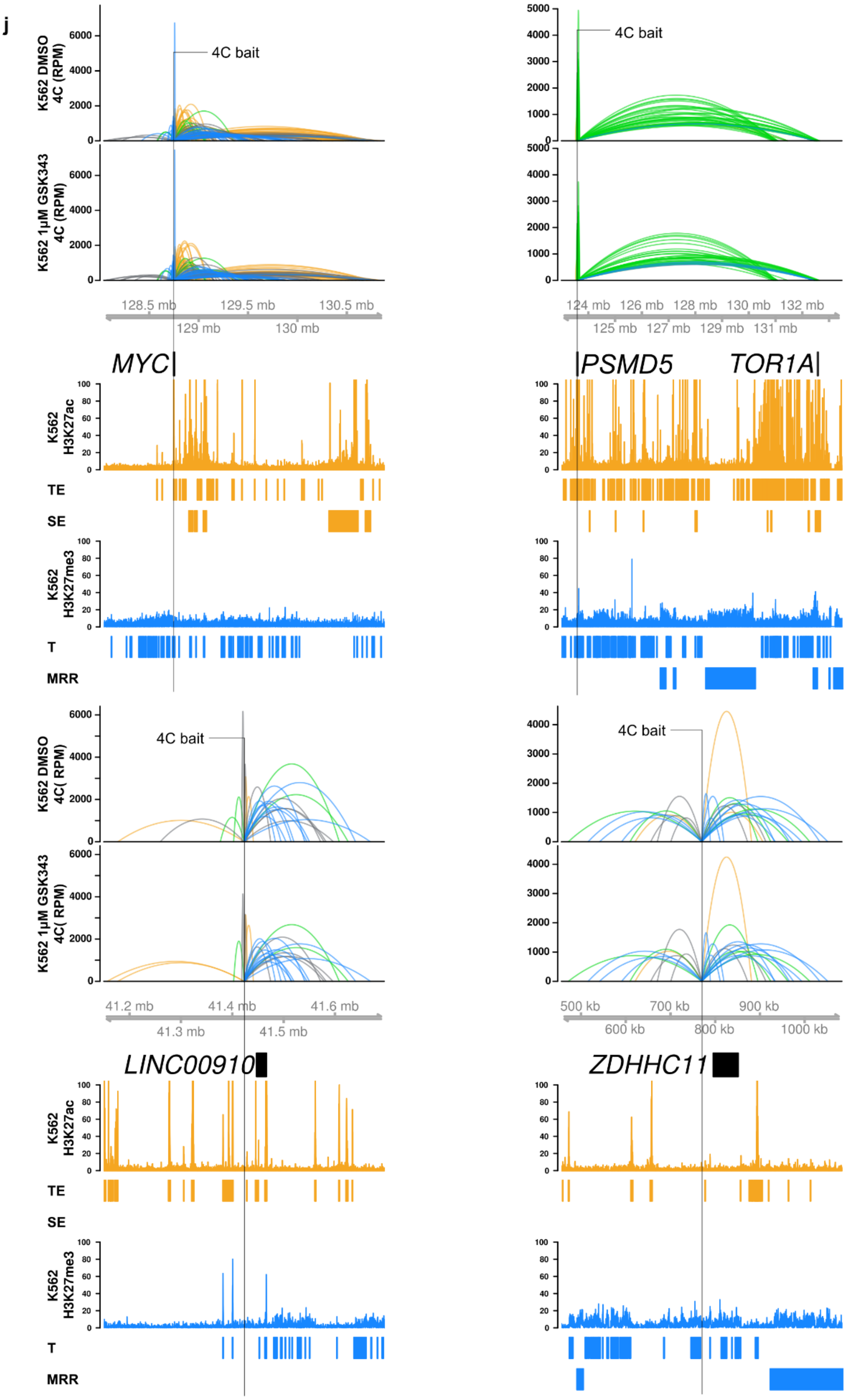

Supplementary Figure 7. **j.** 4C results of *MYC*, *PSMD5*, *LINC00910* and *ZDHHC11* genes in DMSO and 1μM GSK343 treated K562 cells. ChIP-seq signal and ChIP-seq peaks of H3K27ac, H3K27me3, and H3K4me3 in WT K562 cells were given in the

lower panels. Details of the representation of 4C and ChIP-seq are the same as in **Supplementary Figure 7f-7g**.

a

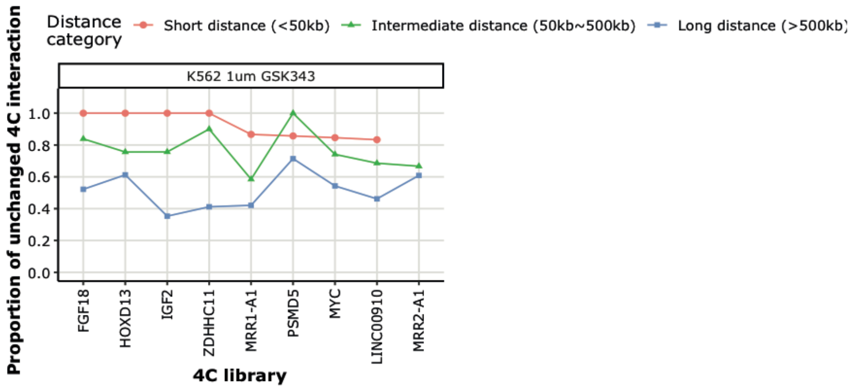

b

RPMs of 4C chromatin interactions

bait	hit	hit coordinates	RPM DMSO-rep1	RPM GSK343-rep1	RPM DMSO-rep2	RPM GSK343-rep2
IGF2 gene	MRR2-A1	Chr11: 2023184-2029380	1520	2814	1604	3632
MRR1-A1	*FGF18* gene	Chr5: 170840936-170842644	718	719	612	587
FGF18 gene	MRR1-A1	Chr5: 171344255-171347257	883	1209	704	651

c

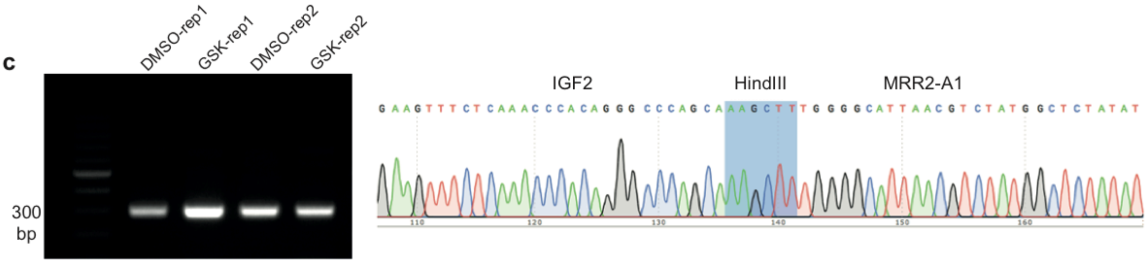

d

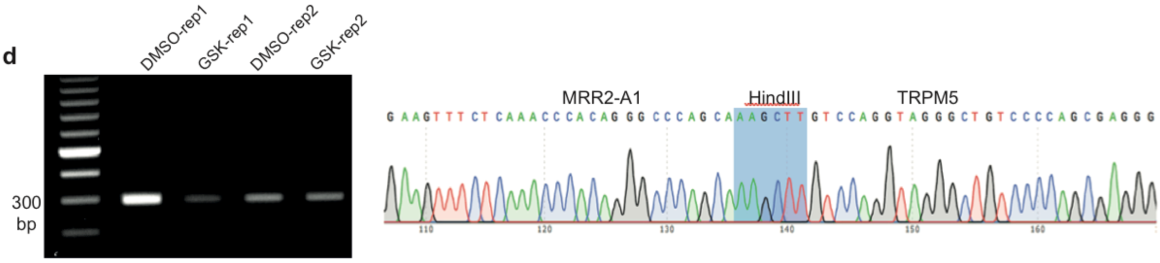

Supplementary Figure 8. Chromatin interactions changes upon GSK343 treatment were confirmed by 3C-PCR. **a.** Proportion of unchanged 4C interactions in different distance categories in 1μM GSK343-treated K562 cells. As the distance of 4C interactions increases, the proportion of unchanged 4C interactions drops, indicating

that long-range interactions are perturbed. **b.** Table of RPMs of 4C chromatin interactions in two different replicates in DMSO and GSK343 condition. **c.** 3C-PCR gel image and Sanger sequencing of the *IGF2*-MRR2-A1 loop in DMSO and GSK343-treated cells (two replicates). **d.** 3C-PCR gel image and sanger sequencing of *TRPM5*-MRR2-A1 loop in DMSO and GSK343-treated cells (two replicates).

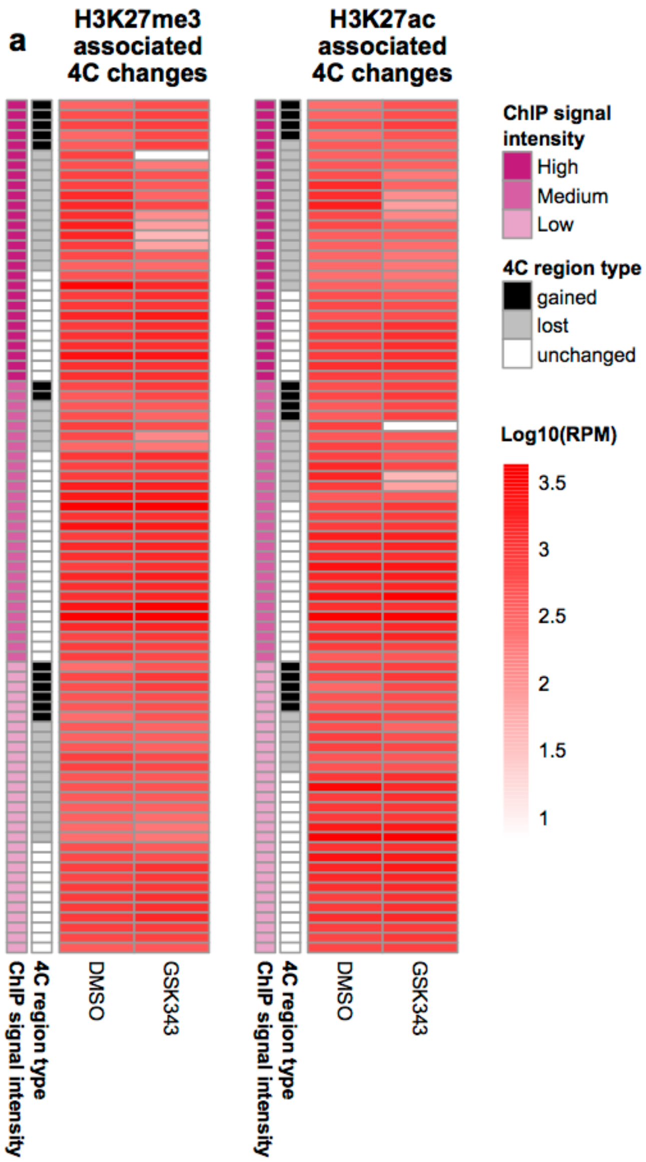

Supplementary Figure 9. Integrative analysis of H3K27me3, H3K27ac and chromatin interactions upon EZH2 inhibition. **a.** 4C interaction intensity changes at 4C regions with different levels of H3K27me3/H3K27ac signal in DMSO-treated K562 cells. Left panel, different 4C regions are classified according to their H3K27me3 signal intensity in DMSO-treated K562 cells. H3K27me3 signal level at these 4C regions are tertiled in three cohorts: high, medium, and low. 4C regions type indicates different categories of 4C regions. Gained, 4C interactions present in GSK343-treated 4C but not DMSO-treated 4C; lost, 4C interaction present in GSK343-treated 4C but not DMSO-treated 4C; unchanged, 4C interactions present in both GSK343-treated and DMSO-treated 4C. The 4C interaction intensities are shown in $\log_{10}$ transformed RPM. Right panel, different 4C regions are classified according to their H3K27ac signal intensity in DMSO-treated K562 cells. Similar to the left panel, H3K27ac signal level at these 4C regions are tertiled in three cohorts.

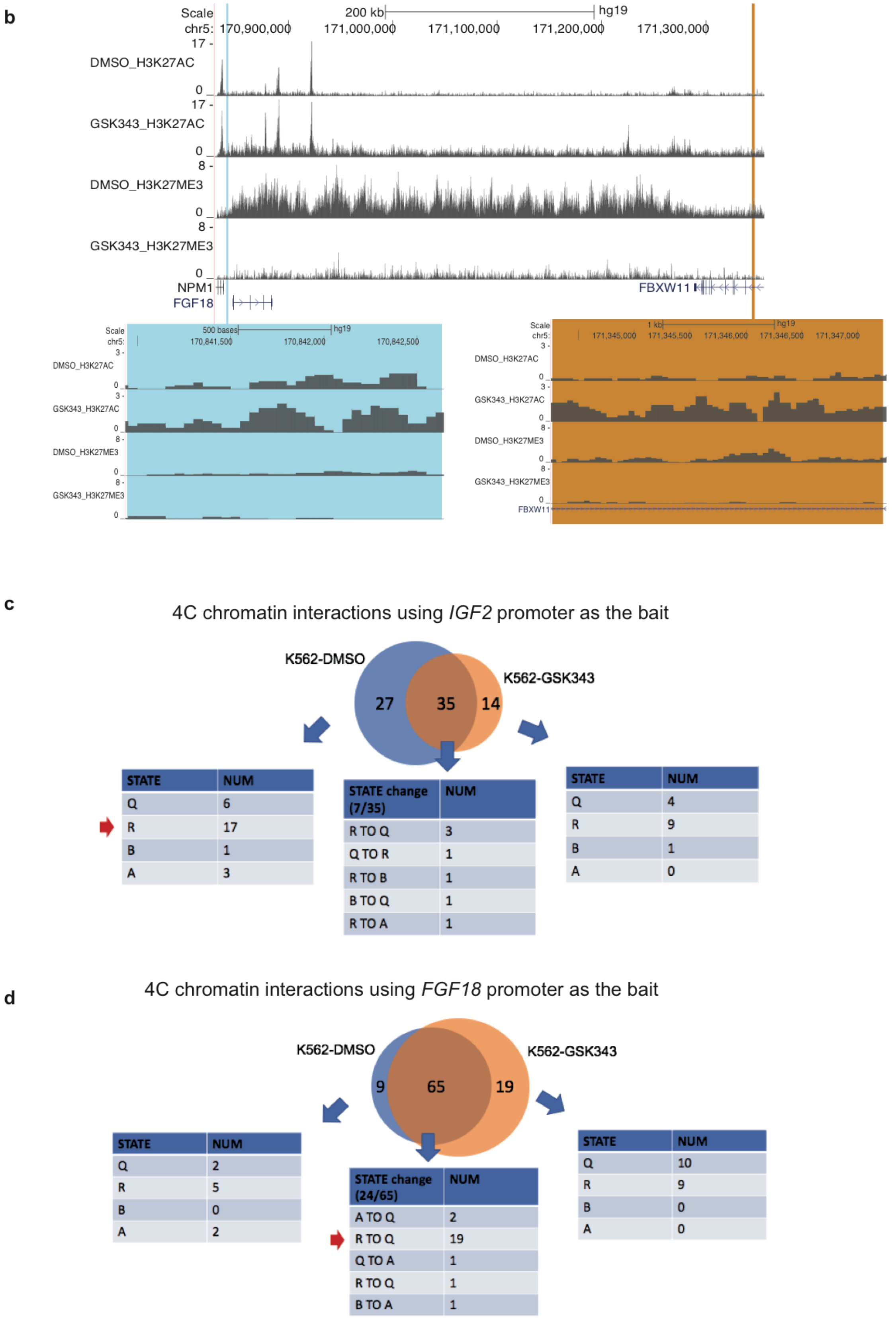

Supplementary Figure9. b. Screenshot of H3K27me3 and H3K27ac ChIP-seq in DMSO and GSK343 condition as well as zoomed-in view at *FGF18* and MRR1-A1 site. **c.** 4C chromatin interactions using *IGF2* promoter as the bait in DMSO and GSK343.

d. 4C chromatin interactions using *FGF18* promoter as the bait in DMSO and GSK343. The red error indicates the differences between *IGF2* and *FGF18*.

Supplementary References

1 Pimentel, H., Bray, N. L., Puente, S., Melsted, P. & Pachter, L. Differential analysis of RNA-seq incorporating quantification uncertainty. *Nat Methods* **14**, 687-690, doi:10.1038/nmeth.4324 (2017).

2 Kim, M. K., Lesoon-Wood, L. A., Weintraub, B. D. & Chung, J. H. A soluble transcription factor, Oct-1, is also found in the insoluble nuclear matrix and possesses silencing activity in its alanine-rich domain. *Mol Cell Biol* **16**, 4366-4377, doi:10.1128/mcb.16.8.4366 (1996).

3 Antalis, T. M., Costelloe, E., Muddiman, J., Ogbourne, S. & Donnan, K. Regulation of the plasminogen activator inhibitor type-2 gene in monocytes: localization of an upstream transcriptional silencer. *Blood* **88**, 3686-3697 (1996).

4 French, J. D. *et al.* Functional variants at the 11q13 risk locus for breast cancer regulate cyclin D1 expression through long-range enhancers. *Am J Hum Genet* **92**, 489-503, doi:10.1016/j.ajhg.2013.01.002 (2013).

5 Dong, J. M. & Lim, L. The human neuronal alpha 1-chimaerin gene contains a position-dependent negative regulatory element in the first exon. *Neurochem Res* **21**, 1023-1030, doi:10.1007/bf02532412 (1996).

6 Moffat, G. J., McLaren, A. W. & Wolf, C. R. Functional characterization of the transcription silencer element located within the human Pi class glutathione S-transferase promoter. *J Biol Chem* **271**, 20740-20747, doi:10.1074/jbc.271.34.20740 (1996).

7 Ye, J., Cippitelli, M., Dorman, L., Ortaldo, J. R. & Young, H. A. The nuclear factor YY1 suppresses the human gamma interferon promoter through two mechanisms: inhibition of AP1 binding and activation of a silencer element. *Mol Cell Biol* **16**, 4744-4753, doi:10.1128/mcb.16.9.4744 (1996).

8 Liu, J. & Francke, U. Identification of cis-regulatory elements for MECP2 expression. *Hum Mol Genet* **15**, 1769-1782, doi:10.1093/hmg/ddl099 (2006).

9 Schoch, S., Cibelli, G. & Thiel, G. Neuron-specific gene expression of synapsin I. Major role of a negative regulatory mechanism. *J Biol Chem* **271**, 3317-3323, doi:10.1074/jbc.271.6.3317 (1996).

10 Kheradmand Kia, S. *et al.* EZH2-dependent chromatin looping controls INK4a and INK4b, but not ARF, during human progenitor cell differentiation and cellular senescence. *Epigenetics Chromatin* **2**, 16, doi:10.1186/1756-8935-2-16 (2009).

11 Dietrich-Goetz, W., Kennedy, I. M., Levins, B., Stanley, M. A. & Clements, J. B. A cellular 65-kDa protein recognizes the negative regulatory element of human papillomavirus late mRNA. *Proc Natl Acad Sci U S A* **94**, 163-168, doi:10.1073/pnas.94.1.163 (1997).

12 Liu, B., Maul, R. S. & Kaetzel, D. M., Jr. Repression of platelet-derived growth factor A-chain gene transcription by an upstream silencer element. Participation by sequence-specific single-stranded DNA-binding proteins. *J Biol Chem* **271**, 26281-26290, doi:10.1074/jbc.271.42.26281 (1996).

13 Haniel, A., Welge-Lussen, U., Kuhn, K. & Poschl, E. Identification and characterization of a novel transcriptional silencer in the human collagen type IV gene COL4A2. *J Biol Chem* **270**, 11209-11215, doi:10.1074/jbc.270.19.11209 (1995).

14 Bossu, J. P. *et al.* Two regulatory elements of similar structure and placed in tandem account for the repressive activity of the first intron of the human apolipoprotein A-II gene. *Biochem J* **318 (Pt 2)**, 547-553, doi:10.1042/bj3180547 (1996).

15 Donda, A., Schulz, M., Burki, K., De Libero, G. & Uematsu, Y. Identification and characterization of a human CD4 silencer. *Eur J Immunol* **26**, 493-500, doi:10.1002/eji.1830260232 (1996).

16 Petrykowska, H. M., Vockley, C. M. & Elnitski, L. Detection and characterization of silencers and enhancer-blockers in the greater CFTR locus. *Genome Res* **18**, 1238-1246, doi:10.1101/gr.073817.107 (2008).

17 Qi, H., Liu, M., Emery, D. W. & Stamatoyannopoulos, G. Functional validation of a constitutive autonomous silencer element. *PLoS One* **10**, e0124588, doi:10.1371/journal.pone.0124588 (2015).

18 Zuccato, C. *et al.* Widespread disruption of repressor element-1 silencing transcription factor/neuron-restrictive silencer factor occupancy at its target genes in Huntington's disease. *J Neurosci* **27**, 6972-6983, doi:10.1523/JNEUROSCI.4278-06.2007 (2007).

19 Huang, D., Petrykowska, H. M., Miller, B. F., Elnitski, L. & Ovcharenko, I. Identification of human silencers by correlating cross-tissue epigenetic profiles and gene expression. *Genome Res* **29**, 657-667, doi:10.1101/gr.247007.118 (2019).

20 Doni Jayavelu, N., Jajodia, A., Mishra, A. & Hawkins, R. D. An atlas of silencer elements for the human and mouse genomes. *bioRxiv*, 252304, doi:10.1101/252304 (2018).

21 Pang, B. & Snyder, M. P. Systematic identification of silencers in human cells. *Nat Genet* **52**, 254-263, doi:10.1038/s41588-020-0578-5 (2020).
