# Supplementary material for: H3K27me3-rich genomic regions can function as silencers to repress gene expression via chromatin interactions
Source: Nat Commun. 2021 Jan 29;12:719. doi: 10.1038/s41467-021-20940-y (PMC7846766; doi:10.1038/s41467-021-20940-y)
Supplement: Supplementary file 2 — Description of Additional Supplementary Files [file 41467_2021_20940_MOESM2_ESM.pdf]

Description of Additional Supplementary Files

Supplementary Data 1. MRRs, typical H3K27me3 peaks, super-enhancers and typical H3K27ac peaks in K562 GM12878 and HAP1 (Excel Spreadsheet). This table lists all the coordinates and associated genes of the different categories of peaks. More details can be found in the “README” tab.

Supplementary Data 2. MRRs in other cell lines (Excel Spreadsheet). This table lists all the MRRs in other cell lines (H1hESC, HEPG2, HeLaS3, KARPAS-422, Pfeiffer, WSU-DLCL2) in addition to K562, GM12878 and HAP1. More details can be found in the “README” tab.

Supplementary Data 3. Transcription factor binding enrichment at interacting regions of MRRs (Excel Spreadsheet). This table lists the binding enrichment of different TFs (CTCF, EZH2, GATAD2B, RAD21, REST, SMC3, YY1, and ZNF143) at the interacting regions of MRRs. More details can be found in the “README” tab.

Supplementary Data 4. Statistical testing for change and unchanged 4C interactions of high or low H3K27me3 level. More details can be found in the “README” tab.

Supplementary Data 5. ChIP-seq peaks in K562 cells with EZH2 inhibition and HAP1 WT cells (Excel Spreadsheet). This table lists all the ChIP-seq peaks of 5μM GSK343-treated K562 cells that we sequence in this paper (related to Figure 8). More details can be found in the “README” tab.

Supplementary Data 6. Differentially expressed genes in RNA-seq of K562 cells after EZH2 inhibition or CRISPR KO (Excel Spreadsheet). This table lists all the differentially expressed genes from the RNA-seq that we performed for this paper (related to Figure 8). More details can be found in the “README” tab. Sleuth (0.29.0) package[1] was used to analysis differentially expressed genes. Wald test is used to derive significance of changes in gene expression.

Supplementary Data 7. Libraries Used (Excel Spreadsheet). This is a list of all the libraries used in this manuscript. More details can be found in the “README” tab.
