# Supplementary material for: H3K27me3-rich genomic regions can function as silencers to repress gene expression via chromatin interactions
Source: Nat Commun. 2021 Jan 29;12:719. doi: 10.1038/s41467-021-20940-y (PMC7846766; doi:10.1038/s41467-021-20940-y)
Supplement: Supplementary file 10 — Reporting Summary [file 41467_2021_20940_MOESM10_ESM.pdf]

nature research

Corresponding author(s): Melissa Jane Fullwood and Greg Tucker-Kellogg

Last updated by author(s): Dec 8, 2020

# Reporting Summary

Nature Research wishes to improve the reproducibility of the work that we publish. This form provides structure for consistency and transparency in reporting. For further information on Nature Research policies, see Authors & Referees and the Editorial Policy Checklist .

## Statistics

For all statistical analyses, confirm that the following items are present in the figure legend, table legend, main text, or Methods section.

| n/a | Confirmed | |
|---|---|---|
| ☐ | ☒ | The exact sample size (*n*) for each experimental group/condition, given as a discrete number and unit of measurement |
| ☒ | ☐ | A statement on whether measurements were taken from distinct samples or whether the same sample was measured repeatedly |
| ☐ | ☒ | The statistical test(s) used AND whether they are one- or two-sided<br>*Only common tests should be described solely by name; describe more complex techniques in the Methods section.* |
| ☒ | ☐ | A description of all covariates tested |
| ☒ | ☐ | A description of any assumptions or corrections, such as tests of normality and adjustment for multiple comparisons |
| ☐ | ☒ | A full description of the statistical parameters including central tendency (e.g. means) or other basic estimates (e.g. regression coefficient) AND variation (e.g. standard deviation) or associated estimates of uncertainty (e.g. confidence intervals) |
| ☐ | ☒ | For null hypothesis testing, the test statistic (e.g. *F*, *t*, *r*) with confidence intervals, effect sizes, degrees of freedom and *P* value noted<br>*Give P values as exact values whenever suitable.* |
| ☒ | ☐ | For Bayesian analysis, information on the choice of priors and Markov chain Monte Carlo settings |
| ☒ | ☐ | For hierarchical and complex designs, identification of the appropriate level for tests and full reporting of outcomes |
| ☒ | ☐ | Estimates of effect sizes (e.g. Cohen's *d*, Pearson's *r*), indicating how they were calculated |

*Our web collection on statistics for biologists contains articles on many of the points above.*

## Software and code

Policy information about availability of computer code

| | |
|---|---|
| Data collection | No software was used. |
| Data analysis | RNA-Seq, ChIP-Seq & 4Cdata analysis. For reads of RNA-seq and ChIP-seq, adptors are trimmed off by trimmomatic (0.38) with option 'TruSeq3-PE.fa:2:30:10 LEADING:3 TRAILING:3 SLIDINGWINDOW:4:15 MINLEN:36' and retained only those properly-paired reads after trimming. RNA-Seq reads of HAP1 EZH2KO/WT and K562 GSK343/DMSO were analysis with kallisto (0.44.0) with option '-b 100'. Differentially expressed genes were called using sleuth (0.29.0) with gene-level aggregation and wald test. ChIP-Seq reads of H3K2me3, H3K27ac and H3K4me3 were mapped by BOWTIE2 (v2.2.5) using default parameters in pair-end mode and filter out alignment with a mapq score smaller than 30. The two replicates were combined and peaks and bigWig files were generated by MACS2 (2.1.0.20150731) using option '-q 0.01' for H3K27ac and H3K4me3 and '--broad --broad-cutoff 0.1 -q 0.05' for H3K27me3. 4C reads were trimmed off HindIII digestion site using tagdust (2.33) and only those remained paired were mapped by BOWTIE2 (v2.2.5) with option '--end-to-end' in single-end mode. R3Cseq (1.24.0) was used to call significant interactions against Hind III digested genome background with a cut-off p value of 0.05. Significant interactions of two replicates were pooled and only one of the duplicated interacting regions was retained.<br>Definition of H3K27me3-rich regions(MRRs). H3K27me3 ChIP-Seq signal and peaks were obtained from ENCODE, and used as inputs of an in-house customized script (https://bitbucket.org/YichaoCai/rose_strict_share/src/master/<br>) that mimic the signal calculation of the ROSE (0.1) package. Missing H3K27me3 peaks from ENCODE were called using MACS2 (2.1.0.20150731)1with pooled replicates using option "--broad -q 0.05". First, ChIP-Seq peaks of H3K27me3 were stitched using a window size of 4 kb. After stitching, the treatment and control signal of the stitched peaks were calculated and used in the ranking in MRR calling. Super-enhancers were called in a similar manner except that a stitching window of 12.5 kb was used.<br>Hi-C and 4C interactions were drawn in arc style using Sushi (1.16.0) from Bioconductor. Tracks of ChIP-Seq signal and peaks were generated by Gviz (1.22.3).<br>Genomic feature enrichment analysis was performed using R package annotatr (1.8.0). Gene ontology, pathway enrichment analysis (REACTOME & KEGG) and map-view representation of enriched pathways were performed using R package clusterProfiler (3.10.1) and ReactomeRA (1.26.0). |

For manuscripts utilizing custom algorithms or software that are central to the research but not yet described in published literature, software must be made available to editors/reviewers. We strongly encourage code deposition in a community repository (e.g. GitHub). See the Nature Research guidelines for submitting code & software for further information.

## Data

Policy information about availability of data

All manuscripts must include a data availability statement. This statement should provide the following information, where applicable:

- Accession codes, unique identifiers, or web links for publicly available datasets
- A list of figures that have associated raw data
- A description of any restrictions on data availability

Novel sequencing data in this manuscript: GSE133183. This record has been made public.
Processed Hi-C interactions in K562, GM12878, and HAP1 were obtained from GEO (GSE63525 (https://www.ncbi.nlm.nih.gov/geo/query/acc.cgi?acc=GSE63525)). H3K27me3 and H3K27ac ChIP-Seq peaks in K562 and GM12878 obtained from ENCODE at UCSC (wgEncodeEH000031, wgEncodeEH000044, wgEncodeEH000030, wgEncodeEH000043). Other H3K27me3 ChIP-Seq data in H1hESC, HeLaS3, HepG2, and MCF7 are obtained ENCODE (ENCSR000ALU, ENCSR000APB, ENCSR000AOL, and ENCSR768LHG). H3K27me3 ChIP-Seq data in KARPAS-422, Pfeiffer, and WSU-DLCL2 were obtained from GEO (GSE40970 (https://www.ncbi.nlm.nih.gov/geo/query/acc.cgi?acc=GSE40970)). EZH2 ChIP-Seq data in K562, GM12878, H1hESC, HepG2, and HeLaS3 were obtained from ENCODE (ENCFF083IDB, ENCSR000ARD, ENCSR000ASY, ENCSR000ARI, and ENCSR000ATC).

# Field-specific reporting

Please select the one below that is the best fit for your research. If you are not sure, read the appropriate sections before making your selection.

☒ Life sciences ☐ Behavioural & social sciences ☐ Ecological, evolutionary & environmental sciences

For a reference copy of the document with all sections, see nature.com/documents/nr-reporting-summary-flat.pdf

# Life sciences study design

All studies must disclose on these points even when the disclosure is negative.

| | |
|---|---|
| Sample size | A minimum of 3 replicates was used for the Reverse Transcriptase-quantitative PCR and Western Blot data. The choice of 3 replicates is commonly used by studies in the field, for example, Kirschneck et al., 2017 (PMID: 29116140) and Espanola et al., 2020 (PMID: 33230303).<br>A minimum of 2 replicates was used for all of the sequencing-related experiments (4C, ChIP-Seq, RNA-Seq). The choice of 2 replicates is commonly used by studies in the field, for example, Kloetgen et al., 2020 (PMID: 32203470), Espanola et al., 2020 (PMID: 33230303), and Liu et al., 2020 (PMID: 33214551). For 3C-PCR, 2 replicates were performed and it is also used by studies in the field, for example, Kakui et al. (PMID: 28825727). |
| Data exclusions | No data was excluded |
| Replication | For the RNA-Seq, ChIP-Seq and Hi-C data that was used, we performed RT-qPCR, ChIP-qPCR and 4C respectively in order to verify the data. In addition, a minimum of 3 replicates was used for the Reverse Transcriptase-quantitative PCR and Western Blot data, and a minimum of 2 replicates was used for all of the sequencing-related experiments (4C, ChIP-Seq, RNA-Seq). The choice of replicates for each experiments follows the commonly used replicate numbers in studies in the field.<br>The figures showing 4C-seq, ChIP-seq, and RNA-seq contain the results from all replicates run. The figures for RT-qPCR and 3C-PCR show all replicates. The replicates are consistent with each other. For western blot, one representative replicate was shown in the figure, and the other replicates are shown in the Source Data Excel file. The other replicates are consistent with the representative replicate shown. |
| Randomization | The samples were prepared from experimental treatments where a cell line was treated with a drug or subjected to CRISPR. Covariates therefore were kept the same because the control cells came from the same cell line. |
| Blinding | Blinding was not possible here because we performed experimental treatments, and one investigator prepared, collected and analyzed the experimental treatments. |

# Reporting for specific materials, systems and methods

We require information from authors about some types of materials, experimental systems and methods used in many studies. Here, indicate whether each material, system or method listed is relevant to your study. If you are not sure if a list item applies to your research, read the appropriate section before selecting a response.

## Materials & experimental systems

| n/a | Involved in the study | |
|---|---|---|
| ☐ | ☒ | Antibodies |
| ☐ | ☒ | Eukaryotic cell lines |
| ☒ | ☐ | Palaeontology |
| ☐ | ☒ | Animals and other organisms |
| ☒ | ☐ | Human research participants |
| ☒ | ☐ | Clinical data |

## Methods

| n/a | Involved in the study | |
|---|---|---|
| ☐ | ☒ | ChIP-seq |
| ☒ | ☐ | Flow cytometry |
| ☒ | ☐ | MRI-based neuroimaging |

## Antibodies

Antibodies used

H3K27me3 (C36B11, Cell Signaling Technologies, catalog NO. 9733 and lot 8, 3.5ug antibody per IP)
H3K4me3 (#ab8580, Abcam)
H3K27ac (#ab4729, Abcam)
mouse IgG (#sc-2025, Santa Cruz)
EZH2 (Cell Signaling Technology AC22 #3147)
beta-Actin (abcam ab6276)
total H3 (abcam ab1791)
HRP-conjugated anti mouse secondary antibodies (Cell Signaling Technology #7076)
HRP-conjugated anti rabbit secondary antibodies (Cell Signaling Technology #7074)

Dilution protocol of the antibodies can be found in the Methods section.

Validation

H3K27me3 (C36B11, Cell Signaling Technologies #9733) (https://www.cellsignal.com/products/primary-antibodies/tri-methyl-histone-h3-lys27-c36b11-rabbit-mab/9733) Validation: Tri-Methyl-Histone H3 (Lys27) (C36B11) Rabbit mAb detects endogenous levels of histone H3 only when tri-methylated on Lys27. The antibody does not cross-react with non-methylated, mono-methylated or di-methylated Lys27. In addition, the antibody does not cross-react with mono-methylated, di-methylated or tri-methylated histone H3 at Lys4, Lys9, Lys36 or Histone H4 at Lys20.

H3K4me3 (#ab8580, Abcam) (https://www.abcam.com/histone-h3-tri-methyl-k4-antibody-chip-grade-ab8580.html) Validation: Chromatin was prepared from U-2 OS (Human bone osteosarcoma epithelial cell line) cells according to the Abcam X-ChIP protocol. Cells were fixed with formaldehyde for 10 minutes. The ChIP was performed with 25 µg of chromatin, 2 µg of ab8580 (blue), and 20 µl of Protein A/G sepharose beads. No antibody was added to the beads control (yellow). The immunoprecipitated DNA was quantified by real time PCR (Taqman approach). Primers and probes are located in the first kb of the transcribed region.

H3K27ac (#ab4729, Abcam) (https://www.abcam.com/histone-h3-acetyl-k27-antibody-chip-grade-ab4729.html?productWallTab=ShowAll#top-653) Validation: Chromatin was prepared from HeLa (Human epithelial cell line from cervix adenocarcinoma) cells according to the Abcam X-ChIP protocol. Cells were fixed with formaldehyde for 10 minutes. The ChIP was performed with 25 µg of chromatin, 2 µg of ab4729 (blue), and 20 µl of Protein A/G sepharose beads.No antibody was added to the beads control (yellow).The immunoprecipitated DNA was quantified by real time PCR (Taqman approach). Primers and probes are located in the first kb of the transcribed region.

mouse IgG (#sc-2025, Santa Cruz) (https://www.scbt.com/scbt/product/normal-mouse-igg?productCanUrl=normal-mouse-igg&_requestid=1078244) Validation: normal mouse IgG is an affinity purified, unconjuated conjugated isotype control immunoglobulinfrom mouse. Selected citation: Tan, M., et al. 2002. Phosphorylation on tyrosine- 15 of p34( Cdc2) by ErbB2 in hibits p34(Cdc2 ) activation and is involved in resistance to taxol-induced apoptosis. Mol. Cell 9: 993.

EZH2 (Cell Signaling Technology AC22 #3147) (https://www.cellsignal.com/products/primary-antibodies/ezh2-ac22-mouse-mab/3147) Validation: Ezh2 (AC22) Mouse mAb detects endogenous levels of total Ezh2 protein.
Species Reactivity: Human, Mouse, Rat, Monkey

beta-Actin (abcam ab6276) (https://www.abcam.com/beta-actin-antibody-ac-15-ab6276.html) Validation: Lane 1: Wild-type HAP1 cell lysate (20 µg)
Lane 2: Beta actin knockout HAP1 cell lysate (20 µg)
Lanes 1 and 2: Merged signal (red and green). Green - beta actin, ab6276 observed at 42 kDa. Red - loading control, ab181602 observed at 37 kDa.
Ab6276 was shown to specifically react with beta actin in wild-type HAP1 cells. No band was observed when beta actin knockout samples were used. Wild-type and beta actin knockout samples were subjected to SDS-PAGE. ab6276 (beta actin) and ab181602 (loading control to GAPDH) were diluted 1/5000 and 1/10 000 and incubated overnight at 4°C. Blots were developed with Goat anti-Mouse IgG H&L (IRDye® 800CW) preadsorbed (ab216772) and Goat Anti-Rabbit IgG H&L (IRDye® 680RD) preadsorbed (ab216777) secondary antibodies at 1/10 000 dilution for 1 h at room temperature before imaging.

total H3 (abcam ab1791) (https://www.abcam.com/histone-h3-antibody-nuclear-loading-control-and-chip-grade-ab1791.html)
Validation: All lanes : Anti-Histone H3 antibody - Nuclear Loading Control and ChIP Grade (ab1791) at 1/1000 dilution
Lane 1 : A431 (Human epithelial carcinoma cell line) Whole Cell Lysate
Lane 2 : Jurkat (Human T cell lymphoblast-like cell line) Whole Cell Lysate
Lane 3 : HEK293 (Human embryonic kidney cell line) Whole Cell Lysate
Lane 4 : A431 (Human epithelial carcinoma cell line) Whole Cell Lysate with Human Histone H3 peptide (ab12149) at 1 µg/ml
Lane 5 : Jurkat (Human T cell lymphoblast-like cell line) Whole Cell Lysate with Human Histone H3 peptide (ab12149) at 1 µg/ml
Lane 6 : HEK293 (Human embryonic kidney cell line) Whole Cell Lysate with Human Histone H3 peptide (ab12149) at 1 µg/ml
Lysates/proteins at 20 µg per lane.
Secondary
All lanes : Goat Anti-Rabbit IgG H&L (HRP) (ab6721) at 1/5000 dilution
Developed using the ECL technique.
Performed under reducing conditions.

Predicted band size: 15 kDa
Observed band size: 17 kDa

## Eukaryotic cell lines

Policy information about cell lines

| | |
|---|---|
| Cell line source(s) | K562, GM12878: ATCC; HAP1: Horizon Discovery |
| Authentication | None of the cell lines used were authenticated |
| Mycoplasma contamination | No mycoplasma testing was done |
| Commonly misidentified lines (See ICLAC register) | None |

## Animals and other organisms

Policy information about studies involving animals; ARRIVE guidelines recommended for reporting animal research

| | |
|---|---|
| Laboratory animals | Female CB17 SCID mice (6-8 weeks old) were used in this study. Mice were purchased from InVivos, Singapore and fed with standard laboratory diet and distilled water ad libitum. The animals were kept on a 12 h light/dark cycle at 22 ± 2°C in individually ventilated caging system with 50-65% humidity in the Biological Resource Centre, A-Star, Singapore. |
| Wild animals | No wild animals were used in the study. |
| Field-collected samples | No field collected samples were used in the study. |
| Ethics oversight | All animal experiments were carried out in accordance with ethical guidelines and approved by the Institutional Animal Care and Use Committee (IACUC), Biological Resource Center (BRC) A*STAR. |

Note that full information on the approval of the study protocol must also be provided in the manuscript.

## ChIP-seq

### Data deposition

- [x] Confirm that both raw and final processed data have been deposited in a public database such as GEO.
- [x] Confirm that you have deposited or provided access to graph files (e.g. BED files) for the called peaks.

Data access links
*May remain private before publication.*

https://www.ncbi.nlm.nih.gov/geo/query/acc.cgi?acc=GSE133183

Files in database submission

ChIP-seq files under GSE133183:
GSM3901517 HAP1_WT_H3K27me3_rep1_ChIPSeq
GSM3901518 HAP1_WT_H3K27me3_rep2_ChIPSeq
GSM3901519 HAP1_WT_H3K4me3_rep1_ChIPSeq
GSM3901520 HAP1_WT_H3K4me3_rep2_ChIPSeq
GSM3901521 HAP1_WT_H3K27ac_rep1_ChIPSeq
GSM3901522 HAP1_WT_H3K27ac_rep2_ChIPSeq
GSM3901523 HAP1_WT_rep1_IgG_ChIPSeq
GSM3901524 HAP1_WT_rep2_IgG_ChIPSeq
GSM4817452 K562_DMSO_H3K27me3_rep1_ChIPSeq
GSM4817453 K562_DMSO_H3K27me3_rep2_ChIPSeq
GSM4817454 K562_DMSO_H3K27ac_rep1_ChIPSeq
GSM4817455 K562_DMSO_H3K27ac_rep2_ChIPSeq
GSM4817456 K562_DMSO_IgG_rep1_ChIPSeq
GSM4817457 K562_DMSO_IgG_rep2_ChIPSeq
GSM4817458 K562_5µMGSK343_H3K27me3_rep1_ChIPSeq
GSM4817459 K562_5µMGSK343_H3K27me3_rep2_ChIPSeq
GSM4817460 K562_5µMGSK343_H3K27ac_rep1_ChIPSeq
GSM4817461 K562_5µMGSK343_H3K27ac_rep2_ChIPSeq
GSM4817462 K562_5µMGSK343_IgG_rep1_ChIPSeq
GSM4817463 K562_5µMGSK343_IgG_rep2_ChIPSeq
GSM4817484 K562_CRISPR_EV_H3K27me3_rep1_ChIPSeq
GSM4817485 K562_CRISPR_EV_H3K27me3_rep2_ChIPSeq
GSM4817486 K562_CRISPR_EV_H3K27ac_rep1_ChIPSeq
GSM4817487 K562_CRISPR_EV_H3K27ac_rep2_ChIPSeq
GSM4817488 K562_CRISPR_EV_IgG_rep1_ChIPSeq

GSM4817489 K562_CRISPR_EV_IgG_rep2_ChIPSeq
GSM4817490 K562_CRISPR_IGF1KO_H3K27me3_rep1_ChIPSeq
GSM4817491 K562_CRISPR_IGF1KO_H3K27me3_rep2_ChIPSeq
GSM4817492 K562_CRISPR_IGF1KO_H3K27ac_rep1_ChIPSeq
GSM4817493 K562_CRISPR_IGF1KO_H3K27ac_rep2_ChIPSeq
GSM4817494 K562_CRISPR_IGF1KO_IgG_rep1_ChIPSeq
GSM4817495 K562_CRISPR_IGF1KO_IgG_rep2_ChIPSeq

Genome browser session
(e.g. UCSC)

hg19

## Methodology

Replicates

All ChIP-seq experiments have two biological replicates and are pooled together in the peak calling process.

Sequencing depth

AccessionNumber name totalReads UniqueMappedReads PairEndReads
GSM3901517 HAP1_WT_H3K27me3_rep1_ChIPSeq 53778102*2 61937244 151*2
GSM3901518 HAP1_WT_H3K27me3_rep2_ChIPSeq 65233271*2 69784674 151*2
GSM3901519 HAP1_WT_H3K4me3_rep1_ChIPSeq 55095302*2 61093198 151*2
GSM3901520 HAP1_WT_H3K4me3_rep2_ChIPSeq 115277267*2 125585250 151*2
GSM3901521 HAP1_WT_H3K27ac_rep1_ChIPSeq 50216751*2 57131138 151*2
GSM3901522 HAP1_WT_H3K27ac_rep2_ChIPSeq 74419685*2 81886150 151*2
GSM3901523 HAP1_WT_rep1_IgG_ChIPSeq 78337587*2 57225816 151*2
GSM3901524 HAP1_WT_rep2_IgG_ChIPSeq 106478911*2 73624602 151*2
GSM4817452 K562_DMSO_H3K27me3_rep1_ChIPSeq 115344250*2 186372588 151*2
GSM4817453 K562_DMSO_H3K27me3_rep2_ChIPSeq 123090127*2 199052624 151*2
GSM4817454 K562_DMSO_H3K27ac_rep1_ChIPSeq 127553023*2 208899150 151*2
GSM4817455 K562_DMSO_H3K27ac_rep2_ChIPSeq 119154127*2 196661246 151*2
GSM4817456 K562_DMSO_IgG_rep1_ChIPSeq 106233672*2 174631622 151*2
GSM4817457 K562_DMSO_IgG_rep2_ChIPSeq 105417657*2 171896920 151*2
GSM4817458 K562_5μMGSK343_H3K27me3_rep1_ChIPSeq 115261659*2 181965172 151*2
GSM4817459 K562_5μMGSK343_H3K27me3_rep2_ChIPSeq 103912042*2 164268052 151*2
GSM4817460 K562_5μMGSK343_H3K27ac_rep1_ChIPSeq 74534091*2 122664054 151*2
GSM4817461 K562_5μMGSK343_H3K27ac_rep2_ChIPSeq 119154127*2 180720298 151*2
GSM4817462 K562_5μMGSK343_IgG_rep1_ChIPSeq 106233672*2 147922186 151*2
GSM4817463 K562_5μMGSK343_IgG_rep2_ChIPSeq 105417657*2 192687150 151*2
GSM4817484 K562_CRISPR_EV_H3K27me3_rep1_ChIPSeq 78009662*2 64158850 151*2
GSM4817485 K562_CRISPR_EV_H3K27me3_rep2_ChIPSeq 80354900*2 82820076 151*2
GSM4817486 K562_CRISPR_EV_H3K27ac_rep1_ChIPSeq 72090945*2 63679490 151*2
GSM4817487 K562_CRISPR_EV_H3K27ac_rep2_ChIPSeq 88418583*2 67783106 151*2
GSM4817488 K562_CRISPR_EV_IgG_rep1_ChIPSeq 78357004*2 49999232 151*2
GSM4817489 K562_CRISPR_EV_IgG_rep2_ChIPSeq 76427307*2 70767740 151*2
GSM4817490 K562_CRISPR_IGF1KO_H3K27me3_rep1_ChIPSeq 216373640*2 218611334 151*2
GSM4817491 K562_CRISPR_IGF1KO_H3K27me3_rep2_ChIPSeq 87358788*2 83379448 151*2
GSM4817492 K562_CRISPR_IGF1KO_H3K27ac_rep1_ChIPSeq 75968130*2 69472566 151*2
GSM4817493 K562_CRISPR_IGF1KO_H3K27ac_rep2_ChIPSeq 102566073*2 132184726 151*2
GSM4817494 K562_CRISPR_IGF1KO_IgG_rep1_ChIPSeq 124731490*2 114582398 151*2
GSM4817495 K562_CRISPR_IGF1KO_IgG_rep2_ChIPSeq 196574738*2 150543650 151*2

**Note: The number of uniquely mapped reads are calculated by `samtools view -bh -q 30 -f 3 -F 2316`, meaning reads that are properly paired and filter out non-primary alignments with a mapping score > 30.

Antibodies

H3K27me3 (C36B11, Cell Signaling Technologies)
H3K4em3 (#ab8580, Abcam)
H3K27ac (#ab4729, Abcam)
mouse IgG (#sc-2025, Santa Cruz)

Peak calling parameters

MACS2 (2.1.0.20150731) using option '-q 0.01' for H3K27ac and H3K4me3 and '--broad --broad-cutoff 0.1 -q 0.05' for H3K27me3.

Data quality

name TotalPeak PeakFC5FDR5
HAP1_WT_H3K27me3_ChIPSeq 36794 1726
HAP1_WT_H3K4me3_ChIPSeq 19187 19016
HAP1_WT_H3K27ac_ChIPSeq 8436 8283
K562_DMSO_H3K27me3_ChIPSeq 100784 3647
K562_DMSO_H3K27ac_ChIPSeq 64964 63101

K562_5µMGSK343_H3K27me3_ChIPSeq 49122 2789
K562_5µMGSK343_H3K27ac_ChIPSeq 56506 54288
K562_CRISPR_EV_H3K27me3_ChIPSeq 108147 7979
K562_CRISPR_EV_H3K27ac_ChIPSeq 39215 37904
K562_CRISPR_IGF1KO_H3K27me3_ChIPSeq 124157 14268
K562_CRISPR_IGF1KO_H3K27ac_ChIPSeq 50613 48241

** Note: PeaksFC5FDR5 column states the number of peaks with FDR < 5% (qvalue < 0.05) and fold change > 5. H3K27me3 histone modification is pervasive in the genome, and thus the enrichment for this mark is not very sharp. Applying a fold change threshold of 5 will filter out most of the peaks.

Software

MACS2 (2.1.0.20150731) using option '-q 0.01' for H3K27ac and H3K4me3 and '--broad --broad-cutoff 0.1 -q 0.05' for H3K27me3.
